# Predicting ionic conductivity of sulfide glass solid-state electrolytes using max-flow methodology

**DOI:** 10.1038/s41467-026-77363-w

**Published:** 2026-08-31

**Authors:** Matteo Pegoraro, Rasmus Christensen, Søren S. Sørensen, Anders R. Lindbjerg, Tao Du, Yossi Bokor Bleile, Anne Marie Svane, Christophe A. N. Biscio, Lisbeth Fajstrup, Morten M. Smedskjaer

**Affiliations:** 1 Department of Mathematical Sciences, Aalborg University, Aalborg, Denmark; 2 Department of Chemistry and Bioscience, Aalborg University, Aalborg, Denmark; 3 Institute of Science and Technology Austria, Klosterneuburg, Austria; 4 The School of Mathematics and Statistics, University of Sydney, Sydney, Australia

**Keywords:** Applied mathematics, Batteries, Computational methods, Glasses

## Abstract

Disordered materials such as glasses are promising candidates as solid-state electrolytes for batteries, but they tend to exhibit low ionic conductivity and current methods for investigating and predicting the ionic conductivity of electrolyte materials are inefficient. Here, we propose a framework that summarizes the topological properties of the electrolyte material’s atomic structure with a weighted graph based on periodic boundary conditions. We then compute a variation of the max-flow algorithm, a commonly used model in continuum models of transportation and liquid flow, on this summary and show that it is compatible with the simulated periodic atomic structures. Using molecular dynamics simulations of various glassy solid-state electrolyte families including both mobile lithium and sodium ions, we validate the approach by showing a positive correlation between the area-normalized maximum flow predicted from the static glass structures and the ionic conductivity as obtained from the simulations, especially in the case of high-conductivity sulfide glass electrolytes. We envision that the developed methodology, which only relies on static atomic structure, will accelerate the discovery of highly conductive sulfide glassy electrolytes for high-performance all-solid-state batteries.

## Abstract (web summary)

Here authors present a max-flow method that uses static glass structures to predict how fast ions move, especially in sulfide glasses, helping to accelerate the discovery of high-performance electrolyte materials for batteries.

## Introduction

The development of high-performance rechargeable ion batteries has played a pivotal role in many facets of modern society, including transportation, communication, and the transition to renewable energy production. Unfortunately, the liquid electrolytes employed in conventional ion batteries are associated with serious safety concerns^1^. Furthermore, the chemical and electrochemical reactivity of these electrolytes limits the potential for employing high-performance Li/Na metal anodes, which tend to form dendrites during repeated charging/discharging. These issues have sparked an interest in developing safer and more stable alternatives in the form of solid-state electrolytes (SSEs), which also offer an opportunity for higher energy density^2^. Glassy materials are promising candidates for this purpose as they exhibit isotropic ionic conductivity, easy compositional tuning, and the ability to be molded into bulk shapes^3^. In this context, lithium sulfide glasses such as Li_2_S-P_2_S_5_^4^, Li_2_S-SiS_2_^5^, Li_2_S-P_2_S_5_-Li_3_N^6^, Li_2_S-B_2_S_3_-LiI^7^, and Li_2_S-P_2_S_5_-GeS_2_^8^ have garnered significant attention owing to their high ionic conductivity of ~10^−3^ S cm^−1^ at ambient temperature, approaching the conductivity of liquid electrolytes (10^−3^ to 10^−2^ S cm^−1^). The hygroscopic nature of sulfide glasses, nevertheless, makes them unstable in air. Accordingly, more stable oxide glasses such as LiSiPON^9,10^, Li_2_SO_4_-Li_2_CO_3_^11^, and Li_2_O-B_2_O_3_-P_2_O_5_^12^ have also been studied as potential SSE materials even though these glasses present lower ionic conductivities in the range of 10^-7^ to 10^−5^ S cm^−1^. In response to growing concerns of lithium scarcity, efforts have been made to develop glassy SSEs for application in sodium-based ion batteries. The sodium glasses such as Na_2_S-P_2_S_5_^13^, Na_2_S-SiS_2_^14,15^, and Na_2_S-P_2_S_5_-SiS_2_^16^ typically present lower ionic conductivities compared to their lithium counterparts (in the range of 10^-6^−10^-4^ S cm^-1^) but seem to possess superior glass-forming abilities.

At present, the development of suitable glassy SSEs is complex, as it needs to consider multiple factors simultaneously, such as ionic conductivity, chemical stability, mechanical integrity, stability of electrolyte-electrode interfaces, and material availability^17–19^. The principal shortcoming of glassy SSEs remains their comparatively low ionic mobility, and thus significant efforts have been devoted to the discovery of glass compositions that exhibit high ionic conductivity (≥10^−3^ S cm^−1^). This search has been dominated by trial-and-error experiments based on previous experience, as generalizable structure-property relations that could guide the search remain elusive^20,21^. Characterization of the conduction behavior of electrolyte materials is both expensive and time consuming. Over the years, several models for predicting conductivity have been developed. Notable early examples include the associated solutions model^22^, weak electrolyte model^23^, and the Anderson and Stuart model^24^. More recent modeling efforts include the work of Wilkinson et al. in which the ionic diffusivity of a glass is predicted from its non-equilibrium viscosity^25^. Moreover, several studies have employed the so-called Bond Valence method to calculate conduction pathways, which can subsequently be correlated to the material’s ionic conductivity^26,27^. In recent years, attempts have also been made to employ machine learning models trained on ionic conductivity data to predict glass compositions with favorable conduction behavior^28^. Similarly, there are also attempts to optimize existing techniques, such as the nudged-elastic-band method^29^, to develop other descriptions, for example, based on topological graphs of the atoms or electron densities, Voronoi face analysis, and freezing of the host-network to allow for computationally cheaper molecular dynamics steps in the so-called “pin-ball” model^30^. However, these previous workflows commonly focus on implementations for crystalline materials with simpler diffusion pathways compared to amorphous systems. In the latter, the diffusion space generally needs to be characterized by a statistically much broader range of possible pathways, which, in turn, requires much larger simulation cells, rendering many existing methods infeasible.

**Fig. 1:**
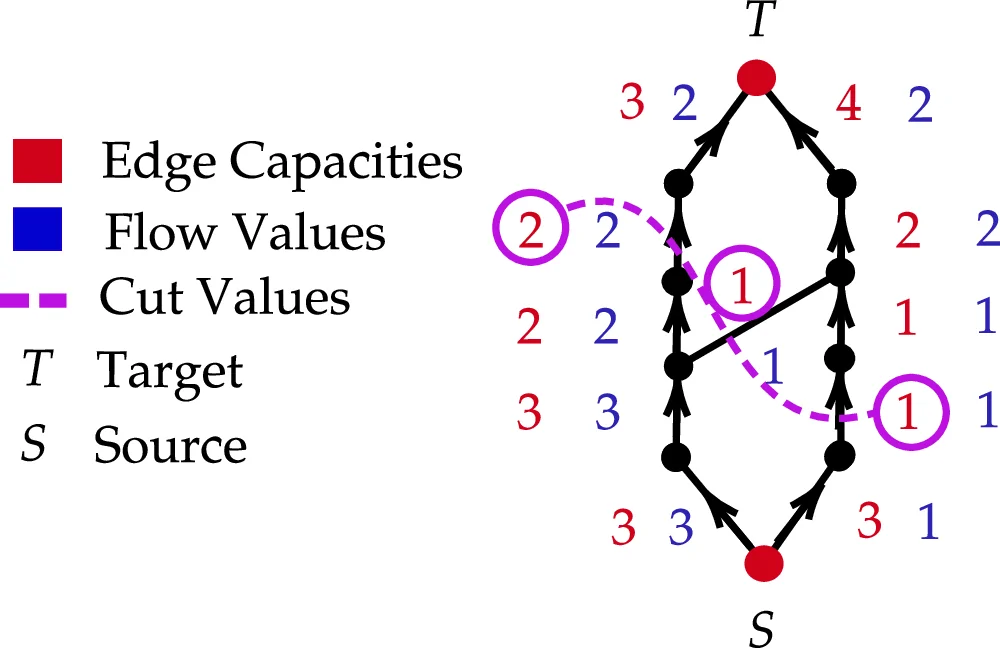
Schematic of the maximum flow method. *S* is the source, and *T* is the target (sink). For each edge, the maximum capacity and the maximum-flow solution are shown in red and blue, respectively. The purple line represents a minimum-cut solution that separates the source and target.

Despite these efforts, the study of glassy SSEs still relies almost exclusively on expensive molecular dynamics simulations and experimental measurements. Thus, to enable a more diverse screening of potential glassy SSEs, and extract insights into the relationship between atomic-scale glass structure and ionic conductivity, we propose to analyze ion diffusivity in glassy SSEs based on combinatorial optimization^31^. Specifically, we employ flow networks and maximum flow to investigate this relationship. Flow networks are a well-established concept within the field of optimization modeling, which emerged as a means of optimizing the flow of goods through the railway systems^32^. Over the years, they have been successfully applied to a wide range of fields, including modeling of electrical currents in circuitry, fluids in pipelines, and permeability of porous materials^33,34^. Here, we develop the maximum flow method for use at the atomic scale. When applying flow networks to a modeling task, the medium to be modeled is represented as a source and a sink separated by a network of vertices and connecting edges as shown in Fig. 1. Assigned to each edge is a capacity, corresponding to the maximum flow allowed through that specific edge, and the maximum flow of the network is the maximum total flow from source to sink that the network can support. From the max-flow min-cut theorem^35^, the maximum flow is found by identifying the minimum cut. We note that a cut is a subset of edges of the network whose removal makes it impossible to go from the source to the sink (see the edges intersected by the dashed purple line in Fig. 1). A minimum cut is found by minimizing the summed capacity of the edges that constitute the cut.

In this work, we represent atomistic models of glassy SSEs as flow networks and investigate the efficacy of employing the maximum flow through these networks as a measure of ion diffusivity. We do so by summarizing the material's topological properties as a weighted graph under periodic boundary conditions, on which a variation of max-flow is computed that is compatible with periodicity. In turn, we do this by generalizing two previous studies that used max-flow to describe transportation properties in porous materials^33,34^, but these do not consider periodic boundary conditions on the simulation box, and so are not suitable in this setting. We then test the methodology on selected families of archetypical glassy SSEs or glasses with known highly diffusive alkali ions, namely combinations of either Li_2_X or Na_2_X with SiX_2_, P_2_X_5_, B_2_X_3_, or GeX_2,_ with X being either oxygen (O) or sulfur (S). Further, we have simulated archetypical SSE glasses, i.e., Li_2_S-P_2_S_5_ (LiPS), Li_2_O-B_2_O_3_-P_2_O_5_ (LiBP), and Na_2_S-SiS_2_ (NaSiS). In detail, we chose LiPS as it is a typical example of a lithium sulfide glass, which has been heavily studied and whose diffusion mechanisms thus are relatively well understood; NaSiS as an example of a sodium conducting electrolyte; and LiBP as an example of a structurally rigid electrolyte, consisting of an interconnected network^36^. Since experimental characterization of atomic configurations of glass materials is still out of reach, structures of glasses from the families are generated using molecular dynamics (MD) using a combination of both classical and machine-learning-based potentials. These structures are then characterized by simulating the diffusion process and calculating the ionic conductivity at different temperatures. After this, we use the atom positions in the simulated glass systems as input to generate network representations that form the basis for calculating flow. Finally, we compute the maximum flow values for these (simulated) glasses. These flows are compared to the calculated room-temperature conductivity of the diffusing Li or Na ions, revealing a positive correlation between the two values for the high-conductivity sulfide glass electrolytes. As such, the present validation is focused on inorganic glass electrolytes in which the ion/network structure is effectively static on the ion-transport timescale. Further, we note how dynamically disordered systems, such as polymeric or hybrid electrolytes, where ion-hosted network rearrangements may be coupled to ion transport, require separate validation before the max-flow descriptor can be applied.

## Results

### Maximum flow pipeline

**Fig. 2:**
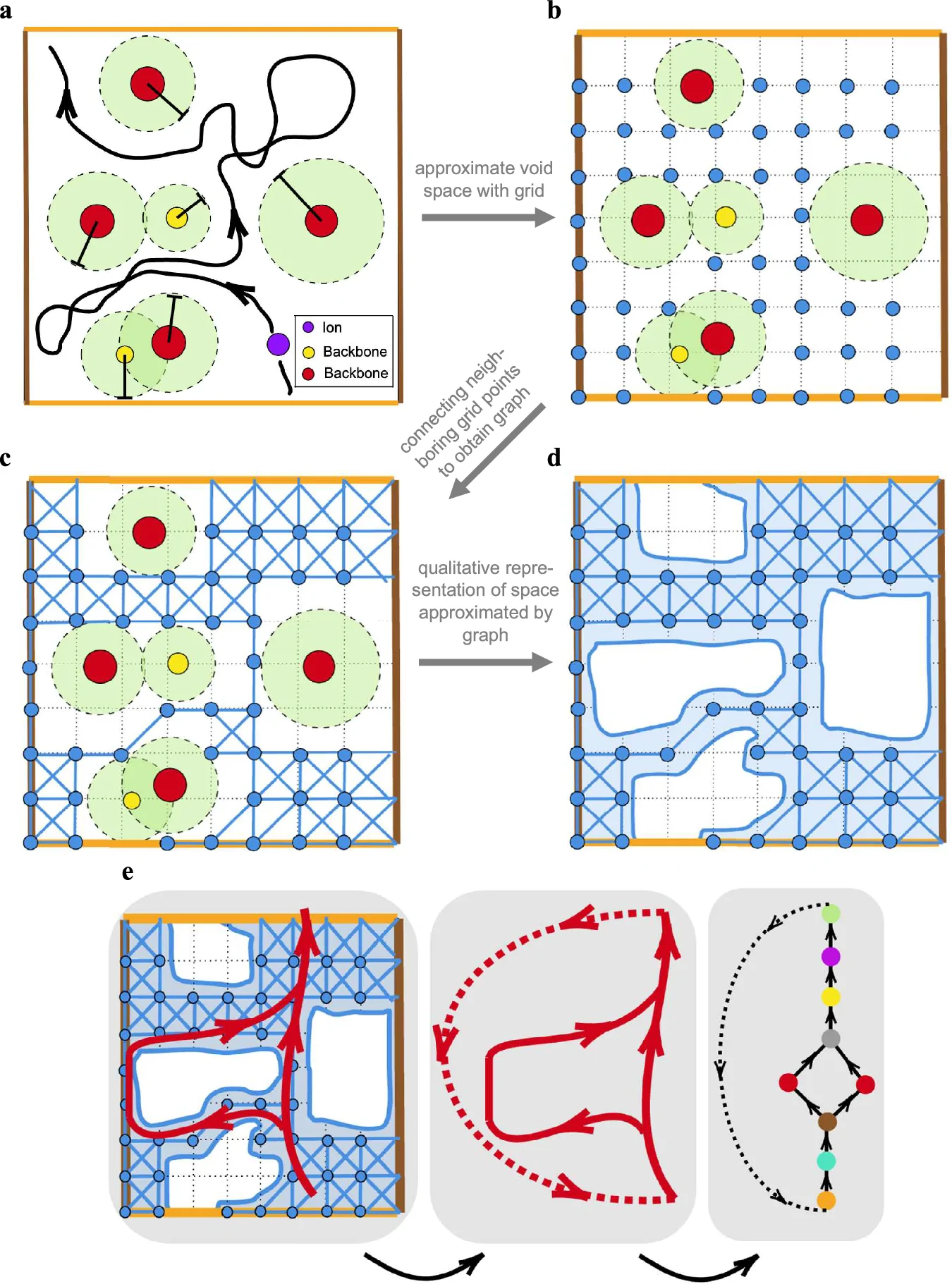
Visual representation of the pipeline employed to proceed from the glass structure data to the map between graphs used to compute the circular max-flow. **a** Qualitative 2D representation of the data, with red and yellow circles representing the backbone atoms of the glass and the purple atoms representing lithium or sodium ions, which move following a self-diffusion process. Circles/radii around the backbone atoms represent the neighborhood of the atoms where we do not observe diffusing ions across time. The void space through which ions flow is represented by the area outside the light green zones. **b** Discrete representation (grid approximation) of the void space, which is needed to analyze its topological properties via max-flow. First, a fine grid is fitted inside the void space. **c** Graph approximation of the void space. To describe the connectivity properties of the void space, we connect neighboring points on the grid, yielding a graph approximation of the void space. **d** Qualitative representation of the “continuous” topological space that we approximate with the graph discretization. **e** Construction of the mapper graph. Upon choosing a direction for the ions to “flow” in the void space, we capture the topology of the void space with a graph along that direction.

The main steps of the analysis pipeline for calculating maximum flow can be summarized as (see Fig. 2): (1) obtain a representation of the space where the ions can move; (2) discretize this representation with a finite grid of points and with edges connecting nearby points, allowing computational modeling of this void space; (3) build a simplified version of this discretized void, i.e., divide the grid in areas/sockets, and obtain a new graph by collapsing each socket to a point, and using the edges to describe tunnels/connectivity between the different sockets; and (4) use this simplified model to compute a notion of max-flow. In other words, we use a graph to build a simplified model of the void space by aggregating points belonging to the same sockets and describing relationships between sockets with edges. Then, interpreting the edges as tunnels between sockets, this describes the number of atoms that this system of tunnels can accommodate, i.e., representing a geometric counterpart of the atom diffusivity. In the following, we provide more details on these steps and refer the reader to the Methods and Supplementary Methods sections (and associated Supplementary Figs. 1–6) for a finer and formal description as well as an estimation of the computational cost of running the max-flow algorithm (see Supplementary Text).

First, we simulate the self-diffusion of ions, noting that since the simulations are carried out with periodic boundary conditions, we consider them as being present in a box whose sides are coherently identified. Then, we separate these simulated systems into two parts, namely the mobile diffusing ions and a fixed backbone (Fig. 2a). Here, we consider Li^+^ and Na^+^ to be diffusing ions while everything else is treated as being part of the static backbone, motivated by the significantly lower mean squared displacements of the backbone atoms (factors of one to four orders of magnitude, see examples in Supplementary Figs. 7–9). Accordingly, the descriptor is expected to be most meaningful when the backbone structure is effectively static on the ion-transport timescale, i.e., a single time-averaged backbone provides a representative structural environment for ion migration. For each atom in the static backbone, we compute the Fréchet mean of the atom’s positions over the simulation at low temperature to get a statistically meaningful description of the backbone, with little effect from the instantaneous thermal vibrations. We can think of the Fréchet mean as the *average location* of the atom in the simulation. We develop a model for the void space, i.e., we identify the common shortest distance that the backbone interacts with the diffusing ions, from the onset of the first peak in their pair-wise radial distribution function *g*(*r*) (see Supplementary Figs. 10–12). This can be thought of as the largest sphere that can be placed at each atom’s Fréchet mean before it intersects with a diffusing ion during the simulation (Fig. 2b). The void space is now given by the complementary of the union of these spheres for each atom in the backbone, as described in detail in Supplementary Methods (section 4). Thus, local ion/backbone interactions enter the present descriptor indirectly through the radial distribution function-derived definition of the excluded volume. Once this void space has been fixed, the subsequent max-flow calculation is geometric/topological in nature and quantifies the size and connectivity of the volume accessible for the ions, rather than explicitly encoding ion-binding strengths or migration-energy barriers. To enable efficient computations, we discretize the void space using a grid as shown in Fig. 2b. We provide a thorough discussion on the fundamental computational cost of the max-flow method in the Supplementary Text.

We then encode connectivity information at each grid point *p* by connecting it to the neighboring points in the 3×3×3 cube centered at *p* (see Fig. 2c for a two-dimensional representation). Each point is then connected with at most 26 other points (each cube contains 27 points, one of which, the barycenter of the cube, is the point itself). Assuming the grid is fine enough (see Supplementary Methods section 4 for more details), we obtain a graph that can capture topological information of the void space (Fig. 2d). The next step is to obtain a simplified graph, called a mapper graph, which keeps track of the connected components in horizontal slices and how these are connected vertically. The mapper graph presents a greatly reduced number of edges and vertices. Figure 2e shows a visual representation (a more detailed version is given in Supplementary Fig. 4) and the formal construction is described in Supplementary Methods section 5. As a byproduct of the mapper construction, we also obtain (1) weights on the edges, indicating the maximum number of ions that can flow through each edge, and (2) a map between graphs (Supplementary Fig. 5), which we use to compute the circular max-flow, i.e., the maximum number of ions that can flow through the mapper graph. For instance, in the outer graph in Supplementary Fig. 5, the value of the circular max-flow is 2. While we do not explicitly finetune the weights of the edges, we note that this is possible and allows for more complex descriptions of chemistries.

### Model implementation

As input and validation of our analysis pipeline, for each structure, we need: (1) a snapshot representation as a basis for generation of flow networks; and (2) ion trajectories recorded during self-diffusion to enable calculation of dynamical properties for comparison with the maximum flow. In the present work, we avoid ab initio MD simulations based on density functional theory as, although they are accurate, they do not scale well to large numbers of atoms (several thousands) and long simulations (nanoseconds), which are needed in the present study^37^. Instead, we here employ a combination of purely classical potentials for screening various compositions within three different families as well as a wider range of oxide and sulfide compositions based on a recently developed machine-learning interatomic potential^38^ (see full list of compositions in Supplementary Table 1). The glass compositions were designed to access different structural features (e.g., varying coordination number and connectivity) within each family. Our focus is on demonstrating that our analysis pipeline is suitable for the task at hand and that we are able to observe and discuss general behaviors of the systems. We thus treat the simulated structures as computer glasses to establish the connection between max-flow and ionic conductivity, i.e., perfect agreement with experimental observables is not necessary for model validation. We generated the various compositions of modified oxide and sulfide glasses by simulating the melt-quench process. For details on the simulations, we refer readers to the Methods section.

### Correlating ionic conductivity and max-flow

**Fig. 3:**
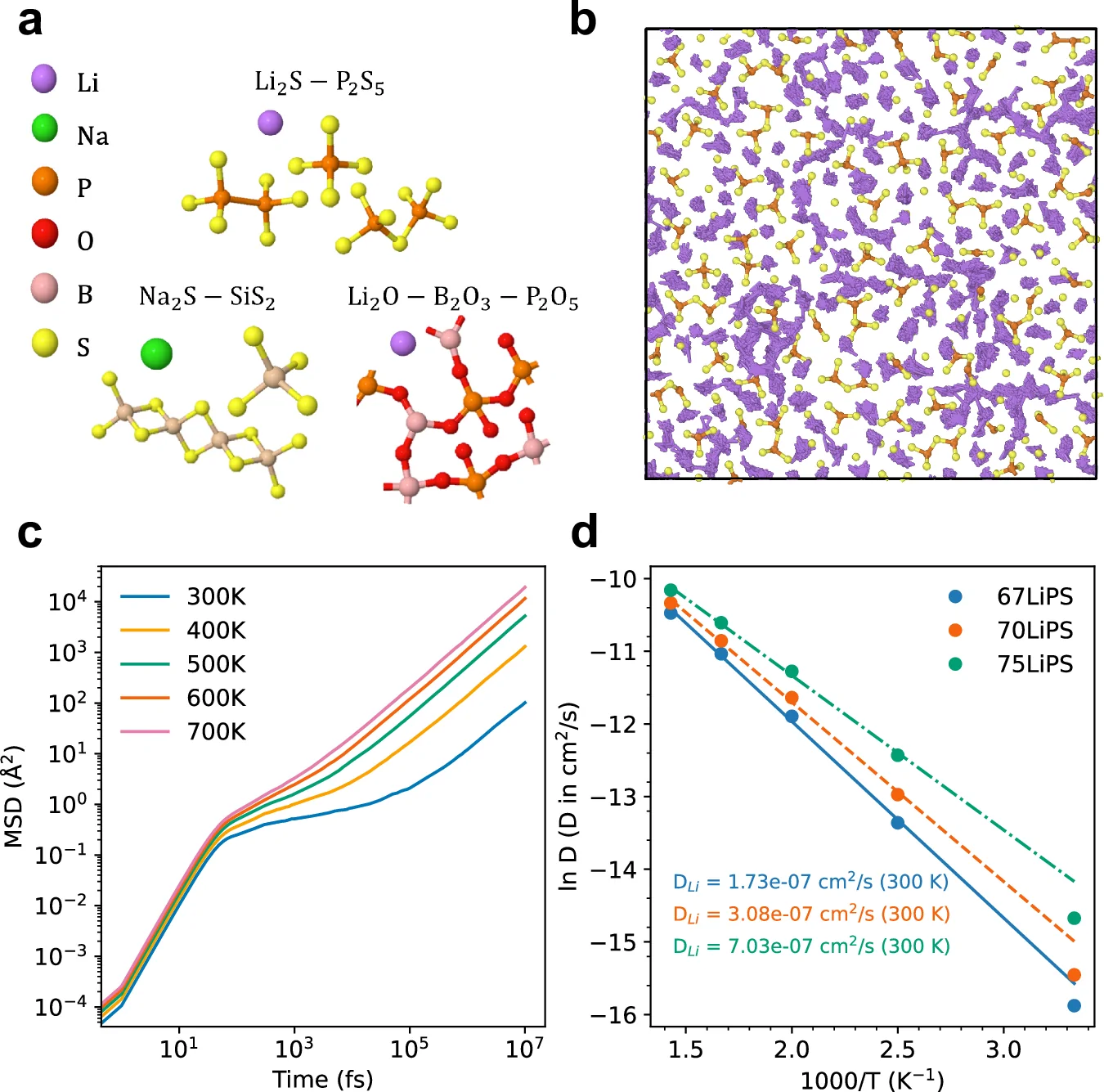
Lithium and sodium diffusion in glassy electrolytes. **a** Structural components of Li_2_S-P_2_S_5_ (LiPS) Na_2_S-SiS_2_ (NaSiS), and Li_2_O-B_2_O_3_-P_2_O_5_ (LiBP) glasses. **b** Visualization of Li trajectories (purple traces) in the 70LiPS glass sampled over 10 ns at 300 K. **c** MSD of Li ions in 70LiPS as a function of time at various temperatures. **d** Arrhenius plot of the diffusion coefficients of glasses in the LiPS system sampled at elevated temperatures. The lines represent fits to the high-temperature data (i.e., with the 300 K data point excluded). Source data are provided as a Source Data file.

Representations of some of the typical structural building units found across the simulations are shown in Fig. 3a. In addition to the use of the simulated glass structures for the generation of network representations of the glasses, these structures are also used as a basis for the simulation of dynamic properties of the glassy electrolytes, by sampling the self-diffusion trajectories of the Li or Na ions. We find that the diffusing ions in the LiBP glasses are generally able to get closer to the atoms of the backbone (Li-O minimum distance of ~1.7 Å) than in the LiPS glasses (Li-S minimum distance of ~2.1 Å) and NaSiS glasses (Na-S minimum distance of ~2.4 Å), as seen from the partial radial distribution functions of the diffusing ions (Supplementary Figs. 10–12). Figure 3b shows an example of the trajectory of Li-ions in a small section of the 70Li_2_S-30P_2_S_5_ (LiPS70) glass sampled over 10 ns at 300 K. Details of these calculations are outlined in the Methods section, following a common strategy of calculating the mean square displacement (MSD) of each elemental type at varying temperatures (Fig. 3c). The slope of the MSD in the diffusive regime is then directly correlated to the diffusion constant.

Due to limited diffusion at room temperature, it is not feasible to calculate dynamic properties from room-temperature simulations. This is due to the inability of ions to reach the MSD diffusive regime within the timeframe of the simulations (see Supplementary Figs. 7–9). Hence, while we have attempted to calculate the diffusivity at 300 K (as shown in Fig. 3c, d), we indeed extrapolate the room-temperature diffusivity from the simulations at elevated temperatures, i.e., above 300 K. Figure 3d shows an example of such an extrapolation for the self-diffusion coefficient of Li^+^ in the three Li_2_S-P_2_S_5_ glasses (plots pertaining to the Li_2_O-B_2_O_3_-P_2_O_5_ and Na_2_S-SiS_2_ glasses are shown in Supplementary Figs. 13 and 14). The room-temperature self-diffusion coefficients and ionic conductivities we obtain in this way correspond well with previously simulated results^20,39,40^. Furthermore, the compositional trends from experimental measurements of the ionic conductivity of these glasses correspond well with those of our simulations (Supplementary Table 2), although the absolute values differ by a few orders of magnitude^12,20,41^. Notably, the latter is often observed in molecular dynamics simulations of glassy electrolytes^21^ and is commonly attributed to a variety of effects, including shortcomings of the used force field, inabilities of the simulation cell to describe all modes of ionic transport, as well as the breakdown of ergodicity in the glassy state^42–44^. Ultimately, it is a separate problem, which is beyond the scope of the present work.

**Fig. 4:**
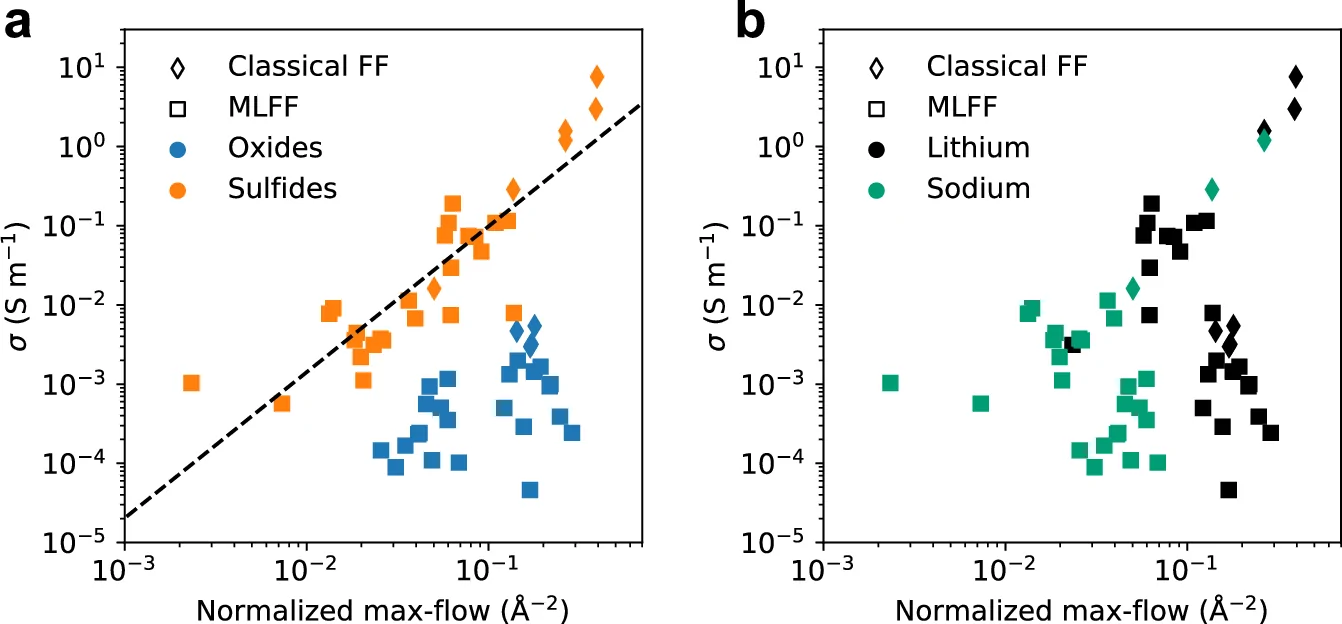
Predicting ionic conductivity from maximum flow. Calculated room temperature ionic conductivity of glasses plotted as a function of normalized max-flow as calculated from the static glass structures. Results are categorized based on (**a**) glass family [blue: oxides; orange: sulfides] and (**b**) diffusing ion [black: lithium; green: sodium]. Diamonds denote glasses simulated by a classical force field, and squares denote glasses simulated by a machine-learning force field (MLFF). We refer to the Methods for a detailed description of this distinction. Note that the axes of the plot are on a logarithmic scale, and the black dashed line in panel (**a**) represents a guide for the eye (linear fit to the data). Source data are provided as a Source Data file.

Next, we investigate the correlation between the simplistic max-flow measure and the more computationally demanding ionic conductivity. To this end, we first note that ionic conductivity is invariant under cell dimension changes while max-flow is not. That is, the flow scales linearly with the cross section of the simulation box under study, and we thus define a max-flow that is normalized by the simulation box side area (which in all our simulations is equivalent for each composition in all directions). We have confirmed that this normalization provides invariance of the max-flow in our implementation. Then, Fig. 4 shows the extrapolated room-temperature conductivity of the diffusing ions (Na^+^ or Li^+^) as a function of the calculated maximum flow from the static glass structure. We observe a strong positive correlation between normalized max-flow and ionic conductivity in the case of sulfide glass electrolytes (Fig. 4a), across both sodium and lithium charge carriers (Fig. 4b). This is interesting considering that the static glass structures used to calculate maximum flow do not contain any explicit information about the ion dynamics. However, we also note that there is no clear correlation for the lower-conductivity oxide glass electrolytes. This may be due to oxygen’s smaller ionic radii but similar charge as sulfur, creating more directionality in local bonding between oxygen and the fast-moving ions. In turn, this may create a stronger correlation between diffusion and the unique local environment of the ion compared to the more ionic bonding of the sulfides. Finally, we note that while Fig. 4 shows the correlation between ionic conductivity and normalized max-flow, the correlation mostly persists when using diffusivity, which does not take the number of Na^+^ or Li^+^ charge carriers into account (Supplementary Fig. 15) but rather provides the average resistance of an ion to diffuse.

## Discussion

The results of our proposed max-flow algorithm show that the conductivity of ions in glass electrolytes is strongly influenced by the ion-conduction pathways in the materials, both in the number of paths and their volumes. We can differentiate between the diffusivity of sodium and lithium ions in especially high-conductivity sulfide systems based only on snapshots of the static structure. That is, ionic conductivity in sulfide glasses features a strong correlation with max-flow, while this is not the case for oxide glasses, which may be due to the more directional Li/Na-O bonding compared to the Li/Na-S bonding in the sulfides. Interestingly, both glass systems seem to exhibit a maximum ionic conductivity for a given max-flow, which suggests that the max-flow method is suitable as a screening parameter in the search for high-conductivity electrolyte materials, particularly for highly conductive sulfide glasses. We note that systems with similar ion-accessible void connectivity, but different ion-binding strengths or activation barriers may exist, which would result in similar max-flow values. However, we expect this to be a rare case, since the accessible void connectivity should be indirectly reflected in the static structure and the related radial distribution functions, which, in turn, are used to derive the accessible volume.

To this end, we note that while the max-flow algorithm also incurs computational cost, it is lower than computing MSDs of ions at various temperatures and over long timescales. As an example, the estimation of the ionic conductivity directly from MD simulations using the machine learning force field (MLFF) has a cost of ~1500 CPU core-hours (~12 hours of wall time on a 128-core CPU) for each composition. In comparison, the max-flow calculations have a cost of ~5 CPU core-hours per composition, yielding a computational speedup of around two to three orders of magnitude (which is notably highly reliant on the computational cost of the used MD force field). Furthermore, as the max-flow algorithm only relies on the static atomic structures, the algorithm is agnostic to the chemical systems and can be widely applied for the study of ion conduction in glasses, relying only on the availability of high-quality atomic structures (and reliable force fields) and the ability to reliably define a backbone. We envision that, in future work, the method could be used for identifying structural bottlenecks in ion diffusion^45^ and diffusion through glass/crystal interfaces in glass-ceramic electrolytes^46^ as well as for use in systems with more complex backbones, including glasses with mixed-anion backbones (e.g., mixtures of sulfides, oxides, halides) and polymeric systems^47^.

A main feature of the method is that diffusion of network (backbone) atoms is not considered. While this is not a problem at room temperature for glassy electrolyte materials with glass transition temperatures typically 100-200 K above room temperature, this assumption breaks down at higher temperatures (as shown in the MSD data, see Supplementary Figs. 7–9). Furthermore, the representation of glasses as flow networks solely based on backbone structures could suggest that differences in the type of diffusing ions are not accounted for. However, as clearly shown in Fig. 4, the max-flow method is able to account for the intermediate conductivity of both lithium and sodium-containing systems and collapse both systems onto a single master curve for the sulfides. The ion diffusivity depends firstly on the force needed to induce movement in ions, which is governed by the mass of the ions and the strength of the interactions between ions and the backbone, and secondly on the space ions must traverse, which is governed by the backbone geometry. As such, we infer that the backbone geometry likely indirectly reflects the difference in size of Li^+^ vs. Na^+^ and thus the flow and ionic conductivity of the network. Lastly, we note how future fine-tuning of the topological edge-weights may allow for improvements in the predictability of the method.

## Methods

### Molecular dynamics simulations

MD simulations of the glassy electrolytes were performed using the open-source LAMMPS software. Systems from three specific glass families (LiPS, NaSiS, and LiBP) were simulated using different classical force fields, while an additional 48 glass compositions were simulated using a machine learning force field. Exact compositions of all simulated glass samples can be found in Supplementary Table 1, but follow the ratios of 2:1, 1:1, and 1:2 for network modifiers (Li, Na) to network formers (B, P, Si, Ge), charge balanced with either O or S. When simulating LiPS glasses, the force field developed and tested in ref. ^39^ was employed. This force field combines the functional forms of the Stillinger-Weber and Class II potentials. Simulation of NaSiS glasses was carried out using a modified version of the force field developed in ref. ^20^ as described in ref. ^44^. This modified force field is based on a Buckingham-Coulomb potential taken directly from ref. ^20^ except for the dispersion parameter, which was optimized in ref. ^44^ with respect to the ability of the force field to accurately reproduce experimental structural data. Finally, the force field developed in ref. ^48^ was employed for the simulation of LiBP glasses. This force field is based on a Buckingham-Coulomb potential, which was parametrized by building on the so-called Teter parameterization. For the systems simulated with the machine learning force field, we used the recently proposed GRACE-FS^38^, with D3 dispersion correction^49^, which has been specifically parameterized to cover common glass electrolyte systems and was benchmarked on such systems. We have previously used each of these force fields to study diffusion in glasses^38,40,44,50^.

We used typical melt-quenching schemes to generate the glasses as follows. For the LiPS glasses, appropriate ratios of predefined structural units (PS_4_, P_2_S_6_, P_2_S_7_, and Li) according to experimental Raman and ^31^P magic angle spinning nuclear magnetic resonance spectroscopy measurements^51^ were randomly placed in a simulated box. For all other glasses, atoms were randomly placed in the simulation box to obtain the chosen overall atomic composition. The total number of atoms included was ~8000 in the case of LiPS and NaSiS, ~9500 in the case of LiBP, and ~3000 in the case of the MLFF simulated systems. These choices were inspired by the work reported in refs. ^20^,^39^,^40^, respectively. The initial structures for the three systems were melt-quenched according to previously published quenching schemes. For the LiPS system, the structures were first melted at 3000 K in the *NVT* ensemble for 100 ps and subsequently quenched by cooling to 300 K in the *NVT* ensemble with a cooling rate of 30 K/ps. The quenched structures were relaxed for 1 ns at 300 K under both the *NVT* and *NPT* ensembles. Similarly, for the NaSiS system, the structures were first melted at 3000 K in the *NVT* ensemble for 100 ps, followed by quenching to 300 K in the *NVT* ensemble with a cooling rate of 100 K/ps and subsequently relaxed for 1 ns at 300 K in *NVT*. For the LiBP system, the structures were melted at 3000 K in the *NVT* ensemble for 200 ps and quenched to 300 K in the *NPT* ensemble at zero pressure using a cooling rate of 1 K/ps. These structures were then relaxed for 100 ps at 300 K under both the *NVT* and *NPT* ensembles. Systems simulated with the MLFF were first energy minimized, followed by a 20 ps equilibration at 3000 K (*NVT*) and then by 100 ps at 3000 K (*NPT*, zero pressure). The systems were quenched to 300 K with a cooling rate of 1 K/ps (*NPT*, zero pressure). Finally, they were relaxed for 100 ps at 300 K (*NPT*, zero external pressure) to obtain relaxed glass structures, which were sampled for 100 ps at 300 K (*NVT*). The final glass structures of the systems simulated with classical potentials were validated against experimental measurements (Supplementary Figs. 16–18), showing reasonable structural agreement. We refer the reader to ref. ^38^. For a thorough benchmarking of glass structures simulated with the MLFF.

To obtain self-diffusion data, we simulated glass structures at various temperatures by heating them to target temperatures. For classical force fields, systems were allowed to equilibrate for 1 ns before measuring the diffusion trajectory over 10 ns (all in the *NVT* ensemble), while for MLFF, 10 ps of equilibration and 1 ns for diffusion sampling were used. Self-diffusion was determined at the following temperatures depending on the potential: LiPS glasses were sampled at 300, 400, 500, 600, and 700 K; NaSiS glasses were sampled at 300, 600, 800, 1000, 1200, 1400, and 1600 K; LiBP glasses were sampled at 300, 500, 600, 650, 700, 750, and 800 K; and systems simulated with MLFF were sampled at 500, 700, 900 and 1100 K. The choices of temperature range were made with consideration of the respective softening point of the families of glasses upon which significant diffusion of all atomic species would occur.

### Dynamical properties

We calculated the dynamic properties of the simulated electrolyte glasses as follows. First, mean-square displacement (MSD) was calculated for the diffusing ions of each of the simulated glasses as,

1 $${MSD}\left(t\right)=\frac{1}{N}{\sum}_{i}^{N}{\left({{{{\boldsymbol{r}}}}}_{{{{\boldsymbol{i}}}}}\left(0\right)-{{{{\boldsymbol{r}}}}}_{{{{\boldsymbol{i}}}}}\left(t\right)\right)}^{2},$$

where *N* is the total number of diffusing ions and ${{{{\boldsymbol{r}}}}}_{{{{\boldsymbol{i}}}}}(t)$ is the Cartesian coordinates of the *i*th ion at time *t*. Then, values of MSD were used as input for the calculation of self-diffusion coefficients *D*,

2 $$D=\frac{1}{6t}\left({MSD}\left(t\right)\right).$$

Although diffusion simulations at room temperature were conducted, we chose to utilize the Arrhenius relationship of the self-diffusion coefficient and temperature as shown in Eq. (3) to extrapolate the self-diffusion coefficient at 300 K from the self-diffusion coefficients at elevated temperatures.

3 $$D={D}_{0}\exp \left(-\frac{{E}_{a}}{{k}_{B}T}\right)$$

Here, ${D}_{0}$ is the pre-exponential factor corresponding to the self-diffusion coefficient at infinite temperature, ${E}_{a}$ is the activation energy of a diffusion event, ${k}_{B}$ is the Boltzmann constant, and T is the temperature. We chose to extrapolate the self-diffusion coefficient at 300 K due to the increased uncertainty of the calculated self-diffusion coefficient, because only very small atomic displacements occur at this temperature during the diffusion trajectories.

Ionic conductivity (σ) is related to the diffusion coefficient and the concentration of mobile ions, and can be expressed as,

4 $$\sigma={ne}\mu,$$

where *n* is the number density of mobile ions, *e* is the charge of the ion, and *μ* is the mobility of the ions. The mobility (*μ*) of ions is related to their diffusion coefficient (*D*) through the Nernst-Einstein relation,

5 $$\sigma=n\frac{{\left({Ze}\right)}^{2}}{{k}_{b}T}D,$$

where *Z* is the valency of the conducting ion and *e* is the elementary charge.

Calculation of MSDs and the associated diffusion coefficients for Li and Na in the different glass systems at the sampled temperatures was performed using Bayesian linear regression via Markov-chain Monte Carlo sampling as implemented in the KINISI python package^52,53^. We note that KINISI effectively yields a result similar to classical fitting in the diffusive region of the mean-square displacement correlation, but in a statistically more robust manner.

### Maximum flow calculation

To make our problem amenable to maximum flow prediction techniques, we first represent the glasses as flow networks. An overview of the steps involved in this conversion is shown in Fig. 2. The starting point for all the glasses was a box containing mobile diffusing ions and a more stationary backbone. In the first step, the diffusing ions were removed, leaving only the backbone and channels of void space behind. Additionally, the backbone was no longer treated as being comprised of multiple individual atoms, and only the total space it occupied was considered. Finally, the void space was simplified into a series of connected nodes, with each connection weighted by the volume of the void it represented. Following this conversion, the maximum flow rate (also referred to as the flow) was calculated as the solution to an optimization problem. See details in Supplementary Methods^54–59^, Supplementary Text, Supplementary Table 3, and Supplementary Figs. 1–6.

## Supplementary information

Supplementary information

Transparent Peer Review file

## Source data

Source Data

## Data Availability

All data supporting the findings of this study are available within the article and Supplementary Information files. Source data are provided with this paper.

## Code availability

The developed code to perform the max-flow calculations is available as part of the Topological Materials Analysis (ToMA) library, which is available at Zenodo (https://doi.org/10.5281/zenodo.10781424) and Github (https://github.com/yossibokorbleile/TopologicalMaterialAnalysis), with documentation available at https://yossi.eu/toma/. To enhance usability, we have included a brief documentation on how to compile ToMA as well as a minimal working example of the code together with an example trajectory directly in the ToMA repository. LAMMPS is an open-source code available at https://lammps.sandia.gov.
