# Supplementary material for: Predicting ionic conductivity of sulfide glass solid-state electrolytes using max-flow methodology
Source: Nat Commun. 2026 Aug 31;17:10343. doi: 10.1038/s41467-026-77363-w (PMC13623887; doi:10.1038/s41467-026-77363-w)
Supplement: Supplementary file 1 [file 41467_2026_77363_MOESM1_ESM.pdf]

Supplementary Information *for*

Predicting ionic conductivity of sulfide glass solid-state electrolytes using max-flow methodology

Matteo Pegoraro[1,†], Rasmus Christensen[2,†], Søren S. Sørensen[2,†], Anders R. Lindbjerg[2], Tao Du[2], Yossi Bokor Bleile[1,3,4], Anne Marie Svane[1], Christophe A. N. Biscio[1], Lisbeth Fajstrup[1,*], Morten M. Smedskjaer[2,*]

[1] *Department of Mathematical Sciences, Aalborg University, Aalborg, Denmark*

[2] *Department of Chemistry and Bioscience, Aalborg University, Aalborg, Denmark*

[3] *Institute of Science and Technology Austria, Austria*

[4] *The School of Mathematics and Statistics, University of Sydney, Australia*

[†] These authors contributed equally to this work.

[*] Corresponding authors. E-mail: fajstrup@math.aau.dk (L.F.), mos@bio.aau.dk (M.M.S.)

Supplementary Methods

1. Notation

In the following, we establish the notation to formally present all the details of our pipeline.

We model the simulation box as a 3-dimensional flat torus $\mathbb{T}_3 = \mathbb{S}^1 \times \mathbb{S}^1 \times \mathbb{S}^1$. It is a Riemannian manifold, with the metric being given by the 2-norm of the distances on each $\mathbb{S}^1$, i.e.,

$$d\big((z_1, z_2, z_3), (x_1, x_2, x_3)\big)^2 = d_{\mathbb{S}^1}(z_1, x_1)^2 + d_{\mathbb{S}^1}(z_2, x_2)^2 + d_{\mathbb{S}^1}(z_3, x_3)^2 \,, \tag{S1}$$

where $d_{\mathbb{S}^1}$ is the metric on each circle given by the angular distance, re-scaled by the length of the corresponding side of the box used in the simulation. According to standard notation, given a point p on a smooth manifold, we use $T_p(M)$ to indicate the tangent space at the point p, $\log_p: U_p \to T_p(M)$ stands for the logarithmic map centerd in p, with U_p being a suitable open neighborhood of p, and $\exp_p: T_p(M) \to M$ being the usual exponential map centered in p. Similarly, the notation $B_r(p)$ is used to indicate open balls in a metric space (X,d), with radius r,

$$B_r(p) = \{x \in X \mid d(p, x) < r\}. \tag{S2}$$

Finally, we introduce the notation that we use to describe our data set. For a glass sample, we let $B_{t_i} = \{b_1^{t_i}, \dots, b_H^{t_i}\} \subset \mathbb{T}_3$ be the backbone atoms at the time-step t_i. Similarly, $L_{t_i} = \{l_1^{t_i}, \dots, l_N^{t_i}\} \subset \mathbb{T}_3$ is the position of the conducting ions (Li^+ or Na^+) at time t_i, for every $i = 1, \dots, K$.

2. Fixed representation of data: backbone

In order to extract the max-flow, it is important that we can reduce to a case where the backbone can be considered to be fixed. To do so, we take advantage of the fact that the backbone atoms move marginally, compared to the conducting ions, i.e., we rely on the "average" position of the former without disrupting the backbone structure. Since every atom is placed in a box with periodic boundary conditions, i.e., each atom lies on a flat torus, we cannot take the average by averaging the coordinates as in Euclidean spaces, but we employ a very popular generalization of the idea of mean to smooth manifolds, namely, Karcher means (also called Frechét means) [1]. In particular, a Frechét mean of a data set $\{x_1, \dots, x_k\}$ in a metric space (X,d) is the minimizer of the following functional,

$$x \mapsto \sum_{i=1}^{k} d(x, x_i)^2. \tag{S3}$$

In Euclidean spaces, this coincides with the sample mean. Otherwise, this provides a generalization of the idea of average to metric spaces, where we do not have a vector space structure. On smooth manifolds, it can be easily approximated with a gradient descent algorithm, relying on the fact that the gradient of the squared distance measured from a point x_0 is the vector connecting x_0 and the target point,

$$\frac{1}{2}\operatorname{grad}\big(d^2(x_0, \cdot)\big) = \log_{x_0}(\cdot). \tag{S4}$$

The difference between taking normal averages without considering periodic boundary conditions and taking Frechét means with periodic boundary conditions is exemplified, using the circle, in Supplementary Fig. 1.

For every j we obtain the average j-th atom of the backbone as,

$$\overline{b_j} = \min_{b\in\mathbb{T}_3} \sum_{i=1}^{K} d\left(b, b_j^{t_i}\right)^2, \quad \text{(S5)}$$

and so $\overline{B} = \{\overline{b_1}, \dots, \overline{b_H}\}$. For notational simplicity, we will refer to $\overline{B}$ as the averaged backbone.

To quantify the extent to which our approximation disrupts the backbone structure, we consider the distortion of the pairwise distances between its atoms, taking inspiration from the Gromov-Hausdorff distance. We define the *distortion* of a function $f : X \to Y$ between two metric spaces as,

$$\operatorname{dis}(f) = \sup_{x,x'\in X} |d_X(x,x') - d_Y(f(x), f(x')|, \quad \text{(S6)}$$

which measures how the metric space is distorted when mapped into Y by f. The *codistortion* of $f : X \to Y$ and $g : Y \to X$ is given by,

$$\operatorname{codis}(f,g) = \sup_{x\in X, y\in Y} |d_X(x, g(y)) - d_Y(f(x), y|. \quad \text{(S7)}$$

Given two compact metric spaces X,Y, the joint information of distortion and codistortion can be used to define the Gromov-Hausdorff distance as,

$$d_{G-H}(X,Y) = \inf_{f,g} \max\{dis(f), dis(g), codis(f,g)\} \quad \text{(S8)}$$

with $f : X \to Y$ and $g : Y \to X$ being any functions.

Inspired by these definitions, we consider the correspondence between $\overline{B}$ and B_{t_i}, for every i given by the bijection $\varphi_i : \overline{B} \to B_{t_i}$, which simply associates the j-th atom position in the averaged backbone to the j-th atom position in the simulated data at time t_i,

$$\varphi_i(\overline{b_j}) = b_j^{t_i}, \quad \text{(S9)}$$

and compute the distribution of the pointwise distortion it induces, relative to the torus diameter. That is, we collect all the values,

$$\left| d(\overline{b}_j, \overline{b}_s) - d\left(b_j^{t_i}, b_s^{t_i}\right) \right| / \operatorname{diam}(\mathbb{T}_3), \quad \text{(S10)}$$

over different atoms (all couples j,s) and time-steps t_i, and then we take the associated density functions. We call it the *relative distortion distribution* (RDD). To quantify how much we distort our data by considering the Frechét mean, we report in Supplementary Table 2 the 90th percentile of the RDD. For instance, we find that, at low temperatures, across all families of glasses, 90% of pairwise distances are distorted by less than 2% of the box size, implying a high degree of similarly between the Frechét means and the original backbones. As expected, the reliability of our "fixed" approximation of the dynamics heavily depends on the mobility of the backbone. This is clearly reflected in Supplementary Table 2, i.e., as the temperature increases, the error increases. For the LiBP family, which has a connected and thus more rigid backbone, consistently low errors are observed even when the temperature increases.

3. Fixed representation of data: conducting ions

Next, we consider the second problem in our data pre-processing to obtain a static backbone approximation. That is, since at every time step, the ions' movement is determined by their position with respect to the backbone, if, by taking the Frechét mean, we change the position that the backbone's atoms have in the simulated data, we must change the ions position accordingly. We do so in the following fashion, as also illustrated in Supplementary Fig. 2.

For every backbone atom, at every time step, i.e., for every $b_j^{t_i}$, we indicate the translation vector which brings $b_j^{t_i}$ to $\overline{b}_j$ as,

$$v_{i,j} = \log_{b_j^{t_i}}(\overline{b_j}) \in \mathrm{Tan}_{b_j^{t_i}}(\mathbb{T}_3). \tag{S11}$$

Our aim is to move the conducting ions according to what happens in the nearby backbone atoms. Thus, for every conducting ion, we average the backbone movement vectors (transported in the tangent space at the chosen conducting ion), weighting them by a Gaussian kernel with the squared distance between the ion and the backbone atoms. So, first we define $\tilde{v}_{i,j}^{s}$ as the result of evaluating at the vector $v_{i,j}$ the parallel transport (along geodesics) operator,

$$\Gamma\left(b_j^{t_i}, l_s^{t_i}\right): \mathrm{Tan}_{b_j^{t_i}}(\mathbb{T}_3) \to \mathrm{Tan}_{l_s^{t_i}}(\mathbb{T}_3). \tag{S12}$$

That is, $\tilde{v}_{i,j}^{s}$ is the tangent vector $v_{i,j}$ transported, "seen", in the tangent space at $l_s^{t_i}$.

Then we take the weighted average of those vectors in $\mathrm{Tan}_{l_s^{t_i}}(\mathbb{T}_3)$. That is, we define,

$$V_{i,s} = \frac{1}{R_{i,s}} \sum_{j=1}^{N} e^{-d^2\left(b_j^{t_i}, l_s^{t_i}\right)} \cdot \tilde{v}_{i,j}^{s} \in \mathrm{Tan}_{l_s^{t_i}}(\mathbb{T}_3), \tag{S13}$$

with $R_{i,s} = \sum_{j=1}^{N} e^{-d^2\left(b_j^{t_i}, l_s^{t_i}\right)}$.

Lastly, we obtain the "pulled-back" ion $\overline{l}_s^{t_i}$ as,

$$\overline{l}_s^{t_i} := \exp_{l_s^{t_i}}(V_{i,s}). \tag{S14}$$

This time, to validate this approximating procedure, we consider how good it is in preserving the mean squared displacement. To this end, we compute it using the Frechét mean backbone $\overline{B}$ and pulled-back ions positions $\overline{l}_s^{t_i}$ and compare it with the original value. The results are also contained in Supplementary Table 2. The reason we need to approximate also the ions paths is that we will use them to validate a discretization scheme that is introduced in the following section.

4. Void space and its graph approximation

At this point, we have the following scenario: a fixed point cloud $\overline{B}$ given by the averaged backbone, and a set of conducting ions whose coordinates change with time and move in the void space $\mathbb{T}_3 - \overline{B}$. As conducting ions cannot get arbitrarily close to atoms in the back bone, they cannot navigate the whole space $\mathbb{T}_3 - \overline{B}$. Thus, we now obtain a better estimate of the space in which the ions can move. To do so, for each backbone atom

type, we compute common radius as the onset of the first peak of the radial distribution function from the number density $n(r)$ of the distances r between $b_j^{t_i}$ and $l_s^{t_i}$,

$$g(r) = \frac{n(r)}{4\pi r^2 \mathrm{d}r \rho_0} \tag{S15}$$

This represents the common minimal observed distance between the fixed backbone atom and any conducting ion, across all time steps t_i. Using these radii, we obtain an estimate of the region where ions are most likely to move, in fact we can inflate balls of radius r_j around every atom $\overline{b_j}$, obtaining:

$$\overline{B}^r \coloneqq \bigcup_{j=1,\dots,N} B_{\overline{b_j}}(r_j), \tag{S16}$$

where we recall that $B_{\overline{b_j}}(r_j) = \{p \in \mathbb{T}_3 | d(p, \overline{b_j}) < r_j\}$. Consequently, the void space then becomes the set,

$$X = \mathbb{T}_3 - \overline{B}^r. \tag{S17}$$

Next, we obtain a discretization of X, which can effectively be used for computations. The main idea is that we want to employ a regular grid $\{z_1, \dots, z_p\} \subset \mathbb{S}_1$, inducing a regular grid $G \subset \mathbb{T}_3$ made by points $z = (z_i, z_j, z_k)$ and use it to approximate X. However, having a grid approximation of X is not enough for our purposes, as we want to extract from X some topological information. That is, having just a discrete set is not helpful. Instead, we want to build some graph structure out of G, which can contain some proximity information regarding its points (as in Figure 2c). The easiest and most natural way to do so is to connect every point inside G to all its points in a cubic neighborhood. In particular, a general point $z = (z_i, z_j, z_k)$ (avoiding for notational simplicity the case i or j or k being equal to 1 or P) will have edges towards all the points in G of the form $p = (p_1, p_2, p_3)$, such that: $p_1 \in \{z_{i-1}, z_i, z_{i+1}\}$, $p_2 \in \{z_{j-1}, z_j, z_{j+1}\}$, $p_3 \in \{z_{k-1}, z_k, z_{k+1}\}$, and $p \neq z$. The key point is now that it is easy to check the condition $d(z, \overline{b_j}) \leq r_j$, i.e., we can easily obtain a graph X_G whose vertex set is $X \cap G$ and whose edges connect the vertices that are connected by edges in G. This is shown in Figures 2c and 2d.

As the last point of this section, we highlight that in our procedure, we need to fix P, that is, the size of the grid, a very important hyperparameter. We do so in two ways: (1) by trying different values for P to compute our max-flow and consider the stability of the computed value; and (2) by checking how the approximated ion paths change when projected onto X_G. We ended up using a grid spacing of approximately 0.2 Å to calculate the needed P for all computed systems.

Supplementary Table 3 contains the results of the additional test we do to assess the goodness of our grids. The idea of this test is simple: since we have recovered the paths of the ions around the averaged backbone, we can use them to confirm if the employed grid is fine enough to approximate the given paths reasonably. In particular, for each sample of glass at temperature 300 K, we proceed as follows:

1. we randomly sample an ion $\overline{l}_s^{t_i}$ and consider its next position $\overline{l}_s^{t_{i+1}}$ and consider the distance between the two in the torus $\mathbb{T}_3$;
2. then we project $\overline{l}_s^{t_i}$ onto its closest point in X_G, and similarly for $\overline{l}_s^{t_{i+1}}$. Then, we compute the shortest path distance between those two grid points;

3. we compute the absolute difference between the two distances that we have measured;
4. to relate this quantity to the different box sizes and with the size of the grid, we express this difference as the integer ratio over $\delta = d_{\mathbb{S}^1}(z_{k-1}, z_k)$, obtaining the *path approximation ratio* (PAR). Any value between 0 and 2 can be related to the grid approximation errors;
5. we repeat this sampling procedure 10,000 times for each glass sample and then aggregate the results by glass family and consider the associated histograms.

Before commenting on the results of such procedure we point out that we compute the length of the geodesic (i.e. the distance) between $\bar{l}_s^{t_i}$ and $\bar{l}_s^{t_{i+1}}$ in the torus and not in X because the geodesic in X is not available in closed form and so we would have to do yet another approximation. However, since we consider low temperatures (and so the backbone's movements are less relevant) the path between $\bar{l}_s^{t_i}$ and $\bar{l}_s^{t_{i+1}}$ is a reliable approximation of the path between $l_s^{t_i}$ and $l_s^{t_{i+1}}$ and since we consider small time steps $t_i - t_{i-1}$, a geodesic in the torus is a good approximation of the path between $\bar{l}_s^{t_i}$ and $\bar{l}_s^{t_{i+1}}$. Moreover, for higher temperatures (700-800 K) the minimal observed radii are smaller and so the void spaces are bigger, and thus if the grid resolution is enough to obtain a good approximation of the void space at 300 K, then it is good also for higher temperatures.

In Supplementary Fig. 3 we represent different possible situations that can be obtained when repeating the procedure just outlined: a) the grid approximation either faithfully represents the original path, b) a path between the atom's locations projected on the grid does not exist, c) such path is much longer than the original path due to the grid not being fine enough. In Supplementary Table 2 we report the percentage of sampled paths whose PAR is less or equal than 2. We see that most of the paths are faithfully recovered by our grid approximation.

5. Mapper graphs

The next step in our pipeline is contextually (1) a dimensionality reduction step, to avoid having to deal with $\sim 10^7$ grid points; and (2) a required step to fit into the theoretical framework developed in [2]. The reader may refer to Figures 2e and Supplementary Fig. 5 for a visual representation of the following section. We subdivide the procedure to obtain a graph simplification of X_G, called the *weighted mapper graph*, in the following points:

1. arbitrarily fix a coordinate 1 or 2 or 3 for the points $p = (p_1, p_2, p_3) \in X_G$. Without loss of generality, suppose that we fixed the last coordinate. We point out that, in our pipeline, we repeat this procedure for every possible choice of coordinate;
2. fix an interval of the form $[z_k, z_{k+1}]$ or $[z_P, z_1]$;
3. consider the subgraph X_G^k containing all points $p = (p_1, p_2, p_3) \in X_G$ such that $p_3 = z_k$ or z_{k+1} and the edges between them;
4. for every X_G^k, let $\pi_0(X_G^k) = \{V_1^k, \dots, V_{n_k}^k\}$ be the set of its path connected components. With this information, considering all the different intervals (i.e., varying k), we start building $\mathcal{M}$ the mapper graph

of X_G, taking as vertex set $\coprod_{k=1,\dots} \pi_0(X_G^k)$. That is, every path connected component, in every sub-graph X_G^k, becomes a vertex of $\mathcal{M}$;

5. we need to define the edges of $\mathcal{M}$. We want these edges to encode pairwise information between the path connected components represented by the vertices. To this end, note that each z_k belongs to exactly two of the intervals we have considered: $[z_k, z_{k+1}]$ and $[z_{k-1}, z_k]$. Thus, a point of the form $(p_1, p_2, z_k) \in X_G$ belongs both to X_G^{k-1} and to X_G^k. That is, it belongs to one path connected component in X_G^{k-1} and one in X_G^k. We trace down exactly this information: given $V_i^{k-1} \in X_G^{k-1}$ and $V_i^k \in X_G^k$, we add the edge (V_i^{k-1}, V_j^k) if $V_i^{k-1} \cap V_j^k \neq \emptyset$. Note that we want to consider edges also with a direction, that is, a tip and a tail. Thus, we refer to (V_i^{k-1}, V_j^k) as directed edges, or arcs, and we imply that V_j^k is the tip and V_i^{k-1} is the tail. Note how the shape of the mapper graph in Figure 2e is the same as the paths represented by the thick red arrows in the left panel of the same figure;
6. as the last step we compute some weights that we attach to the edges of $\mathcal{M}$. As we have just seen, edges represent intersections between path connected components of sub-graphs, which we can interpret as “local sockets” in X_G. In other words, each directed edge can be seen as a “tunnel” connecting two of these sockets (see Supplementary Fig. 3 for a visual representation). Therefore, the information that we want to extract from X_G and attach to these tunnels is their size: to each edge (V_i^{k-1}, V_j^k), we attach the cardinality of $V_i^{k-1} \cap V_j^k$ (which, by construction, is non-empty). If we write the mapper graph as $\mathcal{M} = (V, E)$, V being its vertices and E its directed edges, this last step amounts to defining a function $C : E \to \mathbb{R}_{\geq 0}$, which is the function associating to each edge its maximum capacity, which we will use for the max-flow.

Thus, we have constructed the weighted mapper graph $\mathcal{M}$, as in Figure 2e and Supplementary Fig. 4. To be able to use the *circular flow* defined in ref. [2], we need to add one last piece of structure to $\mathcal{M}$, which is given almost for free by our construction. This amounts to another graph $\mathcal{S}$ and a function f between $\mathcal{M}$ and $\mathcal{S}$. We build $\mathcal{S}$, which will be a discretization of the circle $\mathbb{S}^1$, as follows:

1. we consider one vertex v_k for each interval $[z_k, z_{k+1}]$ or $[z_P, z_1]$; and
2. we consider directed edges of the form (v_k, v_{k+1}) or (v_P, v_1).

In this way, we obtain the inner circular graph in Supplementary Fig. 5. Here, the function $f : \mathcal{M} \to \mathcal{S}$ is represented by vertices of the corresponding colors. With our notation, it can be easily written as $f(V_j^k) = v_k$.

6. Circular max-flow

Now we briefly report the definition of circular max-flow given in ref. [2] that we use in our data analysis pipeline.

Definition 1. Given $\mathcal{M} = (V, E)$, we define the directed chains as the vector spaces:

$$\mathrm{DCh}_0(\mathcal{M}, \mathbb{R}) := \{s = \textstyle\sum_{v \in V} \lambda_v \cdot v \mid \lambda_v \in \mathbb{R}\}, \quad \text{(S18)}$$

$$\mathrm{DCh}_1(\mathcal{M}, \mathbb{R}) := \{s = \textstyle\sum_{e \in E} \lambda_e \cdot e \mid \lambda_e \in \mathbb{R}\}. \quad \text{(S19)}$$

Then, we define $\partial_1 : \mathrm{DCh}_1(\mathcal{M}, \mathbb{R}) \to \mathrm{DCh}_0(\mathcal{M}, \mathbb{R})$ as the linear extension of $\partial_1\big((v, w)\big) = w - v$. In particular, for the directed chain $\partial_1(s)$, the coefficient associated to vertex v is,

$$\lambda_v = \sum_{(w,v)\in \mathrm{In}(v)} \lambda_{(w,v)} - \sum_{(w,v)\in \mathrm{Out}(v)} \lambda_{(w,v)}, \tag{S20}$$

where In(v) and Out(v) are the edges going into v and out of v, respectively. We call $\mathrm{F}_1(\mathcal{M}, \mathcal{R}) = \ker(\partial_1)$. Restricting to chains with non-negative coefficients we obtain,

$$\mathrm{F}_1^+(\mathcal{M}, \mathcal{R}) = \{s \in \mathrm{F}_1(\mathcal{M}, \mathcal{R}) | \lambda_e \geq 0, \forall e \in E\}. \tag{S21}$$

Elements in $\mathrm{F}_1^+(\mathcal{M}, \mathcal{R})$ are called circulations coherently with the max-flow literature [3].

Clearly, we can define circulations also for the graph $\mathcal{S}$ and in ref. [2] it is proven that for $\mathcal{S}$, only constant circulations are allowed (see also Supplementary Fig. 5). That is, $\mathrm{F}_1(\mathcal{M}, \mathcal{R}) \cong \mathcal{R}$ and so $\mathrm{F}_1^+(\mathcal{M}, \mathcal{R}) \cong \mathbb{R}_{\geq 0}$.

The function $f : \mathcal{M} \to \mathcal{S}$ induces a linear map sending a circulation on $\mathcal{M}$ onto a circulation on $\mathcal{S}$ as described visually in Supplementary Fig. 5. This map can be written as,

$$\mathrm{F}_1^+(f) : \mathrm{F}_1^+(K, \mathbb{R}) \to \mathrm{F}_1^+(Q, \mathbb{R}) \cong \mathbb{R}_{\geq 0}. \tag{S21}$$

The last piece of the equation we need to take into account is the set of maximal capacities $C : E \to \mathbb{R}_{\geq 0}$ that we have already defined. It quantifies how many ions can flow through a particular arc.

We indicate with $s \leq C$ the set $s \in \mathrm{F}_1^+(\mathcal{M}, \mathbb{R})$ such that for each arc $e \in E$: $\lambda_e \leq C(e)$. Then, we finally define the circular max-flow of $f : \mathcal{M} \to \mathcal{S}$, with maximal capacities C as,

$$\mathbf{F}(f, C) = \max_{s \leq C} \mathrm{F}_1^+(f). \tag{S22}$$

Supplementary Text

General computational complexity of the max-flow method

Under the standing assumption that the backbone point cloud and the radii are given as input, the dominant computational cost of the geometric/topological part of the max-flow pipeline arises from discretizing the periodic void space on a $P \times P \times P$ grid and testing membership against the thickened backbone; see the description of the void-space graph approximation and mapper construction in the manuscript and Supplementary Information. Let $\mathcal{B}$ denote the set of backbone atoms, and let

$$\#G := P^3 \tag{S23}$$

be the total number of grid points, consistently with the notation of the Supplementary Methods.

A direct implementation of the void-space test computes, for each grid point, its toroidal distance to all backbone atoms and checks whether the point lies outside the chosen thickening radius. In the absence of further spatial acceleration, this step has complexity

$$O(\#G\ \#\mathcal{B}) = O(P^3\ \#\mathcal{B}). \tag{S24}$$

Once the retained grid points have been identified, let $N \leq \#G$ denote their number. The construction of the retained grid graph is then linear in N, since each retained point is connected only to retained points in its local neighborhood, so the number of grid-graph edges is $O(N)$ up to a fixed constant factor. Moreover, connected components of an undirected graph can be computed in time $O(\#V + \#E)$ by repeated breadth-first or depth-first search; see, e.g. Refs. [4,5]. Hence each connected-components computation appearing in the mapper construction is linear in the size of the corresponding subgraph.

The subsequent construction of the mapper graph is likewise linear in the size of the retained graph: the cover along one coordinate has constant overlap, each retained point belongs to at most two cover elements, and connected components are computed on subgraphs of the retained grid graph. Thus, both the computation of the mapper graph and the assignment of its edge capacities are of order

$$O(N). \tag{S25}$$

The same holds for the assignment of edge capacities, since capacities are computed from overlap cardinalities between adjacent cover elements and therefore require only a linear pass through the relevant retained-grid incidences. Hence the full compression stage, from the retained grid graph to the weighted mapper graph, has complexity

$$O(N). \tag{S26}$$

This step is also the key dimensionality-reduction stage of the pipeline, as it replaces a graph with on the order of 10^7 grid points by a much smaller weighted graph that still records the channel and slice connectivity relevant for the circular max-flow.

The final optimization step is not performed on the full retained grid graph, but on the much smaller weighted mapper graph. If $M = (V, E)$ denotes the weighted mapper graph, then the circular max-flow problem is a linear program whose size is controlled by $\#V$ and $\#E$, and therefore has complexity polynomial in the mapper-graph size:

$$\mathrm{poly}(\#V, \#E). \tag{S27}$$

Accordingly, the overall complexity of the geometric/topological stage may be summarized as

$$O(P^3\,\#\mathcal{B}) + O(N) + \mathrm{poly}(\#V, \#E), \tag{S28}$$

In all cases, the grid-based void-space discretization typically represents the dominant bottleneck, while the final optimization is carried out only on the compressed mapper graph.

Practical note

We note that the current implementation was primarily designed to validate the methodology and its predictive power, rather than to optimize each individual step to its best possible asymptotic or practical computational cost. In particular, the current choice of P is made empirically through stability and path-recovery tests, and some stages, most notably the mapper graph construction, could likely be improved substantially by more specialized data structures or other implementation-level optimizations.

A toy complexity model from the atomic configuration

In the following, we outline a toy complexity model, relating the cost of the geometric/topological part of the pipeline to basic geometric features of the underlying periodic atomic configuration. The goal is not to prove a rigorous worst-case theorem, but rather to provide a simple scaling picture explaining why the mapper construction can drastically reduce the size of the graph on which the final max-flow problem is solved. Throughout, the notation

$$A \asymp B \tag{S29}$$

means that A and B are comparable up to multiplicative constants, while $O(\cdot)$ denotes the usual asymptotic upper bound up to a constant factor.

<u>Geometric setting:</u> Following the Supplementary Methods, we start from a fixed-point cloud given by the averaged backbone in the simulation box with periodic boundary conditions, viewed as a flat 3-torus. Let $\mathcal{B}$ denote the set of backbone atoms. For each backbone atom $\bar{b}_j$, one defines a radius r_j from the minimal observed distance to the diffusing ions across time, and then considers the resulting void space

$$X := \mathbb{T}^3 \setminus \bigcup_j \overline{B(\bar{b}_j, r_j)}. \tag{S30}$$

This is the region in which the conducting ions are assumed to move. The void space is then discretized by a regular grid of size P in each coordinate, yielding a grid G and its graph approximation X_G. The corresponding weighted mapper graph is denoted by

$$M = (V, E), \tag{S31}$$

with capacity function

$$C: E \to \mathbb{N}. \tag{S32}$$

This matches the notation introduced in Supplementary Methods sections 4-6.

Choice of the grid scale: A natural quantity controlling the required resolution is the size of the smallest relevant void feature that should still be captured by the grid. We denote this heuristic scale by

$$w_{\min} > 0, \tag{S33}$$

and interpret it as the width of the narrowest transport-relevant channel or neck in the void space. A toy resolution criterion is then to choose the grid step δ so that

$$\delta \le \alpha\, w_{\min}, \qquad \alpha \in (0,1). \tag{S34}$$

Equivalently, if the side lengths of the simulation box are of order L, then one expects

$$P \asymp \frac{L}{\delta} \gtrsim \frac{L}{\alpha w_{\min}}. \tag{S35}$$

For simplicity, we assume here that all side lengths are comparable and write L^3 as a proxy for the box volume. We emphasize again that this is not the grid-selection rule used in the present paper: here P is chosen empirically through numerical stability and path-recovery diagnostics. Rather, the present discussion should be read as a possible direction for replacing that empirical choice by an a priori geometric criterion in future work.

Size of the retained graph X_G: Let

$$\phi := \frac{\mathrm{Vol}(X)}{\mathrm{Vol}(\mathbb{T}^3)} \in (0,1) \tag{S36}$$

denote the void fraction. Since the full grid contains

$$\#G = P^3 \tag{S37}$$

points, the number of retained grid points in X_G is heuristically of order

$$\#X_G \asymp \phi P^3. \tag{S38}$$

Using the above choice of resolution, this becomes

$$\#X_G \asymp \phi\left(\frac{L}{\alpha w_{\min}}\right)^3. \tag{S39}$$

Likewise, the number of edges of X_G is linear in the number of its vertices, so the retained graph remains of cubic order in the inverse grid scale.

A topological heuristic for the size of the mapper graph: We now estimate the size of the mapper graph $\mathrm{M} = (\mathrm{V}, \mathrm{E})$. In Supplementary Methods Section 5, the mapper construction proceeds by fixing one coordinate direction, considering overlapping intervals made of consecutive grid levels, building the subgraphs $\mathrm{X}_{\mathrm{G}}^{\mathrm{k}}$, and then taking their path connected components as vertices of the mapper graph. Edges record non-empty intersections of such components in adjacent overlaps, and capacities are given by the cardinalities of these intersections.

Let C_k denote the number of path connected components of the subgraph X_G^k. Then, at the level of scaling,

$$\#V \asymp \textstyle\sum_k C_k, \tag{S40}$$

while $\#E$ is of the same order as the total number of non-empty overlap incidences between components in consecutive slices.

To obtain a heuristic control on C_k, let O_k denote the union of the inflated backbone balls intersected with the k-th thin slice, and let V_k denote the corresponding void region in that slice. Then C_k is the discrete counterpart of $\beta_0(V_k)$, where β_0 is the 0-th Betti number, that is, the number of connected components. Likewise, $\beta_1(O_k)$ is the 1-st Betti number, which qualitatively counts the number of independent loops or "holes" in the sliced obstacle set.

In an effectively two-dimensional thin-slice regime, Alexander duality gives, for a sufficiently regular compact set $A \subset \mathbb{R}^2$,

$$\beta_0(\mathbb{R}^2 \backslash A) = \beta_1(A) + 1; \tag{S41}$$

see, for instance Ref. [6]. A visual interpretation is given in Supplementary Figure 6a. This says that, in two dimensions, the number of connected components of the complement is exactly controlled by the number of independent loops in the obstacle set. In our setting each slice is periodic, so strictly speaking it is closer to a thin 2-torus than to the plane or the sphere. However, for a very thin slice the same picture remains a reasonable guiding principle: the connectivity of the void region is still expected to be governed by the loop complexity of the sliced obstacle set, up to global corrections coming from periodicity. This motivates the heuristic estimate

$$C_k \lesssim 1 + \beta_1(O_k). \tag{S42}$$

In other words, the slice complexity is governed not merely by the number of nearby atoms, but more fundamentally by the topological complexity of the excluded region inside the slice.

<u>From topological complexity to atom count:</u> The topological heuristic above can be combined with a crude local-complexity assumption. Let N_k denote the number of backbone atoms whose inflated balls intersect the k-th slice. In the effectively two-dimensional thin-slice regime, it is reasonable to assume that each additional intersecting atom creates at most $O(1)$ new independent loops in the sliced obstacle set, so that

$$\beta_1(O_k) = O(N_k). \tag{S43}$$

This assumption is tied to the fact that the slice is modeled as essentially two-dimensional. A simple grid cartoon illustrating this phenomenon is shown in Supplementary Figure 6b: adding one local obstacle can create only a bounded number of new elementary loops. Consequently,

$$C_k = O(1 + N_k). \tag{S44}$$

Hence atom count acts only as a coarse proxy for the homological complexity of the sliced obstacle set. If the atoms are distributed with average density,

$$\rho_a := \frac{\#\mathcal{B}}{L^3} \tag{S45}$$

and the slice thickness is of order δ then one expects

$$N_k \asymp \rho_a L^2 \delta \tag{S46}$$

and therefore:

$$C_k = O(1 + \rho_a L^2 \delta). \tag{S47}$$

Heuristic size of the mapper graph: Summing over the $P \asymp L/\delta$ slices gives

$$\#V = O(P(1 + \rho_a L^2 \delta)). \tag{S48}$$

Using $P \asymp L/\delta$ and $\rho_a = \#\mathcal{B}/L^3$, we obtain

$$P(1 + \rho_a L^2 \delta) \asymp \frac{L}{\delta}\left(1 + \frac{\#\mathcal{B}}{L^3} L^2 \delta\right) = \frac{L}{\delta} + \frac{\#\mathcal{B}}{L}. \tag{S49}$$

Since L is treated here as part of the ambient geometric scale, this yields the simplified estimate

$$\#V = O\left(\frac{L}{\delta} + \#\mathcal{B}\right), \tag{S50}$$

and similarly

$$\#E = O\left(\frac{L}{\delta} + \#\mathcal{B}\right). \tag{S51}$$

Under the resolution choice

$$\delta \asymp \alpha w_{\min}, \tag{S52}$$

this becomes

$$\#V, \#E = O\left(\frac{L}{\alpha w_{\min}} + \#\mathcal{B}\right). \tag{S53}$$

Compression ratio: Within this toy model, the retained graph has size

$$\#X_G \asymp \phi\left(\frac{L}{\alpha w_{\min}}\right)^3, \tag{S54}$$

whereas the mapper graph has size

$$\#V, \#E = O\left(\frac{L}{\alpha w_{\min}} + \#\mathcal{B}\right). \tag{S55}$$

Therefore, the ratio

$$\frac{\#X_G}{\#V} \tag{S56}$$

can be interpreted heuristically as the average number of retained grid vertices that are compressed into one mapper vertex. When the term $\#\mathcal{B}$ is of lower order than $L/(\alpha w_{\min})$, this ratio is of order

$$\frac{\#X_G}{\#V} \asymp \phi\left(\frac{L}{\alpha w_{\min}}\right)^2. \tag{S57}$$

Thus, the retained graph reflects a three-dimensional discretization of the void space and scales cubically in the inverse grid spacing, while the mapper graph may scale much more mildly, essentially like the number of slices corrected by a term depending on the local obstacle complexity.

Implications for the overall complexity: Under the present input model, the most expensive stage in a direct implementation is typically the construction of the discretized void space. Indeed, if one tests membership of each grid point against all backbone atoms, this step has cost

$$O(\#\mathcal{B}\, P^3) = O\left(\#\mathcal{B}\left(\frac{L}{\alpha w_{\min}}\right)^3\right). \tag{S58}$$

Once X_G has been built, the graph construction and the connected-components computations involved in the mapper step are linear in the size of the relevant subgraphs. The final circular max-flow is then solved on the

weighted mapper graph $M = (V, E)$, , and can be treated algorithmically as a classical min-cost circulation problem on a constant-size augmentation of that graph. Hence the toy end-to-end complexity model becomes

$$O\left(\#\mathcal{B}(\frac{L}{\alpha w_{\min}})^3\right) + O\left((\frac{L}{\alpha w_{\min}} + \#\mathcal{B})\right)^3 \ + \ \text{poly}\left(\frac{L}{\alpha w_{\min}} + \#\mathcal{B}\right). \tag{S59}$$

where the second term is obtained by inserting the estimate $\#\mathrm{V}, \#\mathrm{E} = O(\frac{L}{\alpha w_{\min}} + \#\mathcal{B})\#$ into the reference min-cost-flow complexity $O(\#V^2 \#E \ log \#V)$.

Thus, at the level of this toy model, the essential conclusion is that the optimization is performed only on the compressed mapper graph, and its cost is polynomial in the effective graph size. In other words, after compression to the mapper graph, the optimization is expected to scale polynomially in the effective one-dimensional slice complexity, rather than in the full three-dimensional grid size.

Interpretation and limitations: The purpose of this toy model is not numerical accuracy, but rather to explain the different scaling regimes of the two graph constructions. The graph X_G represents a three-dimensional discretization of the void space and therefore has a size that is cubic in the inverse grid scale. By contrast, the mapper graph records how path connected components evolve across overlapping slices and may therefore be much smaller. At the same time, this toy model should not be interpreted as a direct predictor of CPU time. In the actual implementation, the circular max-flow is solved through a linear-programming formulation on the compressed mapper graph, so the practical runtime depends not only on the asymptotic graph size but also on solver-level effects such as sparsity, conditioning, and implementation details. As a consequence, two graphs of comparable theoretical size may still have quite different actual solution times. For this reason, the present complexity discussion should be read as a conservative structural model rather than as a quantitative runtime law. Finally, this discussion concerns only the geometric/topological stage of the pipeline. The chemical problem of predicting the radii remains outside the scope of the present complexity analysis and is better viewed as a separate local learning or regression task. Likewise, the present paper does not yet resolve the principled choice of the grid size, the identification of quantities such as $w_{\min}$, or a more theoretical treatment of system-size effects in the simulated materials.

Supplementary Tables

Supplementary Table 1. Overview of the tested glass compositions in the present work, including their MD-simulated ionic conductivity (σ), slice area, normalized max-flow, and MD force field used for its generation.

Composition	log(σ) at 300 K (log(S m^{-1}))	Slice Area (Å^2)	Normalized Max-flow (Å^{-2})	Force field
$Li_2Si_4O_9$	-3.41	1171.5	0.247	MLFF
$Li_2Si_2O_5$	-3.03	1091.3	0.217	MLFF
Li_2SiO_3	-2.78	991.7	0.160	MLFF
$Na_2Si_4O_9$	-3.30	1225.4	0.054	MLFF
$Na_2Si_2O_5$	-2.93	1192.1	0.059	MLFF
Na_2SiO_3	-3.96	1156.7	0.049	MLFF
$Li_2Si_4S_9$	-2.10	1752.6	0.138	MLFF
$Li_2Si_2S_5$	-1.33	1632.0	0.091	MLFF
Li_2SiS_3	-1.14	1502.2	0.086	MLFF
$Na_2Si_4S_9$	-2.04	1803.0	0.021	MLFF
$Na_2Si_2S_5$	-2.17	1740.4	0.030	MLFF
Na_2SiS_3	-2.45	1673.0	0.025	MLFF
$Li_2P_4O_{11}$	-3.61	1240.2	0.287	MLFF
$Li_2P_2O_6$	-3.00	1136.8	0.245	MLFF
$Li_4P_2O_7$	-2.84	1030.4	0.176	MLFF
$Na_2P_4O_{11}$	-3.99	1255.4	0.066	MLFF
$Na_2P_2O_6$	-4.05	1202.8	0.031	MLFF
$Na_4P_2O_7$	-3.62	1170.7	0.043	MLFF
$Li_2P_4S_{11}$	-0.94	1752.0	0.127	MLFF
$Li_2P_2S_6$	-0.96	1630.1	0.084	MLFF
$Li_4P_2S_7$	-1.13	1498.3	0.077	MLFF
$Na_2P_4S_{11}$	-1.95	1786.9	0.025	MLFF
$Na_2P_2S_6$	-2.43	1703.5	0.025	MLFF
$Na_4P_2S_7$	-2.44	1654.1	0.018	MLFF
$Li_2B_4O_7$	-3.30	973.2	0.122	MLFF
$Li_2B_2O_4$	-2.70	925.1	0.144	MLFF
$Li_4B_2O_5$	-2.88	901.5	0.133	MLFF
$Na_2B_4O_7$	-3.25	1062.8	0.046	MLFF
$Na_2B_2O_4$	-3.03	1079.3	0.047	MLFF
$Na_4B_2O_5$	-3.45	1120.5	0.059	MLFF
$Li_2B_4S_7$	-0.72	1461.7	0.062	MLFF
$Li_2B_2S_4$	-0.96	1385.3	0.060	MLFF
$Li_4B_2S_5$	-1.12	1353.7	0.057	MLFF
$Na_2B_4S_7$	-2.11	1529.0	0.013	MLFF
$Na_2B_2S_4$	-2.35	1534.6	0.018	MLFF
$Na_4B_2S_5$	-2.66	1563.7	0.019	MLFF
$Li_2Ge_4O_9$	-4.34	1194.0	0.171	MLFF
$Li_2Ge_2O_5$	-3.54	1120.9	0.153	MLFF
Li_2GeO_3	-2.84	1051.7	0.178	MLFF
$Na_2Ge_4O_9$	-3.84	1252.4	0.016	MLFF
$Na_2Ge_2O_5$	-3.78	1235.5	0.035	MLFF
Na_2GeO_3	-3.63	1206.4	0.040	MLFF

$Li_2Ge_4S_9$	-2.50	1715.9	0.023	MLFF
$Li_2Ge_2S_5$	-2.13	1629.6	0.044	MLFF
Li_2GeS_3	-1.53	1521.0	0.063	MLFF
$Na_2Ge_4S_9$	-2.99	1787.4	0.005	MLFF
$Na_2Ge_2S_5$	-3.25	1744.8	0.007	MLFF
Na_2GeS_3	-2.95	1701.6	0.021	MLFF
$38Li_2O-31B_2O_3-31P_2O_5$	-2.50	2310.6	0.172	Classical
$40Li_2O-30B_2O_3-30P_2O_5$	-2.53	2275.7	0.169	Classical
$42.5Li_2O-28.75B_2O_3-28.75P_2O_5$	-2.33	2246.7	0.176	Classical
$45Li_2O-27.5B_2O_3-27.5P_2O_5$	-2.27	2217.8	0.181	Classical
$67Li_2S-33P_2S_5$	0.20	3270.2	0.265	Classical
$70Li_2S-30P_2S_5$	0.47	3083.5	0.389	Classical
$75Li_2S-25P_2S_5$	-1.79	3026.8	0.395	Classical
$Na_2S-2SiS_2$	-1.79	3282.8	0.05	Classical
Na_2S-SiS_2	-0.54	3275.8	0.135	Classical
$2Na_2S-SiS_2$	0.08	3270.2	0.265	Classical

Supplementary Table 2. Experimental and simulated ionic conductivities of the investigated systems. Experimental values are sourced from the literature [7,8]. Simulated conductivities are derived from extrapolated room-temperature diffusivities.

Sample	Experimental ionic conductivity (S m^{-1})	Simulated ionic conductivity (S m^{-1})
67LiPS	$5.6\cdot10^{-3}$	$1.57\cdot10^{-0}$
70LiPS	$1.4\cdot10^{-2}$	$2.97\cdot10^{-0}$
75LiPS	$3.0\cdot10^{-2}$	$7.59\cdot10^{-0}$
33NaSiS	-	$1.61\cdot10^{-2}$
50NaSiS	-	$2.87\cdot10^{-1}$
67NaSiS	-	$1.20\cdot10^{-0}$
38LiBP	$1.4\cdot10^{-6}$	$3.19\cdot10^{-3}$
40LiBP	$2.8\cdot10^{-6}$	$2.98\cdot10^{-3}$
42.5LiBP	-	$4.68\cdot10^{-3}$
45LiBP	$1.6\cdot10^{-5}$	$5.43\cdot10^{-3}$

Supplementary Table 3. Metrics used to evaluate our approximation and discretization. In particular, for each glass family and temperature, we report the 90th percentile of the relative distortion distribution (RDD) and the MDS relative error. For the temperature of 300 K, we report the percentage of paths whose path approximation ratio (PAR) is lower than or equal to 2. The threshold of 2 is chosen because any PAR value below 2 can potentially arise as an artifact of computing the distances on a grid, regardless of its resolution.

Glass family	Temperature (K)	90th Perc. of RDD	MSD Relative Error	PAR $\leq$ 2
LiPS	300	0.017	0.002	0.931
	700	0.195	0.008	-
LiBP	300	0.006	0.037	0.992
	800	0.017	0.001	-
NaSiS	300	0.011	0.009	0.966
	800	0.135	0.010	-

Supplementary Figures

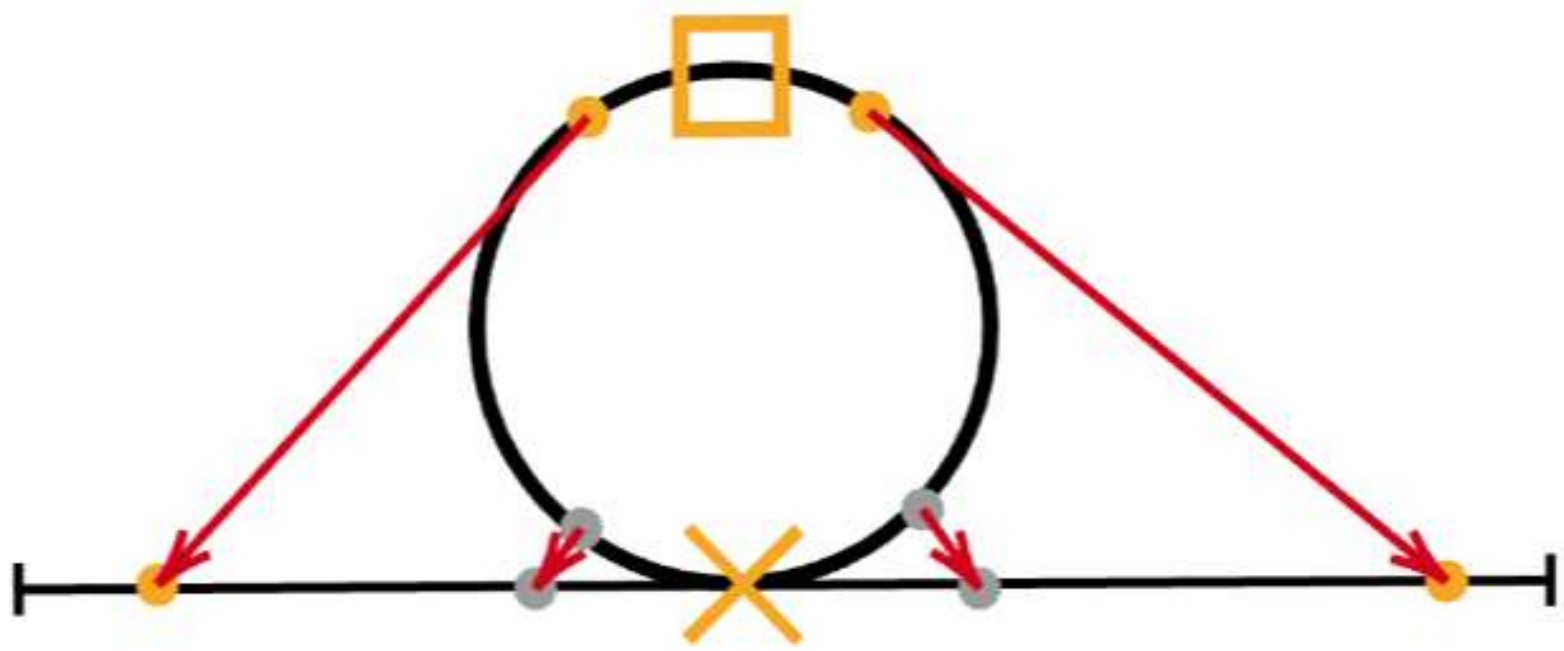

Supplementary Figure 1. Two data sets on $\mathbb{S}_1$ and their respective Frechét means and averaged coordinates. The first data set is made by the two grey points on $\mathbb{S}_1$. The same data set considered without the boundary condition is represented by the two grey points on the horizontal segment. Similarly, we consider a second data set made by the two orange points, both on $\mathbb{S}_1$ and "unrolled" onto a segment, without the periodic boundaries. The big orange X represents the mean of the coordinates of the points, for both data sets, which is a good notion of average for the grey data but a very bad notion of average for the orange points on $\mathbb{S}_1$. Instead, the orange square represents the Frechét mean of the orange points on $\mathbb{S}_1$. For all the other couples of points, the Frechét mean coincides with the average of the coordinates.

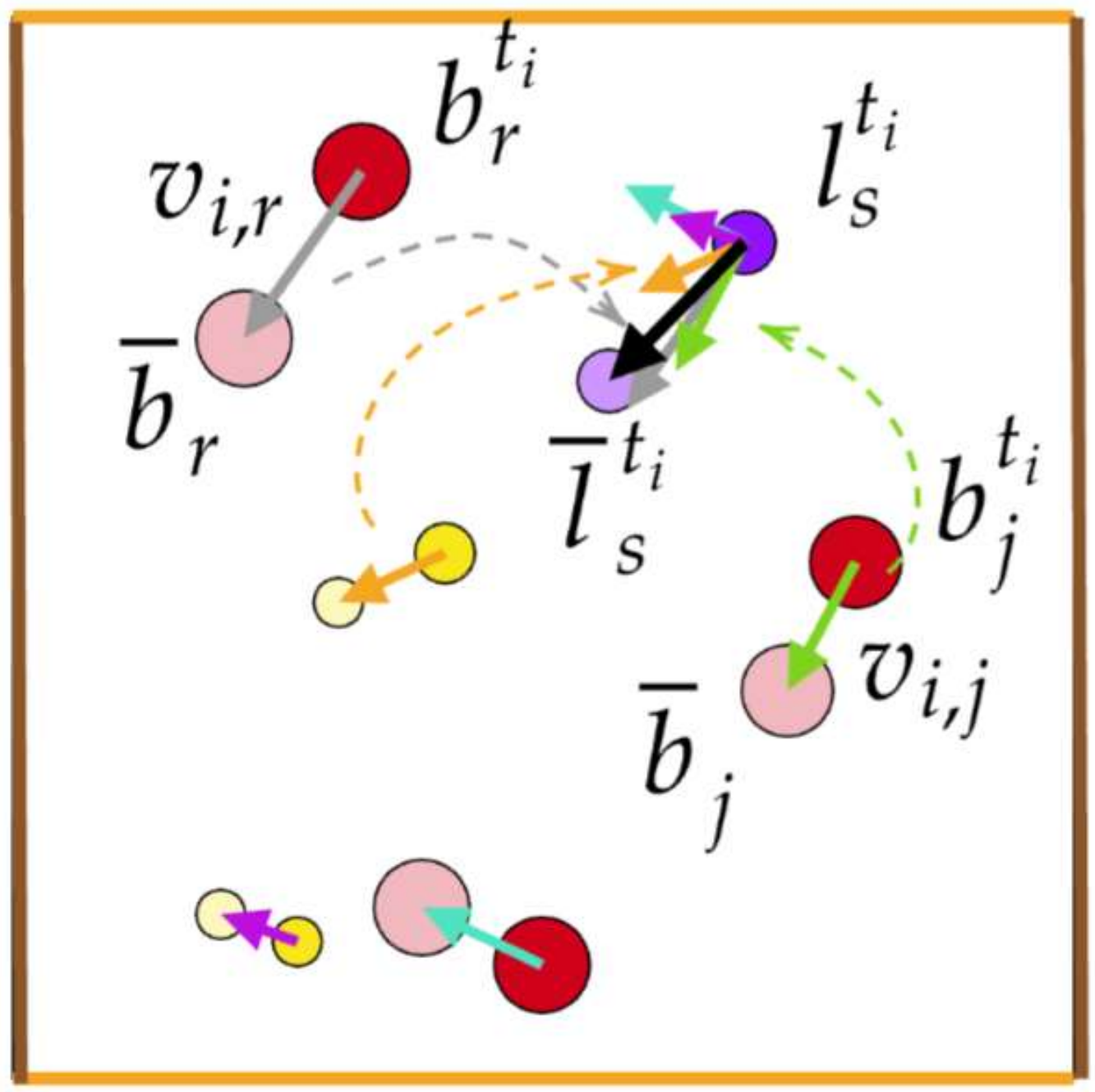

Supplementary Figure 2. A visual representation of the procedure described in Supplementary Methods section 3 to pull back ion movements, representing them relatively to the position of the averaged backbone. The various quantities shown in the figure are defined in the text. Ions are colored in shades of purples, while shades of yellow and shades of red indicate backbone atoms. The arrows centered at backbone's atoms indicate translations vectors and the corresponding arrows of the same color are the result of transporting along geodesics such vectors to an ion location. The weighted average of the transported vectors is drawn in black.

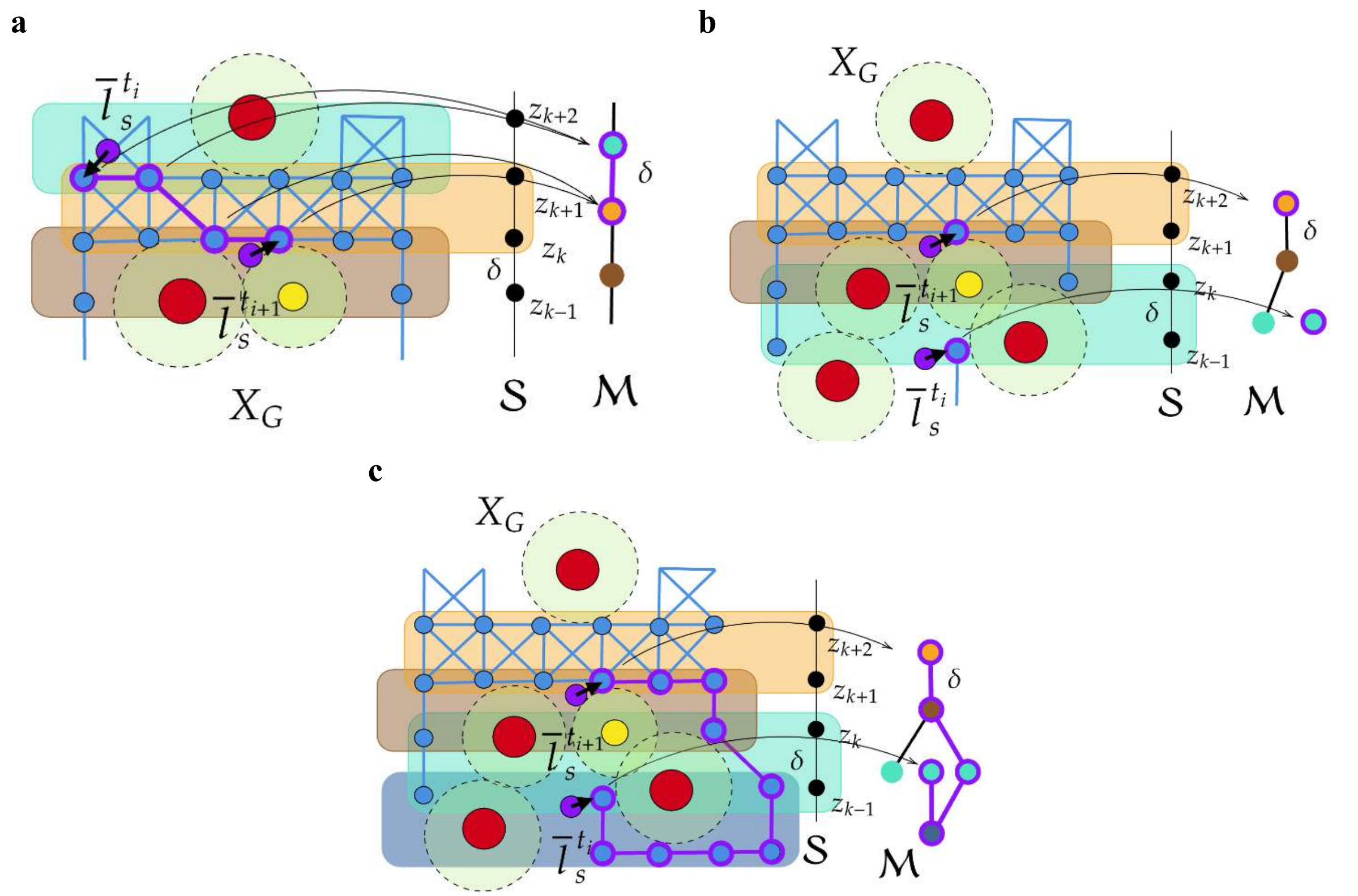

Supplementary Figure 3. Plots related to the tests supporting our choice for the grid cardinalities. We find different possible situations when approximating paths of conducting ions with the chosen grid. The original positions of the atoms are indicated by the purple points. Their projection onto the grid, via closed point projection, is indicated by the black arrows. A geodesic on the grid which connects the two projections is then highlighted by purple edges (and boundaries for grid vertices). Red and yellow points, along with shaded green circles, represent backbone atoms and their estimated "radii", coherently with the color-scheme of Figure 2 in the main text. The colored rectangles correspond to the subgraphs X_G^k, for different values of k. On the right of each panel, we also show the grid path projected onto the mapper graph $\mathcal{M}$, to exemplify how the connectivity of the mapper graph reflects the one on X_G. As in the Supplementary Methods, δ represents the step of the grid on $\mathbb{S}_1$. (a) In this situation, the path of the conducting ion at two consecutive times is well approximated by the grid. The difference in the paths' lengths is just given by the discretization error. (b) In this situation, the path of the conducting ion at two consecutive times cannot be approximated by the grid. The ion goes through a small passage between two backbone atoms, which is not captured by the grid, as it is not fine enough. Therefore, the position of the ion at time t_i and at time t_{i+1} end up being in two different path connected components of the grid. (c) In this situation, the path of the conducting ion at two consecutive times cannot be faithfully approximated by the grid. The ion goes through a small passage between two backbone atoms, which is not captured by the grid, as it is not fine enough. Therefore, the grid-path needed to connect the position of the ion at time t_i and at time t_{i+1} end up being much longer than the distance between the two positions in $\mathbb{T}_3$.

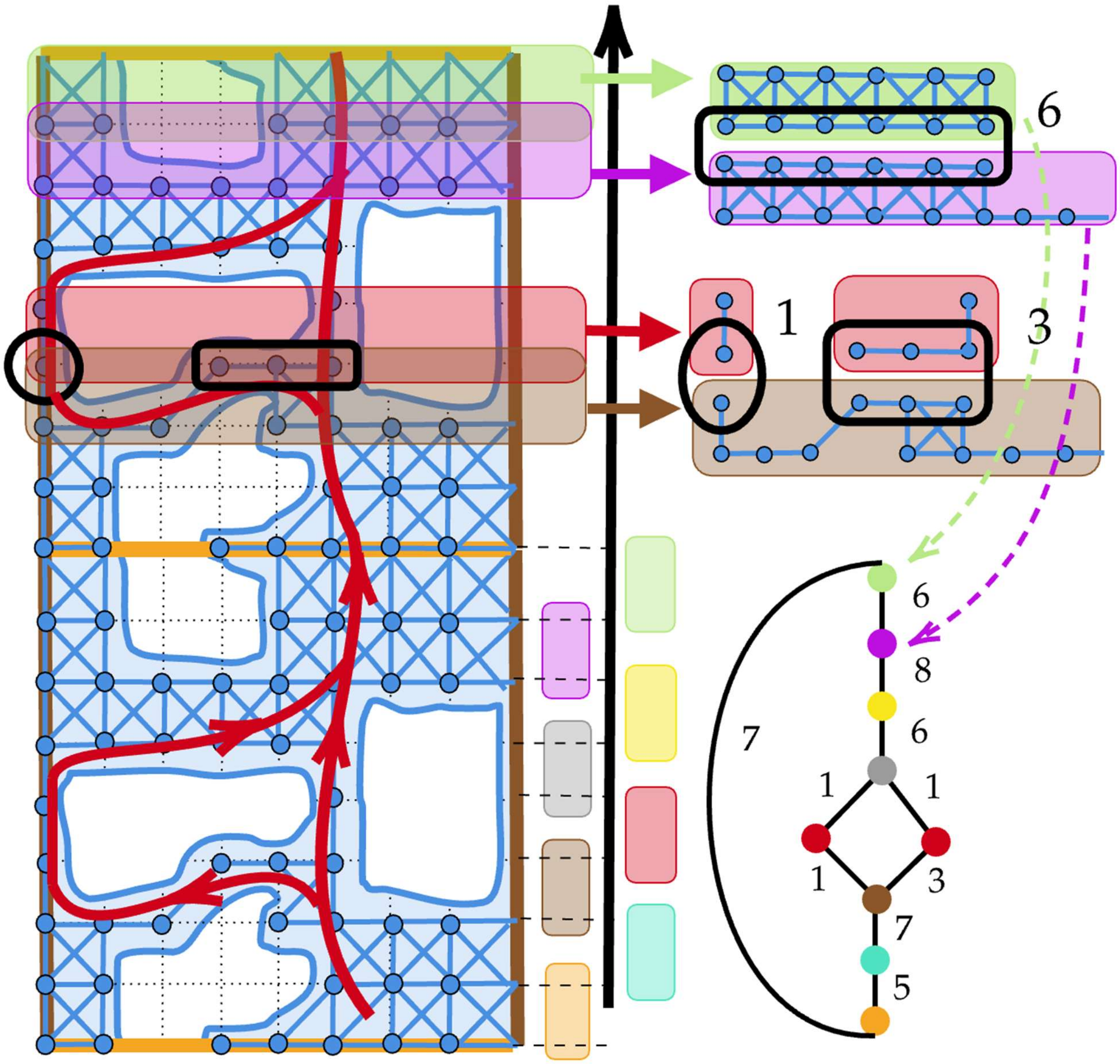

Supplementary Figure 4. Detailed depiction of the process described by Figure 2e in the main text. Starting from the bottom left corner of the figure, we see the graph approximating the void space, with the colored rectangle on the right representing the covering of the circle we use to compute the mapper graph. In the upper left corner, the subgraphs described in item 3 of Supplementary Methods section 5 are highlighted. In the upper right corner, instead, we highlight the path connected components of these subgraphs and their intersections. Lastly, in the lower right corner, we obtain the mapper graph and the weights we use as capacity constraints for computing the max-flow.

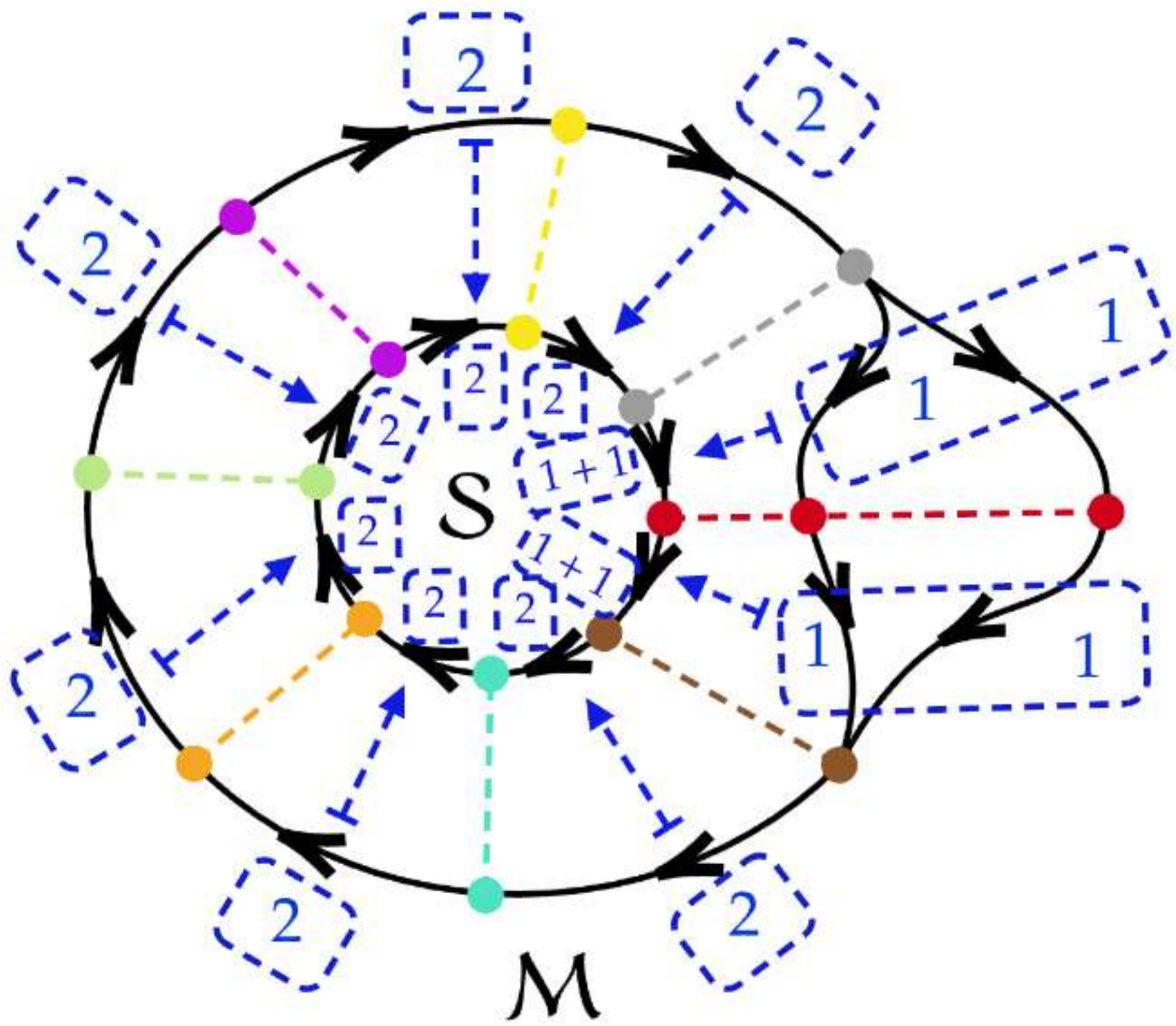

Supplementary Figure 5. Map $F_1(f)$, as defined in Supplementary Methods section 6, induced on circulations by the simplicial map *f* depicted in Supplementary Figure 4. Note that circulations on *Q* are constant on the edges.

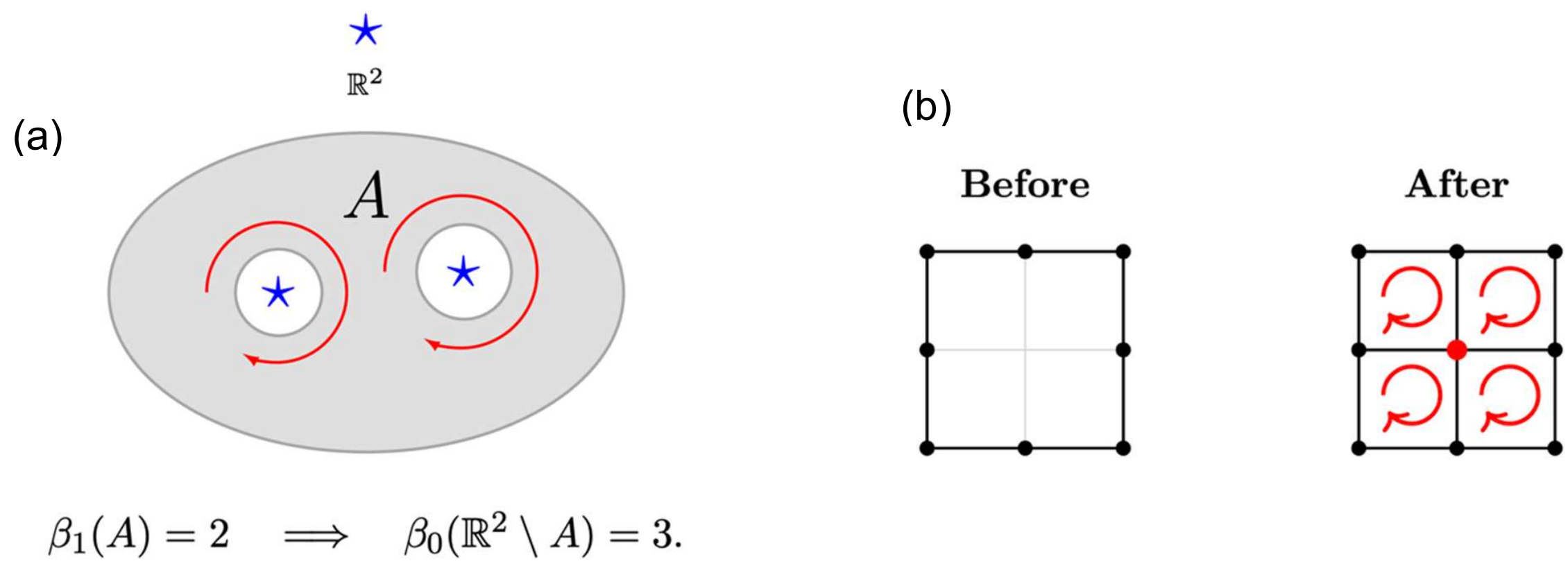

Supplementary Figure 6. (a) Planar Alexander-duality picture. The gray region is A. The two red curved arrows, drawn in A around the holes, indicate its two independent loops, while the three blue symbols mark the three connected components of $\mathbb{R}^2 \backslash A$: the two hole interiors and the unbounded exterior region. (b) Planar square-grid analogy. After adding the central occupied vertex, at most four new elementary square loops can appear around it, highlighted by the four red curved arrows in the "After" panel.

Topological intuition behind the toy complexity model. Panel (b): one additional local obstacle can create only a controlled number of new loops. Panel (a): in two dimensions, the number of connected components of the complement is governed by the number of holes in the obstacle set.

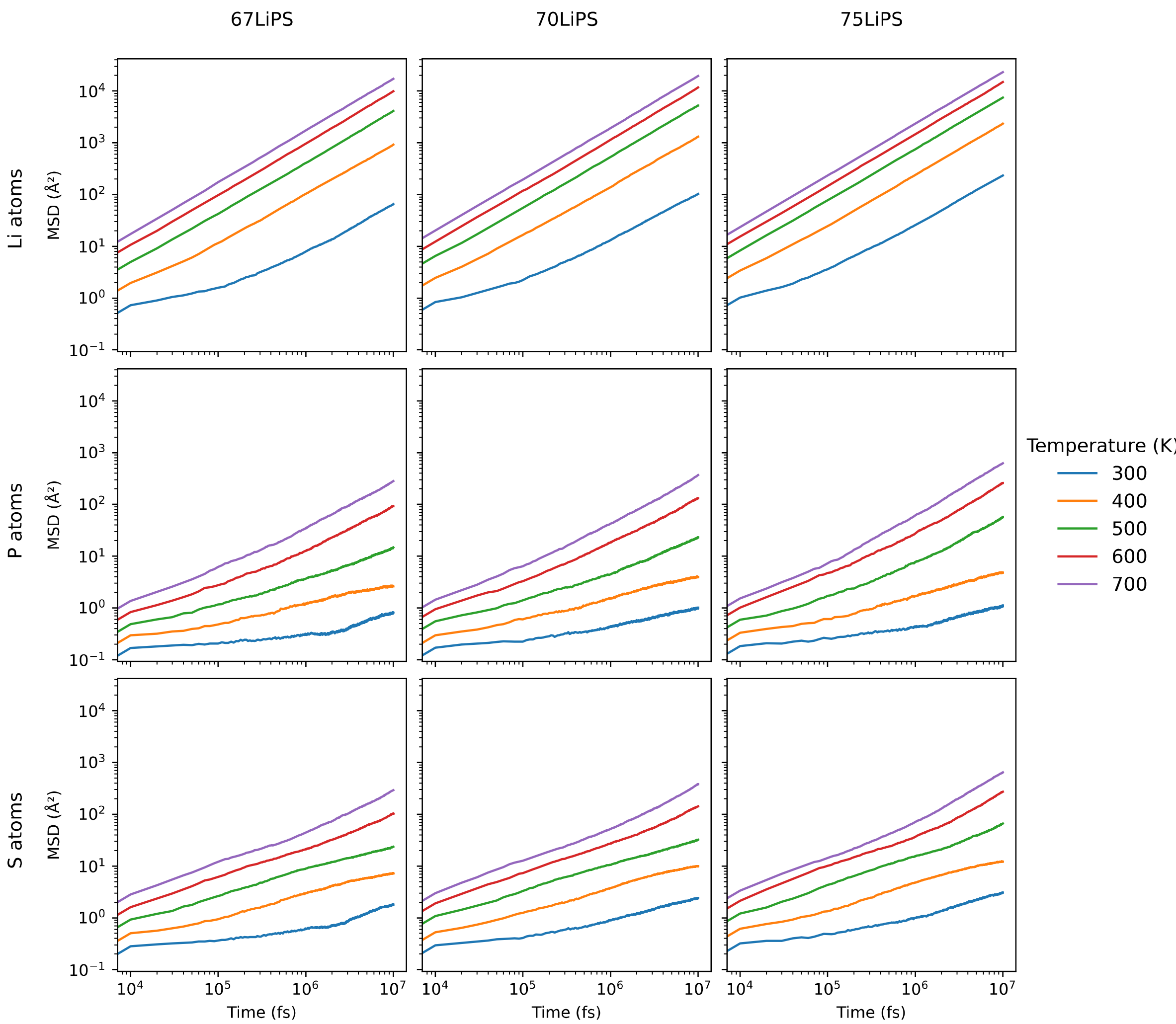

Supplementary Figure 7. Examples of mean squared displacement (MSD) of Li, P and S atoms in the three LiPS glasses (67LiPS, 70LiPS, and 75LiPS) sampled for 10 ns structures at target temperatures from 300 to 700 K. Source data are provided as a Source Data file.

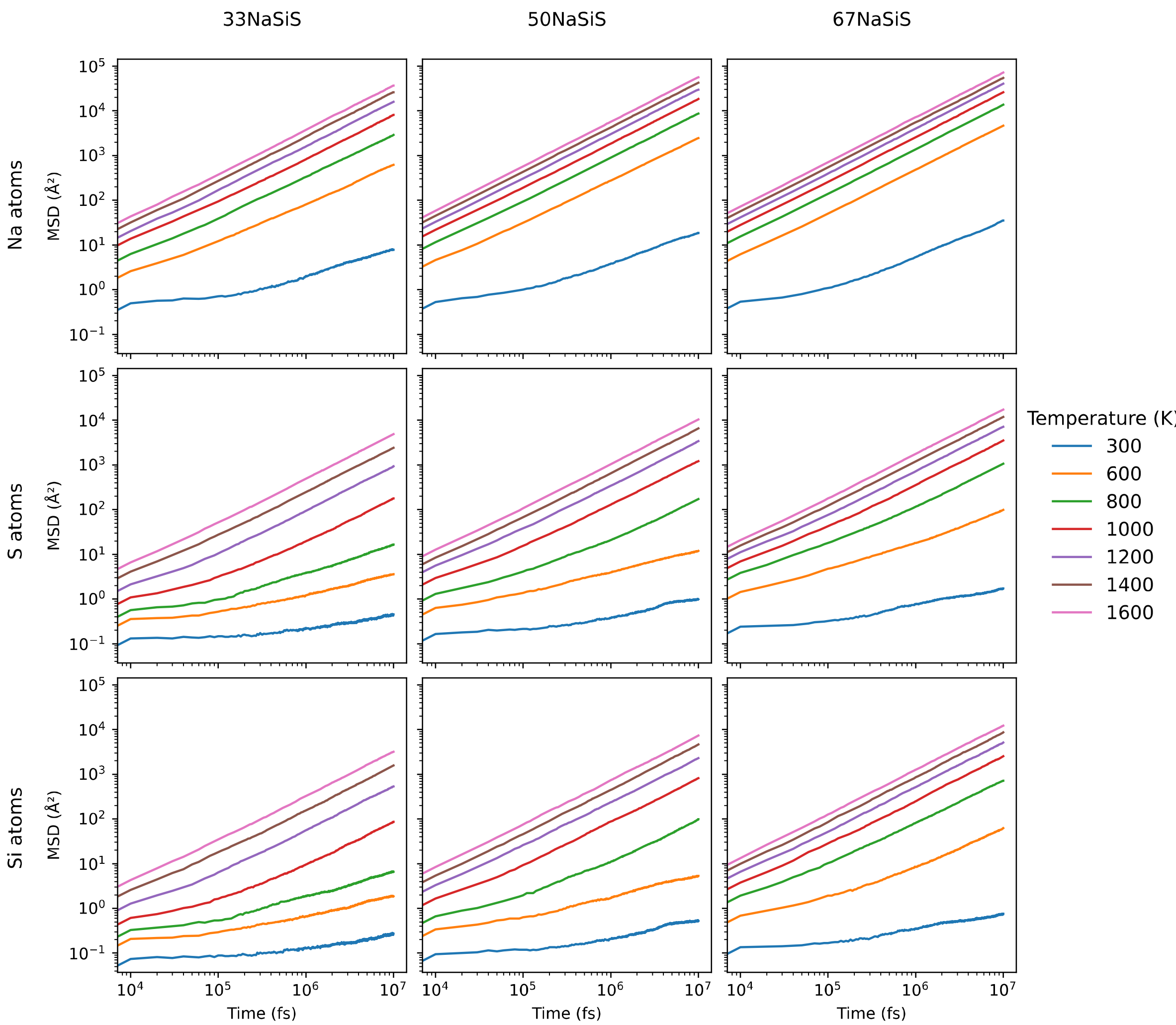

Supplementary Figure 8. Examples of mean squared displacement (MSD) of Na, S and Si atoms in the three NaSiS glasses (33NaSiS, 50NaSiS, and 67NaSiS) sampled for 10 ns structures at target temperatures from 300 to 1600 K. Source data are provided as a Source Data file.

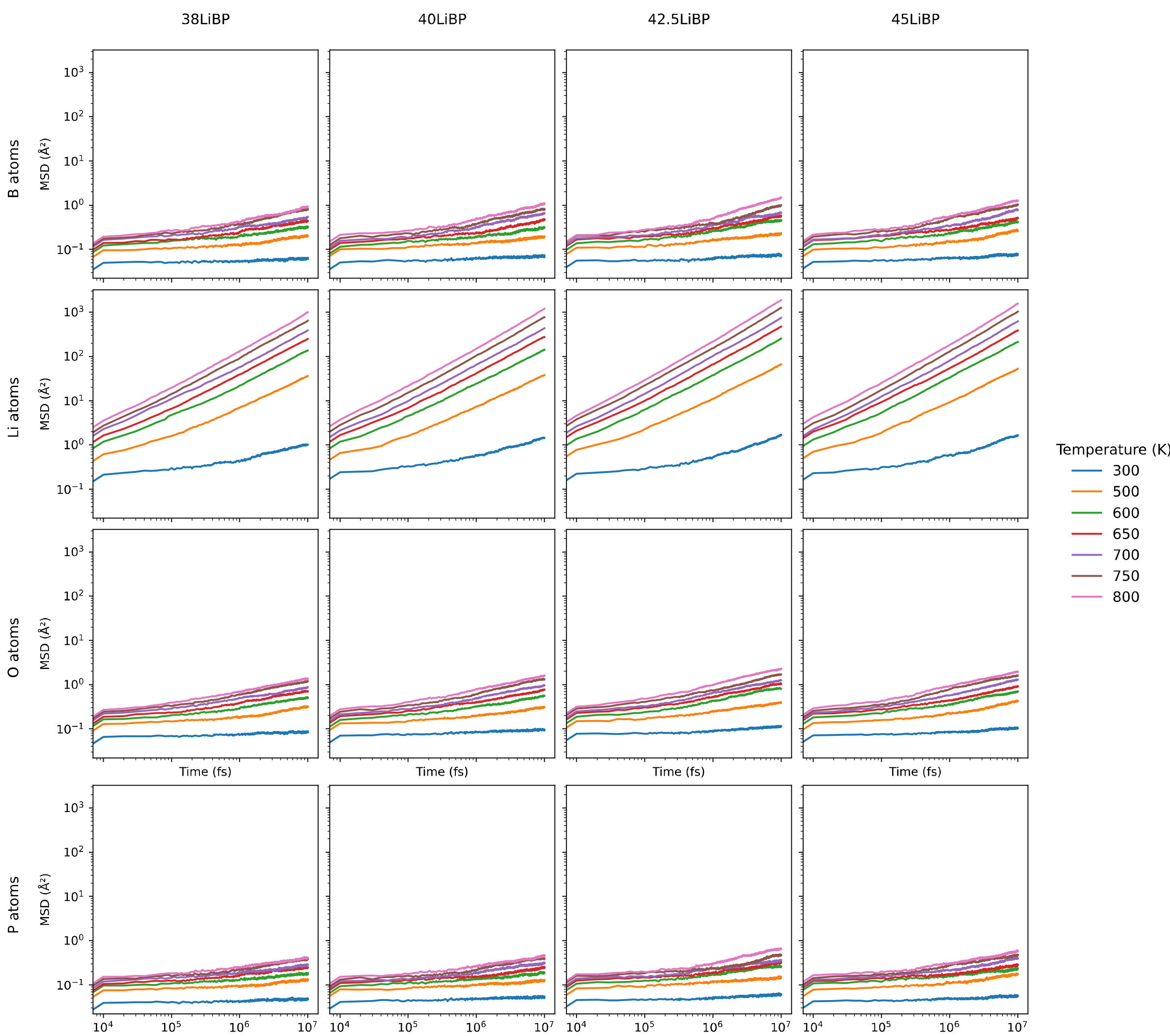

Supplementary Figure 9. Examples of mean squared displacement (MSD) of B, Li, O and P atoms in the four LiBP glasses (38LiBP, 40LiBP, 42.5LiBP, and 45LiBP) sampled for 10 ns structures at target temperatures from 300 to 800 K. Source data are provided as a Source Data file.

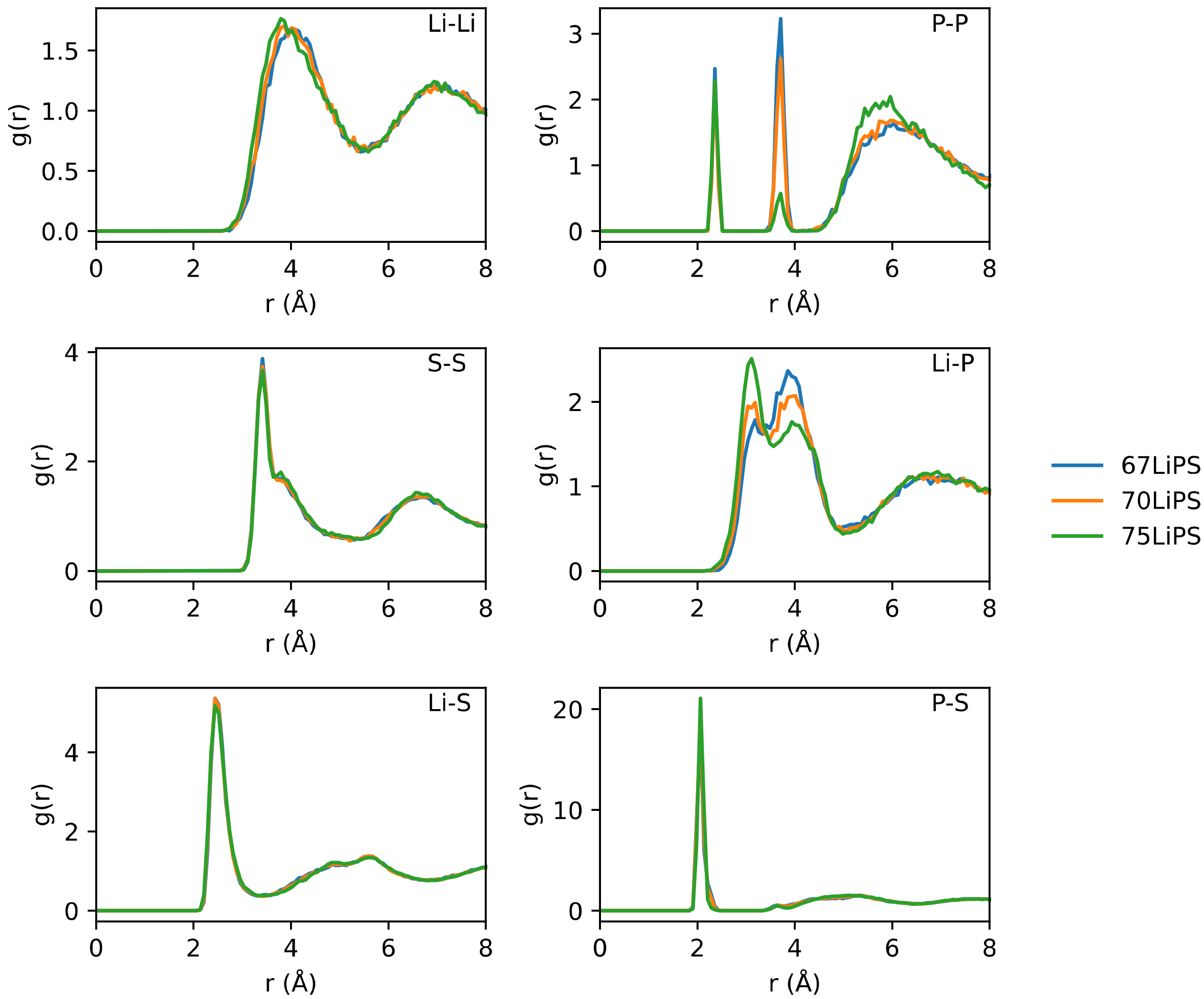

Supplementary Figure 10. Examples of radial distribution functions $g(r)$ for all pair-wise interactions of simulated glass structures in the three LiPS glasses (67LiPS, 70LiPS, and 75LiPS). Source data are provided as a Source Data file.

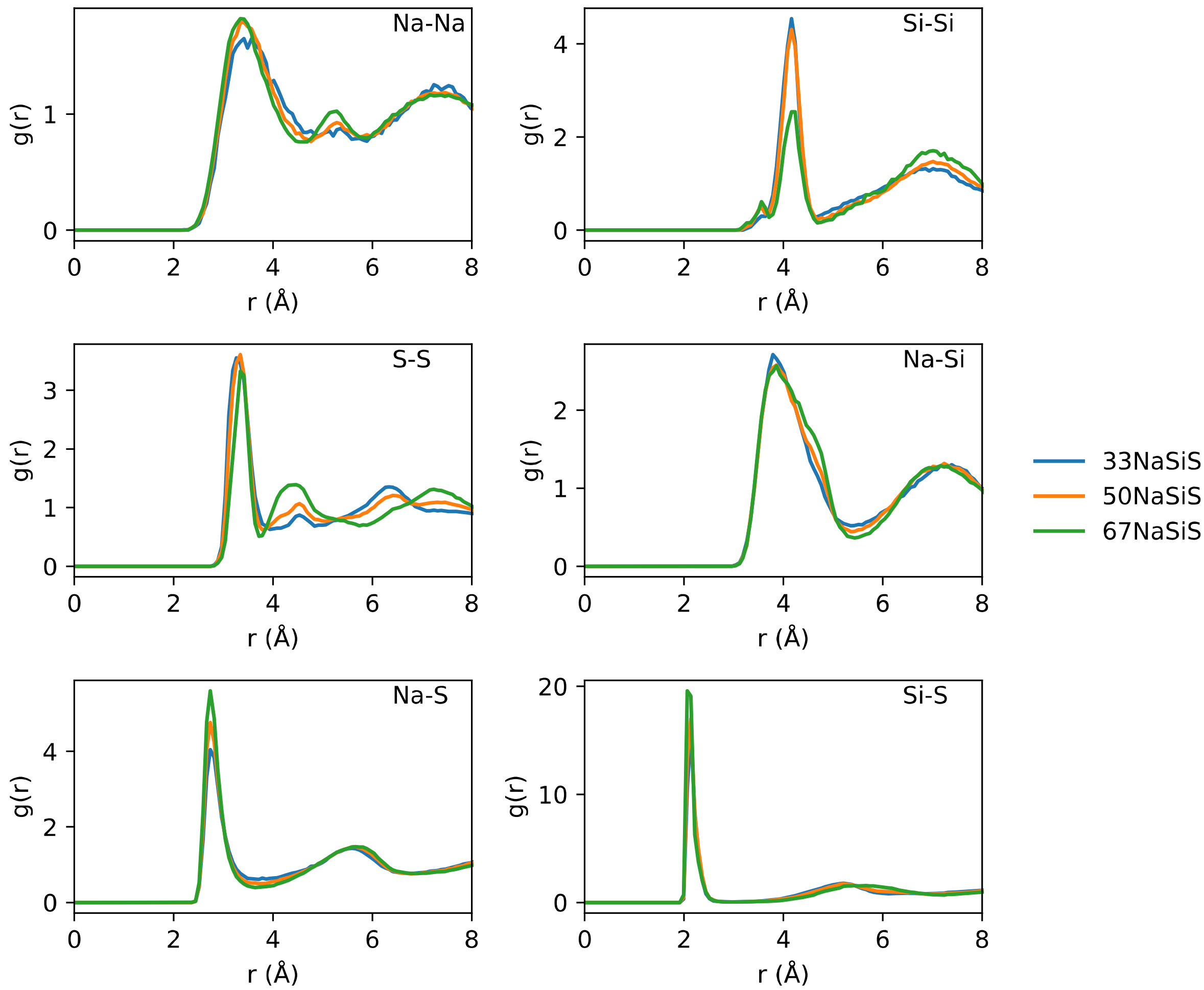

Supplementary Figure 11. Examples of radial distribution functions $g(r)$ for all pair-wise interactions of simulated glass structures in the three NaSiS glasses (33NaSiS, 50NaSiS, and 67NaSiS). Source data are provided as a Source Data file.

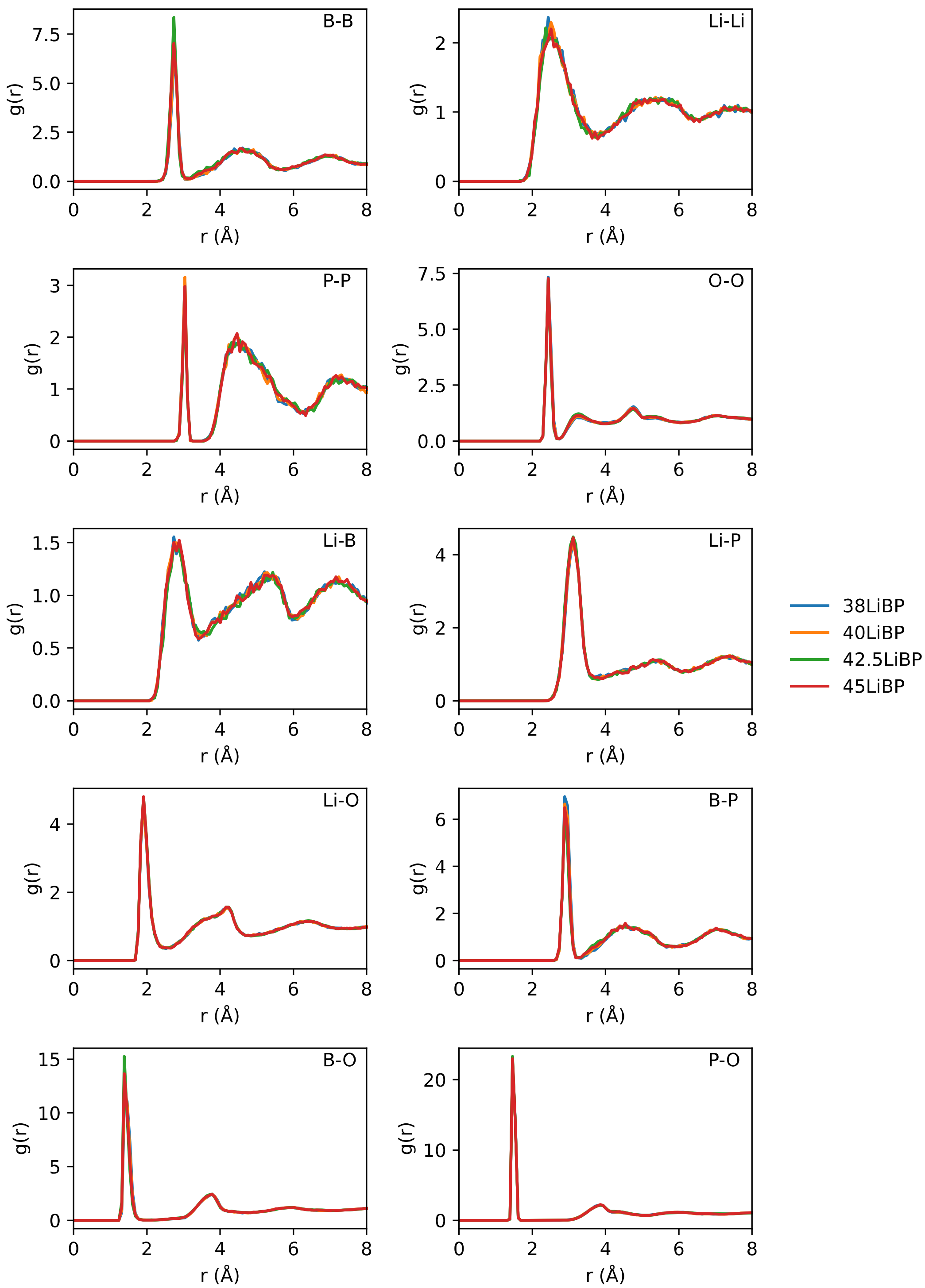

Supplementary Figure 12. Examples of radial distribution functions $g(r)$ for all pair-wise interactions of simulated glass structures in the four LiBP glasses (38LiBP, 40LiBP, 42.5LiBP, and 45LiBP). Source data are provided as a Source Data file.

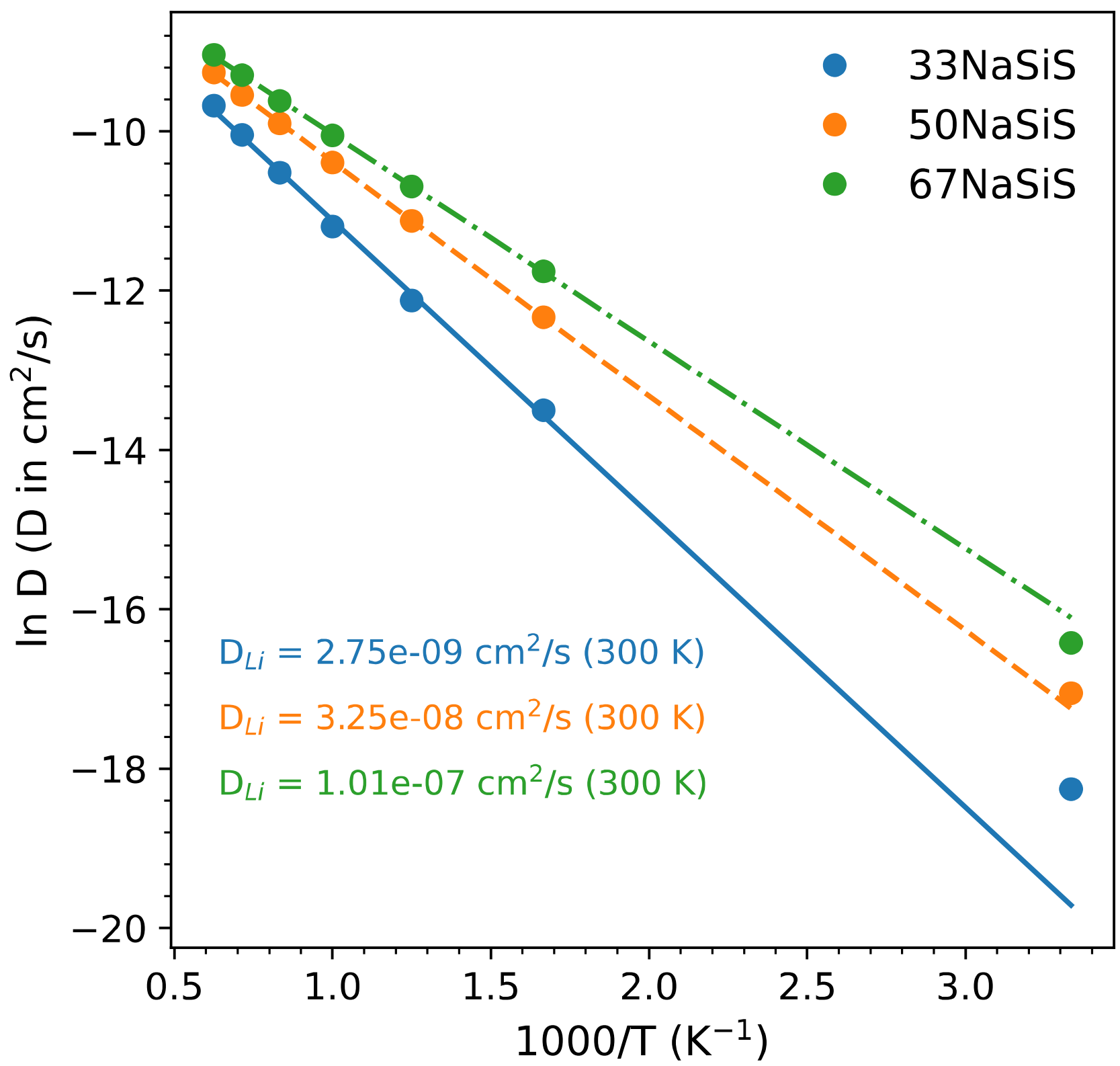

Supplementary Figure 13. Examples of Arrhenius plot of the diffusion coefficients of glasses in the NaSiS system sampled at elevated temperatures, and the extrapolated values at 300 K. Source data are provided as a Source Data file.

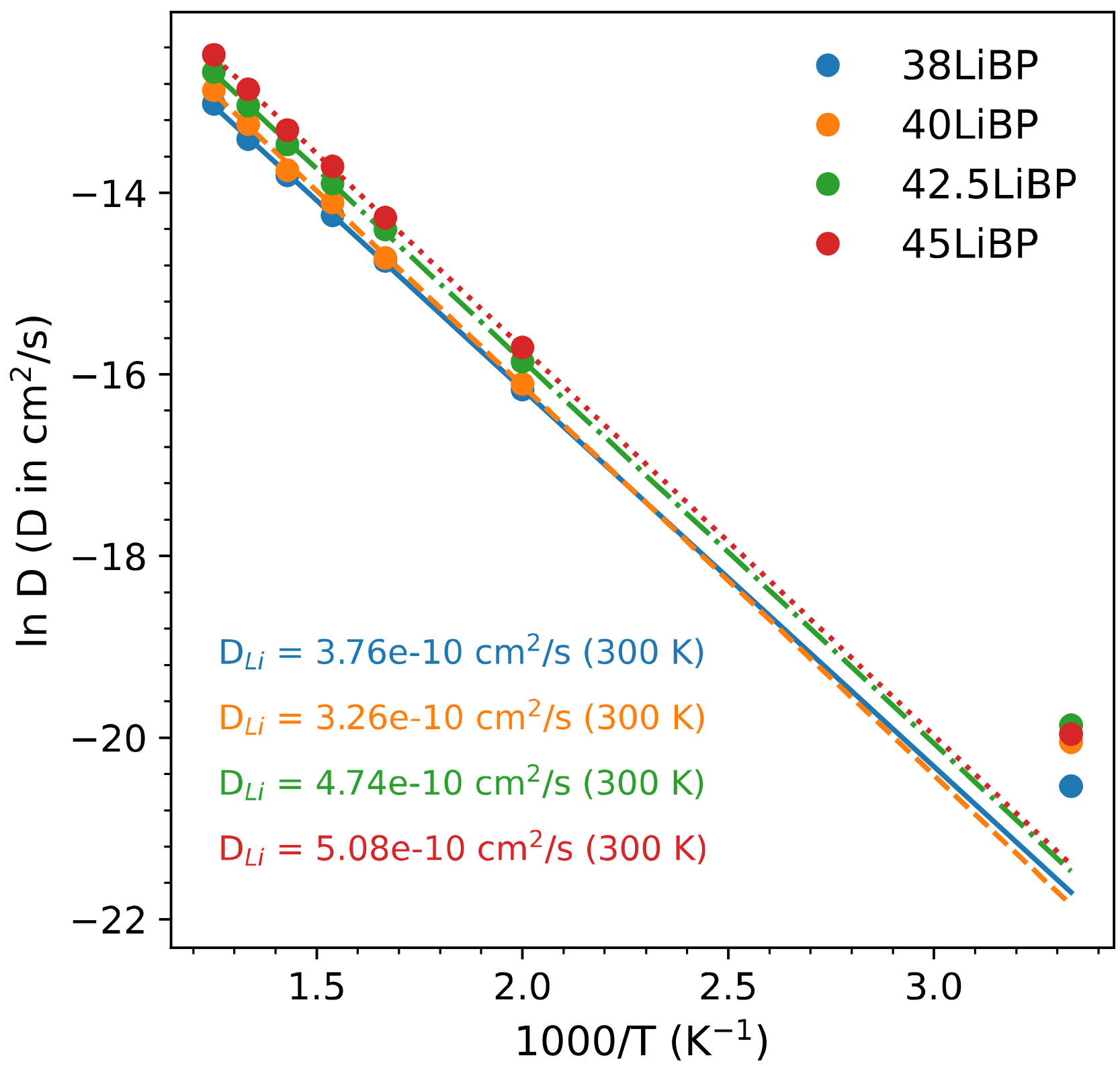

Supplementary Figure 14. Examples of Arrhenius plot of the diffusion coefficients of glasses in the LiBP system sampled at elevated temperatures, and the extrapolated values at 300 K. Source data are provided as a Source Data file.

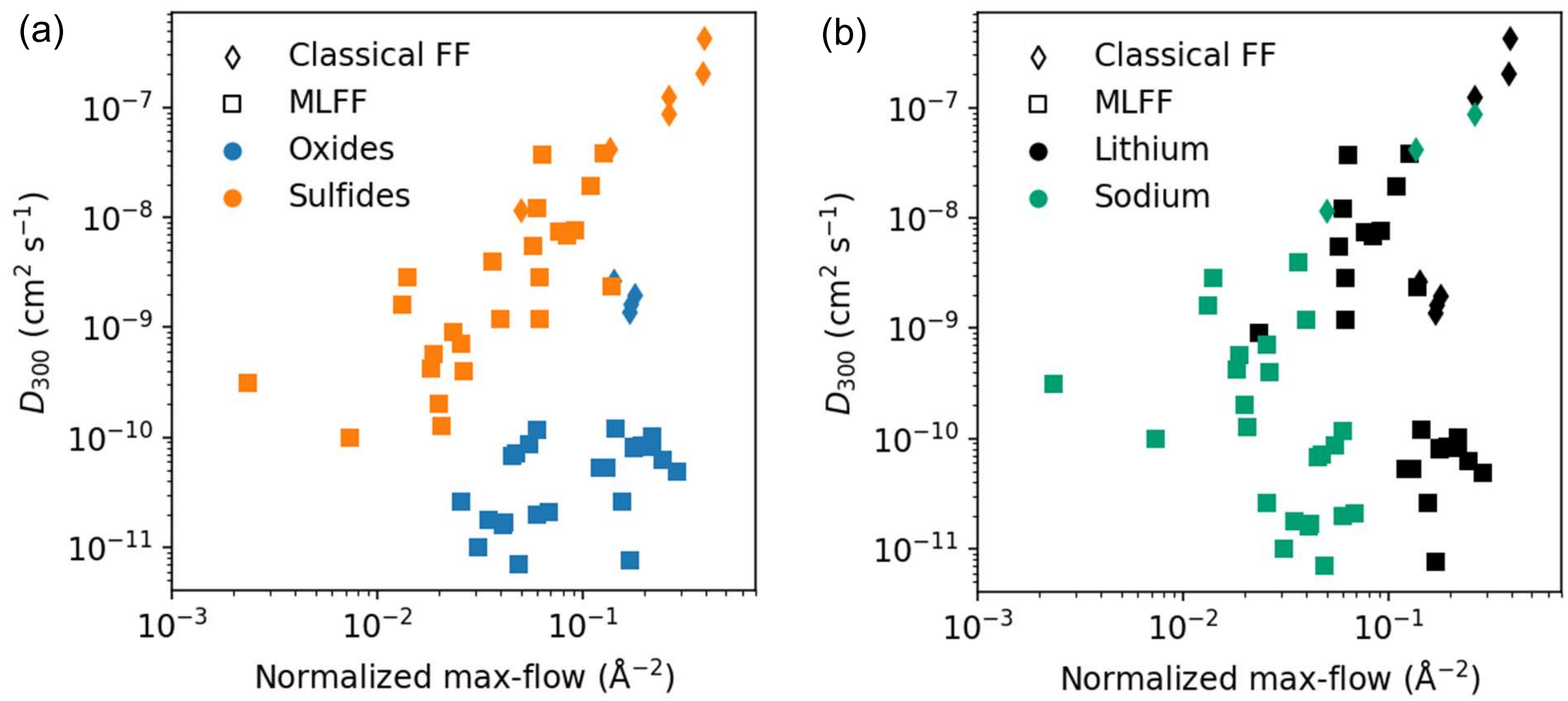

Supplementary Figure 15. Calculated room temperature diffusivity (D_{300}) of glasses plotted as a function of normalized max-flow as calculated from the static glass structures sorted. Results are categorized based on (a) glass family [blue: oxides; orange: sulfides] and (b) diffusing ion [black: lithium; green: sodium]. Diamonds denote glasses simulated by a classical force field and squares denote glasses simulated by a machine-learning force field (MLFF). Note that the axes of the plot are on a logarithmic scale.

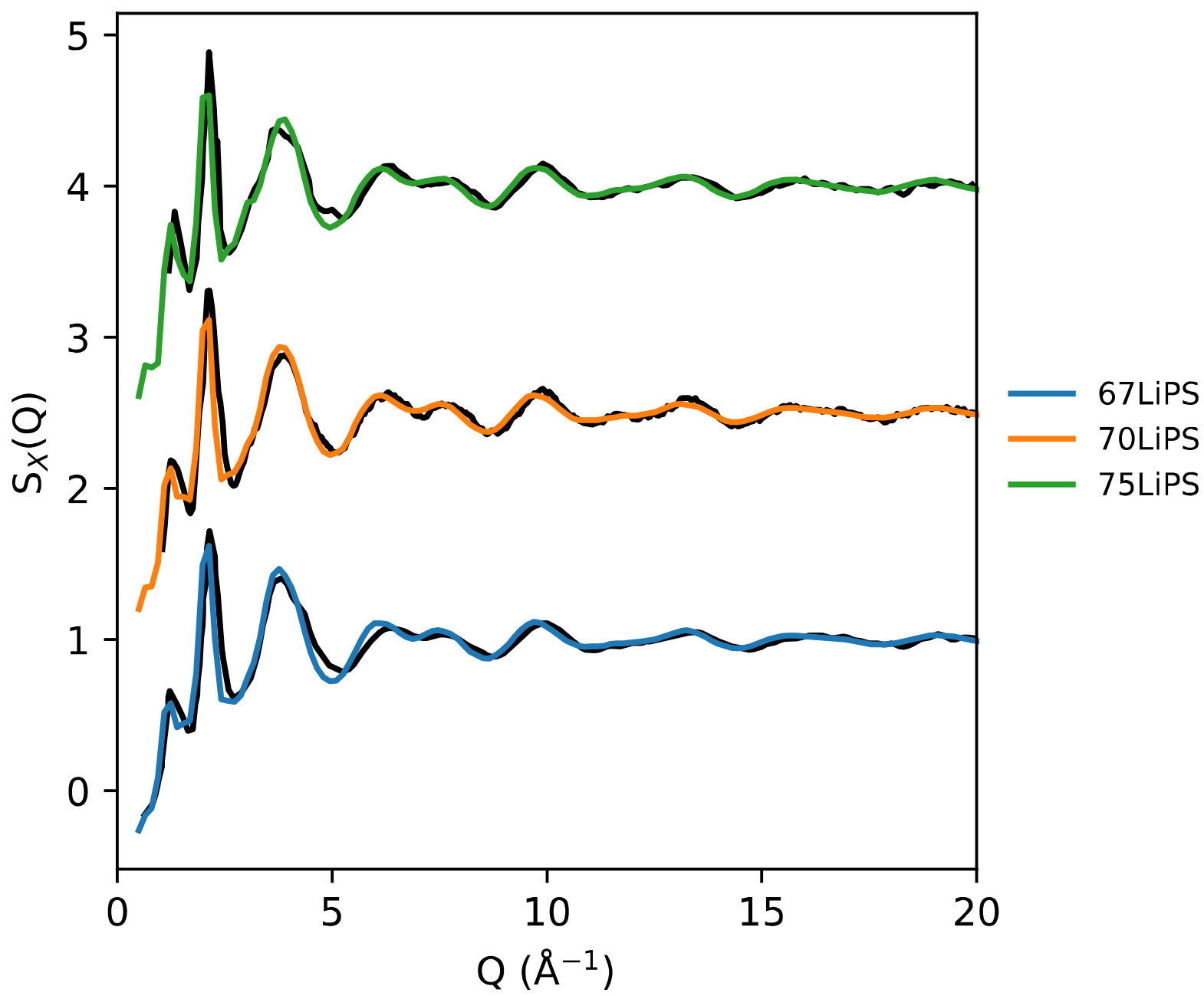

Supplementary Figure 16. X-ray structure factor $S_X(q)$ of LiPS glasses (67LiPS, 70LiPS, and 75LiPS). Experimental results shown as black lines are taken from ref. [8]. Source data are provided as a Source Data file.

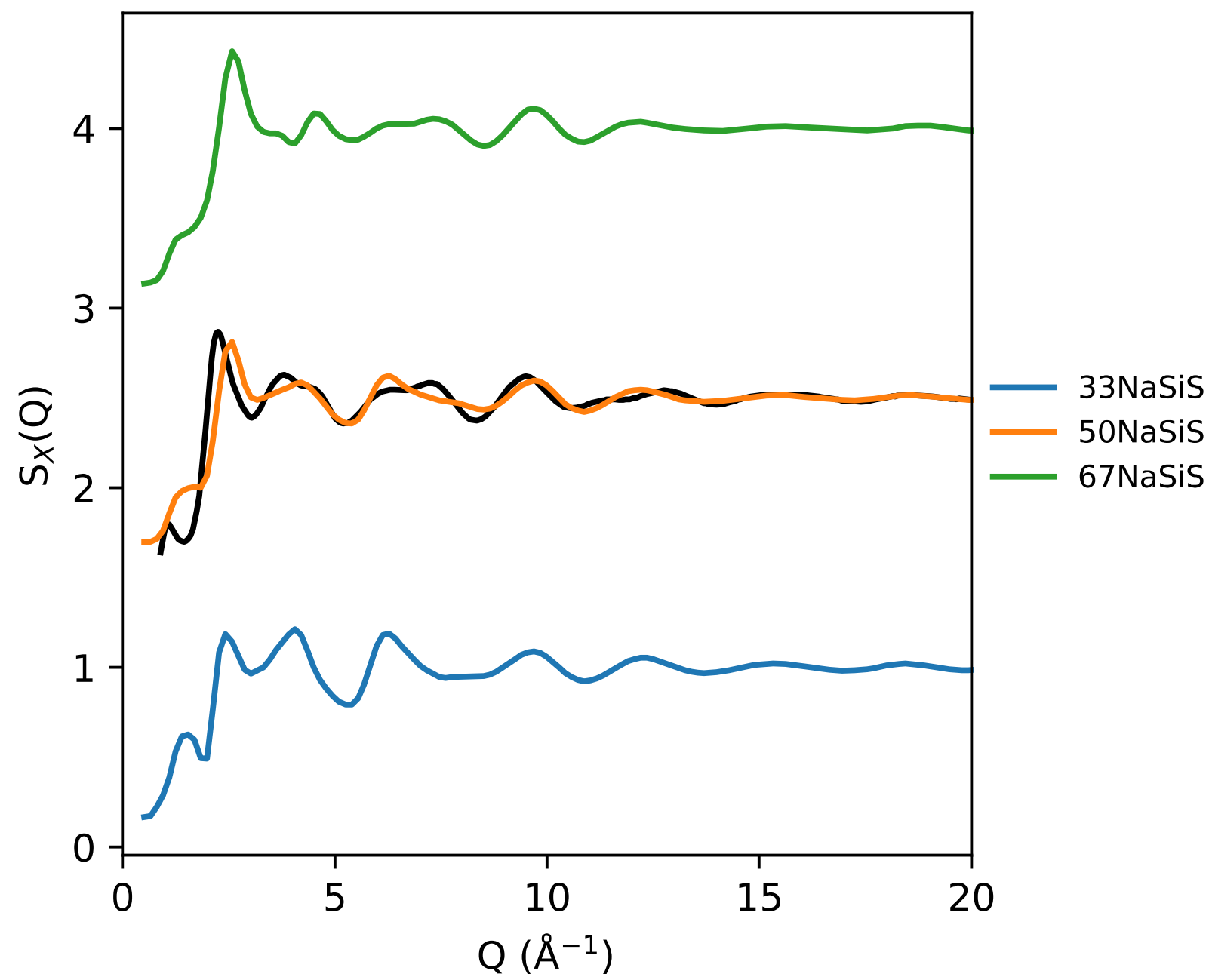

Supplementary Figure 17. X-ray structure factor $S_X(q)$ of NaSiS glasses (33NaSiS, 50NaSiS, and 67NaSiS). Experimental results shown as black lines are taken from ref. [9]. Source data are provided as a Source Data file.

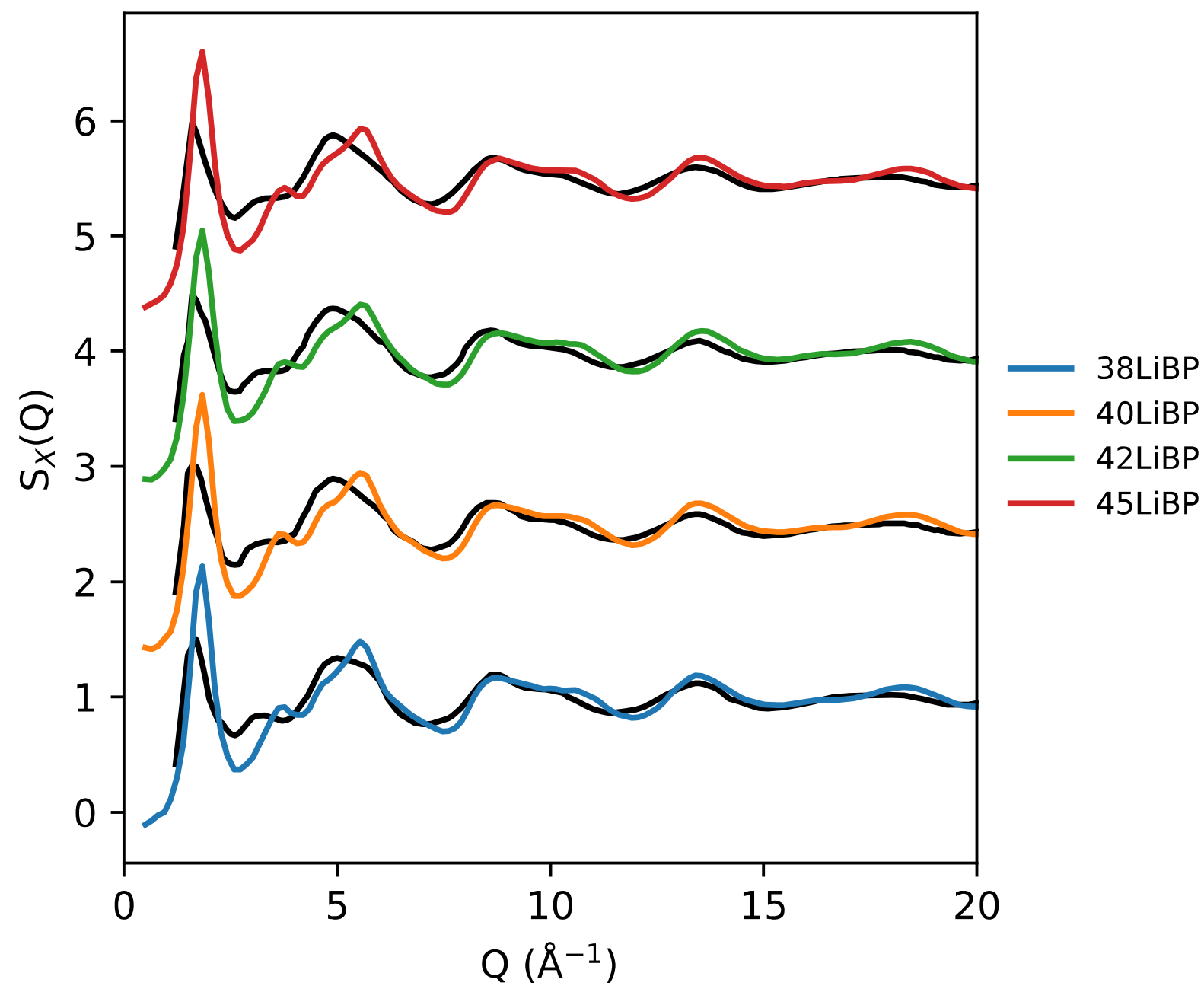

Supplementary Figure 18. X-ray structure factor $S_X(q)$ of LiBP glasses (38LiBP, 40LiBP, 42.5LiBP, and 45LiBP). Experimental results shown as black lines are taken from ref. [10]. Source data are provided as a Source Data file.

Supplementary References

[1] H. Karcher, Riemannian center of mass and mollifier smoothing. *Commun. Pure Appl. Math.* **30**, 509-541 (1977).

[2] M. Pegoraro, L. Fajstrup, Circular Max-Flow for Periodic Data via Reeb Graphs. *arXiv preprint* arXiv:2312.04357 (2023).

[3] E. W. Chambers, J. Erickson, A. Nayyeri, Homology flows, cohomology cuts. *SIAM J. Comput.* **41**, 1605-1634 (2012).

[4] R. Tarjan, Depth-First Search and Linear Graph Algorithms. *SIAM J. Comput.* **1**, 146-160 (1972).

[5] T. H. Cormen, C. E. Leiserson, R. L. Rivest, C. Stein, *Introduction to Algorithms*, 3rd edition (MIT Press, Cambridge, 2009).

[6] A. Hatcher, *Algebraic topology* (Cambridge University Press, Cambridge, 2000).

[7] K. I. Cho, S. H. Lee, K. H. Cho, D. W. Shin, Y. K. Sun, Li_2O–B_2O_3–P_2O_5 solid electrolyte for thin film batteries. *J. Power Sources* **163**, 223-228 (2006).

[8] K. Ohara, A. Mitsui, M. Mori, Y. Onodera, S. Shiotani, Y. Koyama, Y. Orikasa, M. Murakami, K. Shimoda, K. Mori, T. Fukunaga, H. Arai, Y. Uchimoto, Z. Ogumi, Structural and electronic features of binary Li_2S-P_2S_5 glasses. *Sci. Rep.* **6**, 21302 (2016).

[9] A. Dive, C. Benmore, M. Wilding, S. W. Martin, S. Beckman, S. Banerjee, Molecular Dynamics Modeling of the Structure and Na^+-Ion Transport in Na_2S + SiS_2 Glassy Electrolytes. *J. Phys. Chem. B* **122**, 7597-7608 (2018).

[10] Z. Chen, T. Du, S. S. Sørensen, R. Christensen, Q. Zhang, L. R. Jensen, O. V. Magdysyuk, M. Diaz-Lopez, M. Bauchy, Y. Z. Yue, M. M. Smedskjaer, Structure dependence of fracture toughness and ionic conductivity in lithium borophosphate glassy electrolytes for all-solid-state batteries. *J. Power Sources* **553**, 232302 (2023).
