# Supplementary material for: Predicting ionic conductivity of sulfide glass solid-state electrolytes using max-flow methodology
Source: Nat Commun. 2026 Aug 31;17:10343. doi: 10.1038/s41467-026-77363-w (PMC13623887; doi:10.1038/s41467-026-77363-w)
Supplement: Supplementary file 2 [file 41467_2026_77363_MOESM2_ESM.pdf]

nature portfolio

Peer Review File

Predicting ionic conductivity of sulfide glass solid-state electrolytes using max-flow methodology

Corresponding Author: Professor Morten Smedskjaer

This file contains all reviewer reports in order by version, followed by all author rebuttals in order by version.

Version 0:

Reviewer comments:

Reviewer #1

(Remarks to the Author)
This is an excellent study that proposes a new framework that is capable of summarising the topological properties of a solid electrolyte material's atomic structure with a weighted graph based on periodic boundary conditions. The authors then apply the max-flow algorithm to this summary. The novel findings are then further validated by comprehensive molecular dynamics simulations. I am somewhat surprised to see that MLFFs were not used for the simulations given the author's excellent work in this area but I do not feel this has a significant impact on the overall findings or validation. I have no hesitation in recommending the publication of this work as it is likely to be of significance to the field and indeed other related fields.

(Remarks on code availability)

Reviewer #2

(Remarks to the Author)
The manuscript presents an original and interesting approach to predicting ionic conductivity in glassy solid-state electrolytes by employing a novel adaptation of the max-flow algorithm on graph representations of static atomic structures. By leveraging this graph-theoretic framework, the authors claim to offer a method that circumvents the need for extensive molecular dynamics simulations, which are often time- and resource-intensive. The methodology is innovative and seems to provide a generalizable and computationally efficient alternative for screening candidate electrolyte materials across different chemistries. The correlation between predicted maximum flow and simulated ion diffusivity is examined across a few glass families, underscoring the potential of this method in materials discovery.

While the proposed method is original and the results seem promising, the manuscript would benefit from a more thorough contextualization of the approach with respect to existing state-of-the-art methodologies in the field. In particular, the potential impact of the work remains somewhat unclear due to the lack of a direct, quantitative comparison with other established techniques for evaluating ionic conductivity in disordered systems. Additionally, certain assumptions, such as the exclusion of network atom mobility and the universality of backbone-based representations, require further clarification and discussion. These aspects are detailed in the following comments and should be addressed to better convey the broader applicability and limitations of the approach. Moreover, given the technical depth and methodological focus of the study, the work may be more appropriately targeted to a specialized journal such as NPJ Computational Materials, where its computational novelty and materials-science relevance can be fully appreciated.

1/ In the introduction, the authors briefly mention the use of the Bond Valence method to study ionic diffusion in solids, amongst other more computationally intensive approaches. However, several other established or emerging approaches aimed at predicting ionic conductivity (particularly those designed to reduce computational cost while maximizing predictive accuracy) are not adequately discussed. For example, Refs. [DOI: 10.1063/1.4960790], [DOI: 10.1038/s41524-023-01051-2], [DOI: 10.1038/s41524-022-00877-6], [DOI: 10.1038/s41597-020-0491-x], [DOI: 10.1103/PhysRevMaterials.2.065405],and [DOI: 10.1063/1.4960790] offer alternative frameworks that share similar goals with the present work and should be acknowledged. A critical comparison between these methods and the proposed max-flow framework is missing and would be valuable to properly contextualize the contribution. Specifically, it remains unclear how the current approach compares in terms of predictive performance, computational efficiency, and applicability across different material systems. A more

comprehensive discussion that highlights the strengths and limitations of the proposed methodology relative to existing techniques is essential.

2/ The generation of the Fréchet mean and the definition of the spatial region accessible to ion diffusion are central steps in the proposed methodology. However, this process appears to rely on radial distribution functions, which require molecular dynamics simulations to compute. This raises a fundamental concern: a major motivation of the study is to avoid such computationally intensive simulations, yet the methodology seems to depend on them to define the diffusion space. It is unclear at what point this computational burden is genuinely reduced. If only short MD runs are used, there is a risk that the resulting void space is poorly defined or unrepresentative. Conversely, if long MD simulations are required to reliably characterize the diffusion space, one might question why not directly extract conventional transport metrics from the trajectories instead. This apparent contradiction should be addressed more clearly. The authors should specify the extent of MD required and provide a justification for how the proposed method reduces overall computational cost in practice, relative to standard MD-based analysis.

3/ Related to the previous point, it remains unclear how the proposed method would perform in scenarios where the mobility of the anionic framework (assumed to be relatively static in this work) or strong cation–anion coupling plays a significant role in ion transport. Such assumptions may limit the applicability of the method, particularly as recent studies increasingly highlight the importance of dynamic anion behavior and correlated cation–anion motion in highly conductive glassy systems, including halide-based electrolytes (see, for example, [DOI: 10.1002/anie.202315628], [DOI: 10.1002/adfm.202410509]). A more detailed discussion of how the method accounts for (or fails to capture) these complex dynamical effects is warranted. In particular, the authors should clarify the implications of their semi-static structural assumptions on predictive accuracy in systems where the backbone mobility or cation–anion interactions are non-negligible.

4/ The graph-based representation assumes that the weights on the edges reflect the maximum number of ions that can flow through each edge, effectively treating the conduction network as a capacity-limited flow system. However, this assumption may be problematic, as it oversimplifies the physical nature of ion diffusion. In reality, the viability of a diffusion path is governed not only by geometric constraints but also by energy barriers, which can vary significantly across the structure and do not necessarily correlate with the number of ions that can theoretically pass through a given region. This raises concerns about whether the edge weights in the max-flow model accurately capture the true transport limitations within the material. The potential disconnect between geometric flow capacity and energy barrier-driven diffusion could result in misleading conclusions about conductivity. This conceptual issue should be addressed more thoroughly. The authors are encouraged to clarify how, or to what extent, energetic factors are implicitly or explicitly accounted for in their model, and to discuss the limitations of using purely topological or geometric criteria to infer ion mobility.

5/ The results shown in Figure 4 demonstrate encouraging qualitative agreement between the predicted maximum flow values and the observed trends in ionic diffusivity across different material families. However, given the small dataset, the quantitative predictive power of the method remains unclear. Specifically, can the max-flow values be systematically calibrated or scaled to yield quantitative predictions of ionic conductivity or diffusivity, rather than just relative rankings? Addressing this question would significantly enhance the utility of the approach for practical materials screening. A discussion on whether such a calibration is feasible (either globally or within specific chemical families) and what limitations or uncertainties might be involved, would be valuable. Furthermore, exploring the possibility of generalizing this framework to allow for predictive simulations across a broader range of systems could strengthen the impact and applicability of the method.

6/ With respect to the glass structures, while supplementary figure 16 shows good agreement between the computed and reference structures, this level of consistency does not appear to generalize across all systems. In particular, supplementary figures 17 and 18 reveal noticeable discrepancies, especially in the position and relative intensity of certain features. These differences raise questions about the robustness and transferability of the structural representation across different material families.

7/ Please provide more details regarding the computational cost and scalability of the proposed max-flow algorithm, particularly with respect to system size and complexity. Understanding how the algorithm performs as the number of atoms or the size of the simulation cell increases is important for assessing its practical applicability in large-scale screening efforts. Information such as computational time, memory usage, and parallelizability would help in this regard.

(Remarks on code availability)
The associated code provided at https://github.com/pego91/circular_max_flow has not been thoroughly evaluated in detail; however, from a general inspection, the repository appears to be reasonably well-organized. The directory structure seems clear, and the use of modular Python scripts facilitates readability and potential reuse. That said, it is recommended to explicitly add a detailed README file at the root (include project overview, installation instructions, usage examples, input/output formats, and expected results). Additionally, more comprehensive inline documentation and user-facing examples or tutorials would further enhance the accessibility and reproducibility of the workflow, particularly for researchers outside the immediate field.

Reviewer #3

(Remarks to the Author)
This article presents a novel computational approach for modeling and predicting ionic conductivity in glassy solid-state

electrolytes using max-flow methodology. The work addresses a significant challenge in battery research by proposing an approach that could accelerate the discovery of high-performance electrolyte materials. The authors successfully adapt graph theory concepts to glasses. The methodology is well-motivated by drawing parallels between ion transport and classical network flow problems.

The following comments can be made regarding this work:

On page 8, when discussing the difference between the calculated and experimental ionic conductivity values, discrepancies exist between mentioned values. However, the discussion of the potential reasons for this is insufficient and requires more details.
A more detailed comparison with the MD simulation data should be included in the supplementary materials if possible.
The caption for Supplementary Figure 6 in the supplementary materials should refer to Supplementary Figure 5 (which is an enlargement of Figure 2e from main text).

After these minor revisions, the article can be recommended for publication in Nature Communications.

(Remarks on code availability)

Reviewer #4

(Remarks to the Author)
This work presents an original methodology that adapts the max-flow algorithm, commonly used in continuum models for fluid and transport phenomena, to study ion transport in glassy inorganic solid electrolytes. The approach accounts for periodic boundary conditions (PBC) and uses flow network theory to quantify ion-conducting pathways from static atomic structures. This suggests that the max-flow metric could serve as a computationally efficient structural descriptor for predicting ionic conductivity in glassy SSEs. It is an interesting and novel contribution to method development.
However, its application is constrained by key assumptions and some other limitations listed below, which may hinder its accessibility, reproducibility, and broader adoption within molecular modelling community. I recommend that the work be considered in more specialised computational journal.
(1) The model assume that backbone is static, and ions motion follows Arrhenius relationship. This assumption is valid for many solid inorganic glassy systems below the Tg. However, it is not for a broad range of polymer electrolytes or hybrid systems where the backbone exhibits significant segmental motion near or above Tg. In such systems, backbones are coupled with ion transport and conductivity shows non-Arrhenius behaviour. The authors should discuss whether the proposed method remains valid or meaningful under such conditions.
(2) The model predict ionic flux based purely on void space geometry, without incorporating energetic interactions between mobile ions and the surrounding matrix. For example, for two different polymer systems with identical void geometry, will the model predict the same conductivity?
(3) While the max-flow framework is conceptually simple, its practical implementation requires familiarity with graph theory and network optimization — skills not commonly taught in traditional molecular modeling or materials simulation curricula. This may limit its accessibility to researchers without computational graph modeling experience, and highlights the need for well-documented tools and workflows to support broader application.
(4) Calculation of diffusion coefficients using KINISI package involves Bayesian linear regression and MCMC sampling, which offers uncertainty quantification but introduces additional complexity. Reproducing this workflow requires familiarity with probabilistic modeling and parameter tuning, which may be a barrier for users accustomed to classical linear MSD fitting approaches.

(Remarks on code availability)
Python codes are available . However, no step-by-step instructions are available to advice readers how to run codes to reproduce this work. This is challenging for people without relevant background.

Version 1:

Reviewer comments:

Reviewer #1

(Remarks to the Author)
The authors have fully addressed any minor comments I had. The manuscript is ready for publication.

(Remarks on code availability)
The improvements in readability and application of the code should be much appreciated by the community.

Reviewer #2

(Remarks to the Author)
The authors’ substantial efforts to revise the manuscript are noted and appreciated. They have significantly expanded the materials, analyses, and explanatory detail, and have successfully addressed all previous concerns. The manuscript seems

now suitable for publication.

(Remarks on code availability)

Reviewer #4

(Remarks to the Author)
The revised manuscript has improved substantially, particularly through the expanded dataset, the narrower focus on sulfide glass electrolytes, and the improved code-availability information. I have two minor suggestions that may help further clarify the scope and reproducibility of the revised work.

1. The manuscript should more clearly define when the max-flow descriptor is expected to be valid. In particular, it should state that the method is most appropriate when the host/network structure is effectively static on the ion-transport timescale, and that dynamically disordered systems such as polymer or hybrid electrolytes require separate validation.
2. The authors acknowledge in the response letter that the current method is mainly based on void-space geometry/connectivity after the RDF-derived void definition is fixed. This limitation should also be stated explicitly in the manuscript. Systems with similar void connectivity but different ion-binding strengths or activation barriers may yield similar max-flow values unless those energetic differences are indirectly reflected in the static structure or RDF-derived void definition.

(Remarks on code availability)

Version 2:

Reviewer comments:

Reviewer #4

(Remarks to the Author)
I don't have any further question.

(Remarks on code availability)
It looks fine to me. But I didn't; install and run it myself.

Open Access This Peer Review File is licensed under a Creative Commons Attribution 4.0 International License, which permits use, sharing, adaptation, distribution and reproduction in any medium or format, as long as you give appropriate credit to the original author(s) and the source, provide a link to the Creative Commons license, and indicate if changes were made.

In cases where reviewers are anonymous, credit should be given to 'Anonymous Referee' and the source.
The images or other third party material in this Peer Review File are included in the article's Creative Commons license, unless indicated otherwise in a credit line to the material. If material is not included in the article's Creative Commons license and your intended use is not permitted by statutory regulation or exceeds the permitted use, you will need to obtain permission directly from the copyright holder.
To view a copy of this license, visit https://creativecommons.org/licenses/by/4.0/

Point-by-Point Response Letter

We thank all Reviewers for their comments on our manuscript. We have made an extensive effort to revise the manuscript in accordance with the suggestions of the Reviewers. Detailed point-by-point responses to the Reviewers' comments are given below.

We note that additional simulations and analyses have been performed to address the Reviewers' comments. As such, we have reordered the author list (for the first four authors) to reflect the contributions during the revision stage and included an "equal contribution" note for the first three authors. All other co-authors have agreed with this change, and we also note that no authors have been added or removed during the revision.

Reviewer #1 (Remarks to the Author):

This is an excellent study that proposes a new framework that is capable of summarising the topological properties of a solid electrolyte material's atomic structure with a weighted graph based on periodic boundary conditions. The authors then apply the max-flow algorithm to this summary. The novel findings are then further validated by comprehensive molecular dynamics simulations. I am somewhat surprised to see that MLFFs were not used for the simulations given the author's excellent work in this area but I do not feel this has a significant impact on the overall findings or validation. I have no hesitation in recommending the publication of this work as it is likely to be of significance to the field and indeed other related fields.

Response: We greatly appreciate the Reviewer's positive evaluations of our findings, the appreciation for our framework, and the support for publication.

Following the suggestion of this Reviewer as well as that of Reviewer #2, we have conducted new molecular dynamics simulations of 48 other glass materials using a recently developed machine learning force field (https://doi.org/10.1002/aenm.202503813). Based on this, we test and validate the capabilities of the max-flow method over a much larger range of inorganic glass systems using this more accurate and generable machine learning force field (see Response Figure R1 below). We refer to the responses to the comments of Reviewer #2 below for a detailed description of these additions.

Reviewer #2 (Remarks to the Author):

The manuscript presents an original and interesting approach to predicting ionic conductivity in glassy solid-state electrolytes by employing a novel adaptation of the max-flow algorithm on graph representations of static atomic structures. By leveraging this graph-theoretic framework, the authors claim to offer a method that circumvents the need for extensive molecular dynamics simulations, which are often time- and resource-intensive. The methodology is innovative and seems to provide a generalizable and computationally efficient alternative for screening candidate electrolyte materials across different chemistries. The correlation between predicted maximum flow and simulated ion diffusivity is examined across a few glass families, underscoring the potential of this method in materials discovery.

Response: We thank the Reviewer for their kind words and appreciation of the originality of our proposed method. We also appreciate all the constructive comments below.

While the proposed method is original and the results seem promising, the manuscript would benefit from a more thorough contextualization of the approach with respect to existing state-of-the-art methodologies in the field. In particular, the potential impact of the work remains somewhat unclear due to the lack of a direct, quantitative comparison with other established techniques for evaluating ionic conductivity in disordered systems. Additionally, certain assumptions, such as the exclusion of network atom mobility and the universality of backbone-based representations, require further clarification and discussion. These aspects are detailed in the following comments and should be addressed to better convey the broader applicability and limitations of the approach. Moreover, given the technical depth and methodological focus of the study, the work may be more appropriately targeted to a specialized journal such as NPJ Computational Materials, where its computational novelty and materials-science relevance can be fully appreciated.

Response: We acknowledge the Reviewer's point on having a deeper discussion of existing methodologies in the field and greater elaboration of the assumptions and validity hereof. Other Reviewers have also pointed to some of these shortcomings in their responses. We have critically tried to address this in the following responses and have accordingly made significant changes to the revised manuscript.

1/ In the introduction, the authors briefly mention the use of the Bond Valence method to study ionic diffusion in solids, amongst other more computationally intensive approaches. However, several other established or emerging approaches aimed at predicting ionic conductivity (particularly those designed to reduce computational cost while maximizing predictive accuracy) are not adequately discussed. For example, Refs. [DOI: 10.1063/1.4960790], [DOI: 10.1038/s41524-023-01051-2], [DOI: 10.1038/s41524-022-00877-6], [DOI: 10.1038/s41597-020-0491-x], [DOI: 10.1103/PhysRevMaterials.2.065405],and [DOI: 10.1063/1.4960790] offer alternative frameworks that share similar goals with the present work and should be acknowledged. A critical comparison between these methods and the proposed max-flow framework is missing and would be valuable to properly contextualize the contribution. Specifically, it remains unclear how the current approach compares in terms of predictive performance, computational efficiency, and applicability across different material systems. A more comprehensive discussion that highlights the strengths and limitations of the proposed methodology relative to existing techniques is essential.

Response: We agree that it would be valuable to include a more thorough comparison to other existing methods. As suggested by the Reviewer, we have thus included such comparison to other established as well as emerging methods for predicting ionic conductivity, as well as added the suggested references to the revised manuscript (see pp. 2-3 in the revised manuscript). We note that existing methodologies have mainly focused on much simpler crystalline materials with inherently more well-defined diffusion pathways. This is effectively what makes, e.g., the nudged-elastic-band or topological descriptions of electron densities feasible. In comparison, amorphous materials such as glasses feature a much broader diffusion space, requiring much larger simulation cells to reliably describe the diffusion process and make quantitative comparisons as function of, e.g., glass composition and temperature. This makes the application of these prior descriptions infeasible in the case of glasses and, in turn, requires the development of new methods, such as the present max-flow method.

To better assess the prediction performance of the proposed approach, we have performed additional simulations to include a larger range of glass compositions, covering a range of both oxides and sulfides with sodium and lithium as the mobile cations (in total 48 new

compositions). As also noted in the response to Reviewer #1 above, these new simulations were performed using a recent machine learning force field developed specifically for multicomponent glasses (https://doi.org/10.1002/aenm.202503813). As such, we have greatly extended the number of studied compositions compared to the previous version. Furthermore, we now provide the data as a normalized flow value (relative to system box area), i.e., the obtained results are now invariant of the specific system size being simulated.

As shown in Response Figure R1 below, we generally find the predictability to be best in the case of sulfide glasses, as oxide glasses (especially those with low conductivity) exhibit a large spread in their calculated max flow. This figure is included as an updated Figure 4 in the revised manuscript, and we have also added the related discussion in the text as well as included the full list of compositions in the new Supplementary Table 1. The larger spread in the data (compared to that in the original Figure 4) might be due to differences in the ionic transport mechanisms between the widely different glass compositions, but importantly we find that the method works well for the high-conductivity sulfide systems (Response Fig. R1a) across both sodium and lithium ions (Response Fig. R1b), i.e., those with a high calculated max-flow consistently feature high ionic conductivity. This confirms the method's applicability for prediction of ionic conductivity in this important family of glass electrolytes. We have also updated the title and abstract to reflect this. Regarding the oxide systems, we speculate that the poor predictability for these glasses may a results of oxygen's smaller ionic radii but similar charge as sulfur, creating more directionality in local-bonding between oxygen and the fast moving Na^+ and Li^+ ions. This, in turn, may create a stronger correlation between diffusion and the unique local environment of the ion compared to the more ionic bonding of the sulfides.

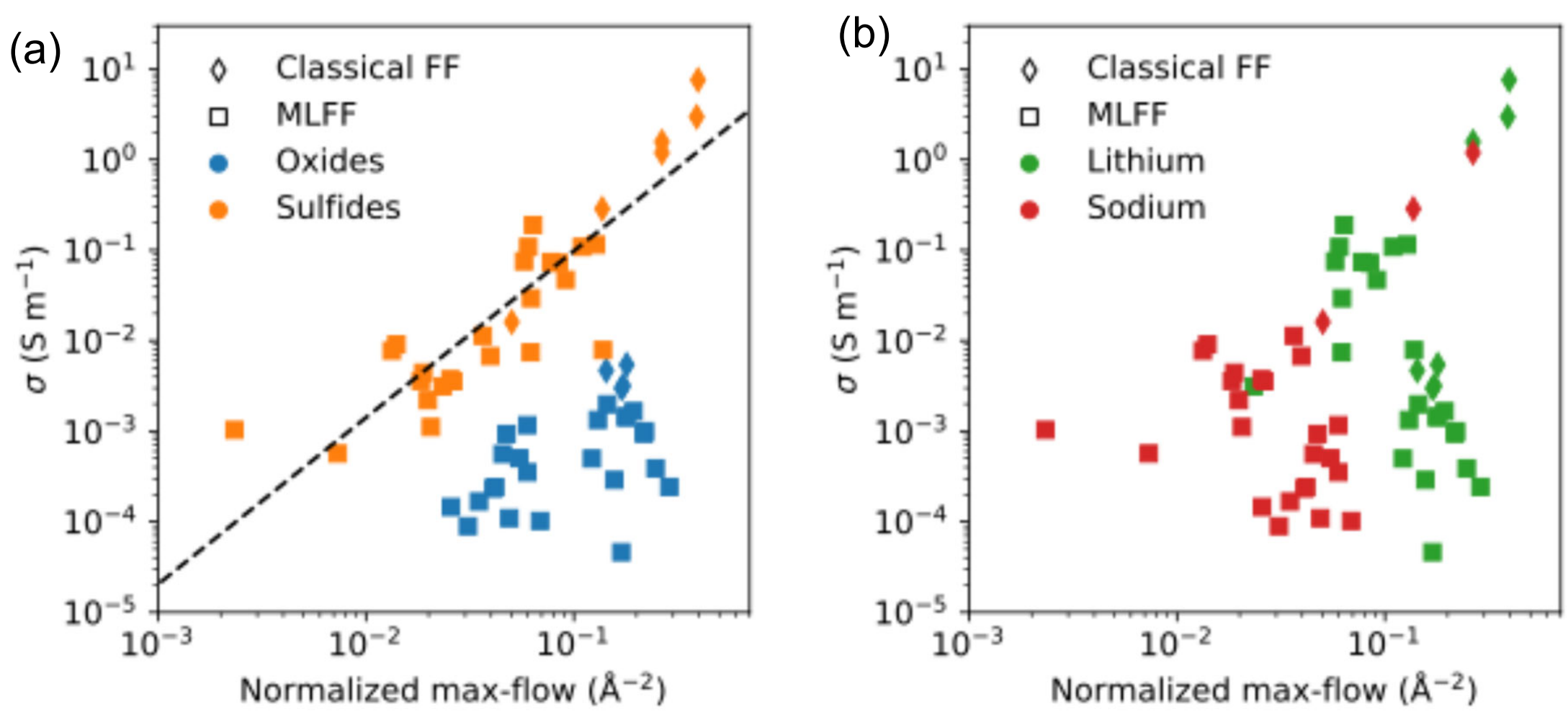

Response Figure R1. *Calculated room temperature ionic conductivity of glasses plotted as a function of normalized max-flow as calculated from the static glass structures. Results are categorized based on (a) glass family [blue: oxides; orange: sulfides] and (b) diffusing ion [green: lithium; red: sodium]. ◊ denotes glasses simulated by a classical force field and ☐ denotes glasses simulated by a machine-learning force field (MLFF). We refer to the Methods section for a detailed description of this distinction. Note that the axes of the plot are on a logarithmic scale and the black dashed line in panel (a) represents a guide for the eye.*

Regarding the comment about computational efficiency, we acknowledge that a more thorough discussion would be useful. In the revised manuscript and Supplementary Information, we have

thus included a dedicated discussion about this point, including a toy model for the geometric/topological part of the pipeline (see details in response to your comment no. 7 below). We note that this model should be considered as a conservative structural or near-worst-case complexity discussion, rather than as a direct proxy for wall-clock or CPU cost. In particular, the present implementation of the circular max-flow is solved through a linear-programming formulation on the compressed mapper graph, and the practical solution time can vary significantly from graph to graph depending on solver-level effects and graph structure. More generally, the elementary operations involved in MD and in the max-flow pipeline have very different constants and implementations. In the present simulations, this distinction is substantial, i.e., direct estimation of ionic conductivity from MD required about 1500 CPU-hours per composition, whereas the corresponding max-flow calculations required about 5 CPU-hours per composition, thereby being 2-3 orders of magnitude less costly than the raw computing of the property using MD. This confirms the practical applicability of our approach to accelerate the material discovery process of, e.g., high-conductivity glass electrolyte compositions for solid-state batteries. In the revised manuscript, we have included this analysis and discussion (p. 11 in the revised manuscript).

Finally, regarding the applicability of our method, and as described above, we have extended our simulations beyond the compositions of the original manuscript with 48 additional glass compositions. These glasses were designed to adhere to the composition of 1:2, 1:1, and 2:1 in their network-former to network-modifier ratio within a range of classical oxides and sulfides of relevance for ionic conduction (see Supplementary Table 1 in the Revised Supplementary Information for a full list of the compositions). We note how these compositions ensure a relatively static backbone (as the connectivity between network forming cations, e.g. Si, P, B, and Ge, is high, however varying with composition) and a mobile cation (Li^+ and Na^+). These new simulations have further validated the method applicability in the case of sulfide systems but also shown its limitations in the case of oxide systems (see Response Figure R1). Accordingly, we have thus modified the title, abstract, and descriptions about the general applicability of the method in the revised manuscript. We also note that we have not extended our tests to completely different materials classes (such as polymers), since such simulations and materials are outside both our scientific expertise as well as the scope of this study. We envision that such communities could apply our developed method and probe its predictability in a wider range of materials.

2/ The generation of the Fréchet mean and the definition of the spatial region accessible to ion diffusion are central steps in the proposed methodology. However, this process appears to rely on radial distribution functions, which require molecular dynamics simulations to compute. This raises a fundamental concern: a major motivation of the study is to avoid such computationally intensive simulations, yet the methodology seems to depend on them to define the diffusion space. It is unclear at what point this computational burden is genuinely reduced. If only short MD runs are used, there is a risk that the resulting void space is poorly defined or unrepresentative. Conversely, if long MD simulations are required to reliably characterize the diffusion space, one might question why not directly extract conventional transport metrics from the trajectories instead. This apparent contradiction should be addressed more clearly. The authors should specify the extent of MD required and provide a justification for how the proposed method reduces overall computational cost in practice, relative to standard MD-based analysis.

Response: Good points. The definition of the diffusion space is indeed a crucial step in the computation of the max-flow. The current predictions (used to generate the flows shown in

Figure 4 of the revised manuscript) rely on computations of diffusion spaces acquired from 100 snapshots distributed over a simulation time of 100 ps (i.e., every 1 ps). Further, the subsequent max-flow computation is rather small in computational cost (~5 CPU-hours per composition), as mentioned in our response to your comment no. 1 above.

In comparison, the estimation of ionic conductivity using long MD simulations require multiple nanoseconds of simulation and simulations at multiple temperatures to extrapolate the ionic conductivity to room temperature (300 K) in addition to the melt-quenching procedure. For accurate and thus generally more computationally demanding force fields (such as machine learning force fields), this means several hundreds or even thousands of CPU-hours of computational time per composition. We have tried to estimate the max-flow from single snapshots but, as the Reviewer points out as a risk, we consequently find larger variation in the flow and thus slightly worse predictability of ionic conductivity (where in comparison, the 100 ps (0.1 ns) simulation time at around 300 K appeared adequate to provide a good predictability). Ultimately, the difference in computational demand highly depends on the computational cost of the force field, but we assess the cost to be in the range of two to three order or magnitudes lower for the max-flow computation compared to computation of the ionic conductivity from MD simulations. To address the Reviewer's comments, we have added a brief discussion of the computational cost in the revised manuscript (see p. 10). We have also included more details in the revised Supplementary Information about the algorithm costs.

3/ Related to the previous point, it remains unclear how the proposed method would perform in scenarios where the mobility of the anionic framework (assumed to be relatively static in this work) or strong cation–anion coupling plays a significant role in ion transport. Such assumptions may limit the applicability of the method, particularly as recent studies increasingly highlight the importance of dynamic anion behavior and correlated cation–anion motion in highly conductive glassy systems, including halide-based electrolytes (see, for example, [DOI: 10.1002/anie.202315628], [DOI: 10.1002/adfm.202410509]). A more detailed discussion of how the method accounts for (or fails to capture) these complex dynamical effects is warranted. In particular, the authors should clarify the implications of their semi-static structural assumptions on predictive accuracy in systems where the backbone mobility or cation–anion interactions are non-negligible.

Response: We acknowledge that the method in its present form represents an assumption of a (largely) static backbone with an inherently mobile charge carrier ion (e.g. Li^+ and Na^+) within the free volume around the backbone-structure. This is implicitly defined in the definition of the backbone where in its current form we define an anion to be a part of the framework. While we are naturally aware of a range of more complex diffusion mechanisms in different systems, we would like to point out that many of these (including e.g. the 'Monkey Bar Mechanism' in 10.1002/anie.202315628 as highlighted by the Reviewer) are essentially also a backbone-charge carrier (Li^+) mechanism and that all charge carriers will have some interaction with the backbone in its diffusion through the structure (if not, it would be a gas).

Future testing should shed light on the applicability of the max-flow approach in even broader chemical systems (e.g. polymers), but such tests are beyond the scope of the present work. We have added a discussion of the above points in the revised manuscript (see pp. 10-11).

4/ The graph-based representation assumes that the weights on the edges reflect the maximum number of ions that can flow through each edge, effectively treating the conduction network as a capacity-limited flow system. However, this assumption may be problematic, as it

oversimplifies the physical nature of ion diffusion. In reality, the viability of a diffusion path is governed not only by geometric constraints but also by energy barriers, which can vary significantly across the structure and do not necessarily correlate with the number of ions that can theoretically pass through a given region. This raises concerns about whether the edge weights in the max-flow model accurately capture the true transport limitations within the material. The potential disconnect between geometric flow capacity and energy barrier-driven diffusion could result in misleading conclusions about conductivity. This conceptual issue should be addressed more thoroughly. The authors are encouraged to clarify how, or to what extent, energetic factors are implicitly or explicitly accounted for in their model, and to discuss the limitations of using purely topological or geometric criteria to infer ion mobility.

Response: We acknowledge the concerns of the Reviewer regarding the universality of the proposed method given the lack of explicitly defined energetic barriers. Indeed, this is evident from the much larger dataset now studied (see Response Figure R1 above). We also agree that the max-flow model does not attempt to reproduce the full dynamics of ion diffusion. Rather, in the present framework, the local description of the void space is already indirectly energy-informed through the effective radii used to define the excluded region around the backbone atoms, since these radii are extracted from molecular dynamics and therefore reflect the local ion-backbone interactions. Once this local description has been fixed, the remaining part of the method is geometric/topological in nature and enables quantification of how the resulting free volume is connected across the structure.

In this sense, our present results suggest, particularly for the sulfides studied herein, that the local chemically informed definition of the void space is a main driver of the diffusion process, while the subsequent prediction is governed by the geometry and connectivity of that void space. We view this as an empirical observation supported by the present data, rather than as a complete theory, and a deeper theoretical analysis of this chemical/local vs. geometric/global decomposition would be an interesting direction of research in future work. We added a brief discussion of the above points in the revised manuscript (see p. 9-11) and toned down statements regarding the general applicability of the method.

Furthermore, we note how the current version of the method represents the early stages of a wider implementation of the method for predicting ionic conductivity and even in this case provides good predictability of the ionic conductivity in the case of both lithium and sodium sulfide glasses. Future improvements could include more possibilities to "fine-tune" the weights of the max-flow computation to obtain even higher predictability. Ultimately, while such improvement has not yet been implemented, the computational framework allows for this implementation and given the fully open-source nature of the code, we envision this will be done in future work in the field.

5/ The results shown in Figure 4 demonstrate encouraging qualitative agreement between the predicted maximum flow values and the observed trends in ionic diffusivity across different material families. However, given the small dataset, the quantitative predictive power of the method remains unclear. Specifically, can the max-flow values be systematically calibrated or scaled to yield quantitative predictions of ionic conductivity or diffusivity, rather than just relative rankings? Addressing this question would significantly enhance the utility of the approach for practical materials screening. A discussion on whether such a calibration is feasible (either globally or within specific chemical families) and what limitations or uncertainties might be involved, would be valuable. Furthermore, exploring the possibility of

generalizing this framework to allow for predictive simulations across a broader range of systems could strengthen the impact and applicability of the method.

Response: To address this comment and as already noted in responses above, we have conducted a series of new simulations using a recent machine-learning based method developed in our group (https://doi.org/10.1002/aenm.202503813). In detail, we have added 48 new glass compositions to the previous Figure 4 with a range of new oxide and sulfide systems with both Li^+ and Na^+ as mobile cations (Response Figure R1). Further, we have now implemented an area-normalization of the max-flow, which ensures general applicability due to how the method is now invariant of the box size. Based on these new data, we show that a simple quantitative calibration is feasible in the case of sulfide glass electrolytes. That is, the plot shows room temperature ionic conductivity as a function of normalized max-flow, displayed on logarithmic scales (Response Figure R1). We observe that high-conductivity sulfide systems follow a clear increasing trend in this representation. In other words, the data in the revised manuscript for this glass family suggests that a simple model, relating normalized max-flow to log(σ), is a natural first quantitative calibration, and similarly for ionic diffusivity.

The data for the oxide systems highlight that a broader calibration study is needed to enable a universal predictive law across all glass compositions and material classes. Nevertheless, the updated results in the revised manuscript support the idea that max-flow is not only a ranking parameter but can also serve as the basis for simple quantitative predictions across both sodium and lithium charge carriers. We have added a new section to our Discussion section in the Revised manuscript (see pp. 7 and 9-10) to elaborate on the above points.

6/ With respect to the glass structures, while supplementary figure 16 shows good agreement between the computed and reference structures, this level of consistency does not appear to generalize across all systems. In particular, supplementary figures 17 and 18 reveal noticeable discrepancies, especially in the position and relative intensity of certain features. These differences raise questions about the robustness and transferability of the structural representation across different material families.

Response: We agree with the Reviewer that the computed and reference-structures do not perfectly agree. We infer how such disagreement is somewhat common in the simulation of glass materials and inherently stem from the problem of having a description of the potential-energy-landscape mimicking reality as well as the shortcoming of simulations only being able to simulate few nanoseconds compared to multiple seconds in reality. While a good agreement between simulations and experiments is of course important in the actual implementation and application of the method, we note that in the present work all correlations are established between the ionic conductivity and the max-flow, both computed from the same MD-simulated structure. This leaves no requirement for direct agreement between simulated and experimental glass structures. That is, it does not have any consequences for the model validation.

To address the Reviewer's comment in the revised manuscript, we have rephrased the description of the glass materials to now state that they are in "reasonable agreement" (see p. 12) but we have also noted that the lack of perfect agreement does not impair the claimed correlations (see p. 7). Finally, we also note that with the expected advancements in available datasets, algorithms, and computational resources in the coming years, machine learning force field simulations are likely to become increasingly accurate in their descriptions of glass structures. Such advancements will directly benefit the present methodology.

7/ Please provide more details regarding the computational cost and scalability of the proposed max-flow algorithm, particularly with respect to system size and complexity. Understanding how the algorithm performs as the number of atoms or the size of the simulation cell increases is important for assessing its practical applicability in large-scale screening efforts. Information such as computational time, memory usage, and parallelizability would help in this regard.

Response: The methodology presented in this work should be viewed as a proof-of-concept pipeline rather than as a final production-ready predictor. The emphasis of the present paper is to show that a max-flow-based geometric construction can be used to predict ionic conductivity of simulated glass structures through a procedure that also retains a clear geometric interpretation of how the prediction is produced. As also outlined in the manuscript, the predictor is obtained by constructing a geometric model of the void space, compressing it into a weighted mapper graph, and then computing a circular max-flow that can be interpreted in terms of the number and size of transport channels available to the diffusing ions. In this sense, the method is not just a black-box predictor, but a concrete geometric realization of the prediction mechanism.

Conceptually, the pipeline has two distinct components. The first one is chemical, i.e., one needs a fixed backbone together with effective radii r_j describing how closely the diffusing ions approach each type of backbone atom. The second component is geometric/topological, i.e., once the backbone point cloud and the radii have been specified, the subsequent construction of the void space, its grid approximation, the mapper graph, and the circular max-flow computation are agnostic to the detailed chemistry of the system, except through the radii passed to the geometric stage. These two components therefore pose different challenges and should be analyzed separately.

From this viewpoint, the main open challenge on the chemical side is local, i.e., predicting the radii r_j efficiently from local atomic environments, without having to simulate the full long-time diffusion process. In the new presented implementation, we achieve this information from standard short sampling (100 ps) of the glass system at room temperature, by measuring the onset of the first peak of the ion-backbone radial distribution function and using these to define the effective excluded region around the averaged backbone atoms. Ideally the radii of atoms would be defined on an atom-by-atom basis from the minimal observed ion-backbone distances observed across a sufficiently long sampling of the system. However, upon further examination we have found this approach to depend heavily on the sampling time as the radii of individual atoms does not converge even upon sampling up to 10 ns at 300 K. Hence, we have switched to the described approach of assigning a common radii to all atoms of a given type based on the onset of the first peak of the RDF, which is found to not depend heavily on sampling time. This has now been updated in the revised Supplementary Information (see Supplementary Methods section 4).

The final form we envision for the method is a pipeline that takes as input one or few snapshots together with local predictions of the per atom radii and then applies the geometric/topological part of the construction. However, developing and validating this final version of the pipeline lies beyond the scope of the present work given its already technically dense and broad scope, combined with the complexity of how to define per atom radii without long sampling. We therefore leave a systematic investigation of this fully local predictive setting for future work.

In the remainder of this response, we therefore focus exclusively on the second part of the methodology, namely the geometric/topological stage. That is, we assume as input a backbone point cloud in the periodic simulation box together with the associated radii, and we analyze the computational complexity of the pipeline built on this input. We do so at two levels. First, we discuss the cost of each step of the algorithm in general terms. Second, we connect these steps through a simple toy model, with the goal of relating the size of the mapper graph and the overall cost to coarse geometric characteristics of the input configuration.

Finally, we stress that several important questions remain open at this stage. First, the choice of the grid size P is treated in the present work through empirical validation, namely max-flow stability under increasing grid size and consistency with projected ion paths, rather than through a predictive a priori criterion. Developing a principled grid-selection strategy is therefore a natural next step, i.e., the toy parameter $w_{\min}$ introduced below should be read as a first illustration of the type of geometric quantity that one might hope to use for this purpose. Second, although the size of the simulated materials is validated in a principled practical way in the present study, and the underlying structures are also chemically validated, we believe that a more theoretical analysis could lead to useful heuristics for size selection and for related questions, such as anisotropy and finite-size effects. In this direction, the comparison of the flows obtained along the three coordinate axes should be viewed as a first practical diagnostic rather than as a complete theory. More broadly, the toy complexity model introduced below should be viewed only as a first indication that such an analysis is feasible. A more formal and more chemically informed model, capable of incorporating finer structural information and yielding better heuristics for parameters, such as the grid resolution and the relevant system size, would be a natural direction for future work.

General computational complexity of the max-flow method: Under the standing assumption that the backbone point cloud and the radii are given as input, the dominant computational cost of the geometric/topological part of the max-flow pipeline arises from discretizing the periodic void space on a $P \times P \times P$ grid and testing membership against the thickened backbone; see the description of the void-space graph approximation and mapper construction in the manuscript and Supplementary Information. Let $\mathcal{B}$ denote the set of backbone atoms, and let

$$\#G := P^3$$

be the total number of grid points, consistently with the notation of the Supplementary Information.

A direct implementation of the void-space test computes, for each grid point, its toroidal distance to all backbone atoms and checks whether the point lies outside the chosen thickening radius. In the absence of further spatial acceleration, this step has complexity

$$O(\#G\,\#\mathcal{B}) = O(P^3\,\#\mathcal{B}).$$

Once the retained grid points have been identified, let $N \leq \#G$ denote their number. The construction of the retained grid graph is then linear in N, since each retained point is connected only to retained points in its local neighborhood, so the number of grid-graph edges is $O(N)$ up to a fixed constant factor. Moreover, connected components of an undirected graph can be computed in time $O(\#V + \#E)$ by repeated breadth-first or depth-first search; see, e.g. https://mitpress.mit.edu/9780262046305/introduction-to-algorithms/ and https://doi.org/10.1137/0201010. Hence, each connected-components computation appearing

in the mapper construction is linear in the size of the corresponding subgraph. The subsequent construction of the mapper graph is likewise linear in the size of the retained graph, i.e., the cover along one coordinate has constant overlap, each retained point belongs to at most two cover elements, and connected components are computed on subgraphs of the retained grid graph. Thus, both the computation of the mapper graph and the assignment of its edge capacities are of order $O(N)$. The same holds for the assignment of edge capacities, since capacities are computed from overlap cardinalities between adjacent cover elements and therefore require only a linear pass through the relevant retained-grid incidences. Hence the full compression stage, from the retained grid graph to the weighted mapper graph, has complexity $O(N)$.

This step is also the key dimensionality-reduction stage of the pipeline, as it replaces a graph with around 10^7 grid points by a much smaller weighted graph that still records the channel and slice connectivity relevant for the circular max-flow.

The final optimization step is not performed on the full retained grid graph, but on the much smaller weighted mapper graph. If $M = (V, E)$ denotes the weighted mapper graph, then the circular max-flow problem is a linear program whose size is controlled by $\#V$ and $\#E$, and therefore has complexity polynomial in the mapper-graph size:

$$\mathrm{poly}(\#V, \#E).$$

Accordingly, the overall complexity of the geometric/topological stage may be summarized as

$$O(P^3\,\#\mathcal{B}) + O(N) + \mathrm{poly}(\#V, \#E),$$

In all cases, the grid-based void-space discretization typically represents the dominant bottleneck, while the final optimization is carried out only on the compressed mapper graph.

Practical note: The current implementation was primarily designed to validate the methodology and its predictive power, rather than to optimize each individual step to its best possible asymptotic or practical computational cost. In particular, the current choice of P is made empirically through stability and path-recovery tests, and some stages, most notably the mapper graph construction, could likely be improved substantially by more specialized data structures or other implementation-level optimizations.

A toy complexity model from the atomic configuration: In the following, we outline the toy complexity model relating the cost of the geometric/topological part of the pipeline to basic geometric features of the underlying periodic atomic configuration. As mentioned above, the goal is not to prove a rigorous worst-case theorem, but rather to provide a simple scaling picture explaining why the mapper construction can drastically reduce the size of the graph on which the final max-flow problem is solved. Throughout, the notation

$$A \asymp B$$

means that A and B are comparable up to multiplicative constants, while $O(\cdot)$ denotes the usual asymptotic upper bound up to a constant factor.

***1) Geometric setting*.**

Following the description in the Supplementary Methods, we start from a fixed-point cloud given by the averaged backbone in the simulation box with periodic boundary conditions, viewed as a flat 3-torus. Let $\mathcal{B}$ denote the set of backbone atoms. For each backbone atom $\bar{b}_j$,

one defines a radius r_j from the minimal observed distance to the diffusing ions across time, and then considers the resulting void space

$$X := \mathbb{T}^3 \setminus \bigcup_j \overline{B(\bar{b}_j, r_j)}.$$

This is the region in which the conducting ions are assumed to move. The void space is then discretized by a regular grid of size P in each coordinate, yielding a grid G and its graph approximation X_G. The corresponding weighted mapper graph is denoted by

$$M = (V, E),$$

with capacity function

$$C: E \to \mathbb{N}.$$

This matches the notation introduced in Supplementary Methods sections 4-6.

2) Choice of the grid scale.

A natural quantity controlling the required resolution is the size of the smallest relevant void feature that should still be captured by the grid. We denote this heuristic scale by

$$w_{\min} > 0,$$

and interpret it as the width of the narrowest transport-relevant channel or neck in the void space. A toy resolution criterion is then to choose the grid step δ so that

$$\delta \leq \alpha\, w_{\min}, \qquad \alpha \in (0,1).$$

Equivalently, if the side lengths of the simulation box are of order L, then one expects

$$P \asymp \frac{L}{\delta} \gtrsim \frac{L}{\alpha w_{\min}}.$$

For simplicity, we assume here that all side lengths are comparable and write L^3 as a proxy for the box volume. We emphasize again that this is not the grid-selection rule used in the present paper, i.e., here P is chosen empirically through numerical stability and path-recovery diagnostics. Rather, the present discussion should be read as a possible direction for replacing that empirical choice by an *a priori* geometric criterion in future work.

3) Size of the retained graph X_G.

Let

$$\phi := \frac{\mathrm{Vol}(X)}{\mathrm{Vol}(\mathbb{T}^3)} \in (0,1)$$

denote the void fraction. Since the full grid contains

$$\#G = P^3$$

points, the number of retained grid points in X_G is heuristically of order

$$\#X_G \asymp \phi P^3.$$

Using the above choice of resolution, this becomes

$$\#X_G \asymp \phi(\frac{L}{\alpha w_{\min}})^3.$$

Likewise, the number of edges of X_G is linear in the number of its vertices, i.e., the retained graph remains of cubic order in the inverse grid scale.

4) A topological heuristic for the size of the mapper graph.

We now estimate the size of the mapper graph $M = (V, E)$. In Supplementary Methods section 5, the mapper construction proceeds by fixing one coordinate direction, considering overlapping intervals made of consecutive grid levels, building the subgraphs X_G^k, and then taking their path connected components as vertices of the mapper graph. Edges record non-empty intersections of such components in adjacent overlaps, and capacities are given by the cardinalities of these intersections.

Let C_k denote the number of path connected components of the subgraph X_G^k. Then, at the level of scaling,

$$\#V \asymp \sum_k C_k,$$

while $\#E$ is of the same order as the total number of non-empty overlap incidences between components in consecutive slices.
To obtain a heuristic control on C_k, let O_k denote the union of the inflated backbone balls intersected with the k-th thin slice, and let V_k denote the corresponding void region in that slice. Then C_k is the discrete counterpart of $\beta_0(V_k)$, where β_0 is the 0-th Betti number, that is, the number of connected components. Likewise, $\beta_1(O_k)$ is the 1-st Betti number, which qualitatively counts the number of independent loops or "holes" in the sliced obstacle set.

In an effectively two-dimensional thin-slice regime, Alexander duality gives, for a sufficiently regular compact set $A \subset \mathbb{R}^2$,

$$\beta_0(\mathbb{R}^2 \backslash A) = \beta_1(A) + 1;$$

see, for instance see https://pi.math.cornell.edu/~hatcher/AT/AT.pdf. A visual interpretation is given in Response Figure R2a below. This says that, in two dimensions, the number of connected components of the complement is exactly controlled by the number of independent loops in the obstacle set. In our setting each slice is periodic, so strictly speaking it is closer to a thin 2-torus than to the plane or the sphere. However, for a very thin slice the same picture remains a reasonable guiding principle: the connectivity of the void region is still expected to be governed by the loop complexity of the sliced obstacle set, up to global corrections coming from periodicity. This motivates the heuristic estimate

$$C_k \lesssim 1 + \beta_1(O_k).$$

In other words, the slice complexity is governed not merely by the number of nearby atoms, but more fundamentally by the topological complexity of the excluded region inside the slice.

5) From topological complexity to atom count.

The topological heuristic above can be combined with a crude local-complexity assumption. Let N_k denote the number of backbone atoms whose inflated balls intersect the k-th slice. In the effectively two-dimensional thin-slice regime, it is reasonable to assume that each additional intersecting atom creates at most $O(1)$ new independent loops in the sliced obstacle set, so that

$$\beta_1(O_k) = O(N_k).$$

This assumption is tied to the fact that the slice is modeled as essentially two-dimensional. A simple grid cartoon illustrating this phenomenon is shown in Response Figure 2b below: adding one local obstacle can create only a bounded number of new elementary loops. Consequently,

$$C_k = O(1 + N_k).$$

Hence atom count acts only as a coarse proxy for the homological complexity of the sliced obstacle set. If the atoms are distributed with average density,

$$\rho_a := \frac{\#\mathcal{B}}{L^3}$$

and the slice thickness is of order δ then one expects

$$N_k \asymp \rho_a L^2 \delta$$

and therefore:

$$C_k = O(1 + \rho_a L^2 \delta).$$

6) Heuristic size of the mapper graph.

Summing over the $P \asymp L/\delta$ slices gives

$$\#V = O(P(1 + \rho_a L^2 \delta)).$$

Using $P \asymp L/\delta$ and $\rho_a = \#\mathcal{B}/L^3$, we obtain

$$P(1 + \rho_a L^2 \delta) \asymp \frac{L}{\delta}\left(1 + \frac{\#\mathcal{B}}{L^3} L^2 \delta\right) = \frac{L}{\delta} + \frac{\#\mathcal{B}}{L}.$$

Since L is treated here as part of the ambient geometric scale, this yields the simplified estimate

$$\#V = O\left(\frac{L}{\delta} + \#\mathcal{B}\right),$$

and similarly

$$\#E = O\left(\frac{L}{\delta} + \#\mathcal{B}\right).$$

Under the resolution choice

$$\delta \asymp \alpha w_{\min},$$

this becomes

$$\#V, \#E = O\left(\frac{L}{\alpha w_{\min}} + \#\mathcal{B}\right).$$

7) Compression ratio.

Within this toy model, the retained graph has size

$$\#X_G \asymp \phi(\frac{L}{\alpha w_{\min}})^3,$$

whereas the mapper graph has size

$$\#V, \#E = O\left(\frac{L}{\alpha w_{\min}} + \#\mathcal{B}\right).$$

Therefore, the ratio

$$\frac{\#X_G}{\#V}$$

can be interpreted heuristically as the average number of retained grid vertices that are compressed into one mapper vertex. When the term $\#\mathcal{B}$ is of lower order than $L/(\alpha w_{\min})$, this ratio is of order

$$\frac{\#X_G}{\#V} \asymp \phi(\frac{L}{\alpha w_{\min}})^2.$$

Thus, the retained graph reflects a three-dimensional discretization of the void space and scales cubically in the inverse grid spacing, while the mapper graph may scale much more mildly, essentially like the number of slices corrected by a term depending on the local obstacle complexity.

8) Implications for the overall complexity.

Under the present input model, the most expensive stage in a direct implementation is typically the construction of the discretized void space. Indeed, if one tests membership of each grid point against all backbone atoms, this step has cost

$$O(\#\mathcal{B}\, P^3) = O\left(\#\mathcal{B}(\frac{L}{\alpha w_{\min}})^3\right).$$

Once X_G has been built, the graph construction and the connected-components computations involved in the mapper step are linear in the size of the relevant subgraphs. The final circular max-flow is then solved on the weighted mapper graph $M = (V, E)$, and can be treated algorithmically as a classical min-cost circulation problem on a constant-size augmentation of that graph. Hence the toy end-to-end complexity model becomes

$$O\left(\#\mathcal{B}(\frac{L}{\alpha w_{\min}})^3\right) + O\left((\frac{L}{\alpha w_{\min}} + \#\mathcal{B})\right)^3 \;+\; \text{poly}\left(\frac{L}{\alpha w_{\min}} + \#\mathcal{B}\right).$$

where the second term is obtained by inserting the estimate $\#\text{V}, \#\text{E} = O(\frac{L}{\alpha w_{\min}} + \#\mathcal{B})\#$ into the reference min-cost-flow complexity $O(\#V^2 \#E\; log \#V)$.

Thus, at the level of this toy model, the essential conclusion is that the optimization is performed only on the compressed mapper graph, and its cost is polynomial in the effective graph size. In other words, after compression to the mapper graph, the optimization is expected to scale polynomially in the effective one-dimensional slice complexity, rather than in the full three-dimensional grid size.

Interpretation and limitations: The purpose of this toy model is not numerical accuracy, but rather to explain the different scaling regimes of the two graph constructions. The graph X_G represents a three-dimensional discretization of the void space and therefore has a size that is cubic in the inverse grid scale. By contrast, the mapper graph records how path connected components evolve across overlapping slices and may therefore be much smaller. At the same time, this toy model should not be interpreted as a direct predictor of CPU time. In the actual implementation, the circular max-flow is solved througha linear-programming formulation on the compressed mapper graph, so the practi-cal runtime depends not only on the asymptotic graph size but also on solver-level effects such as sparsity, conditioning, and implementation details. As a consequence, two graphs of comparable theoretical size may still have quite different actual solution times. For this reason, the present complexity discussion should be read as a conservative structural model rather than as a quantitative runtime law. Finally, this discussion concerns only the geometric/topological stage of the pipeline. The chemical problem of predicting the radii remains outside the scope of the present complexity analysis and is better viewed as a separate local learning or regression task. Likewise, the present paper does not yet resolve the principled choice of the grid size, the identification of quantities such as wmin, or a more theoretical treatment of system-size effects in the simulated materials. These are natural directions for future work if one aims to turn the present proof-of-concept methodology into a fully mature screening tool.

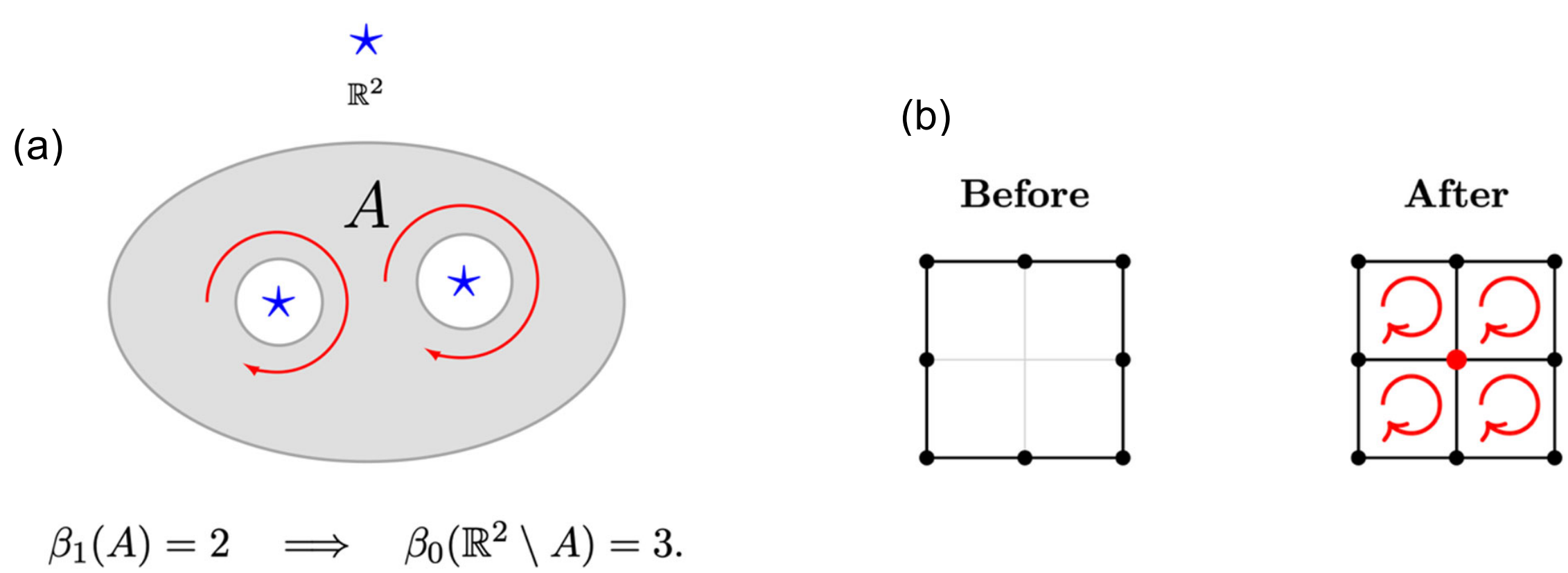

Response Figure R2. *(a) Planar Alexander-duality picture. The gray region is A. The two red curved arrows, drawn in A around the holes, indicate its two independent loops, while the three blue symbols mark the three connected components of $\mathbb{R}^2 \setminus A$: the two hole interiors and the unbounded exterior region. (b) Planar square-grid analogy. After adding the central occupied vertex, at most four new elementary square loops can appear around it, highlighted by the four red curved arrows in the "After" panel.*

We have included added the above discussion in the revised Supplementary Information and refer to it in the revised manuscript (see p. 6).

Reviewer #2 (Remarks on code availability):

The associated code provided at https://github.com/pego91/circular_max_flow has not been thoroughly evaluated in detail; however, from a general inspection, the repository appears to be reasonably well-organized. The directory structure seems clear, and the use of modular Python scripts facilitates readability and potential reuse. That said, it is recommended to explicitly add a detailed README file at the root (include project overview, installation instructions, usage examples, input/output formats, and expected results). Additionally, more comprehensive inline documentation and user-facing examples or tutorials would further enhance the accessibility and reproducibility of the workflow, particularly for researchers outside the immediate field.

Response: We thank the Reviewer for their careful evaluation of our code and the helpful comments. We agree that accessibility, documentation, and reproducibility are essential if the method is to be usable beyond readers already familiar with graph optimization and computational topology. In this respect, we would like to stress that a substantial part of our software effort has gone into integrating the circular max-flow approach into *Topological Material Analysis* (ToMA), a broader software project for the topological analysis of atomic and material configurations, rather than limiting the implementation to a standalone research repository. The current public software release is available on Zenodo and Github at http://doi.org/10.5281/zenodo.10781424, https://github.com/yossibokorbleile/TopologicalMaterialAnalysis; see also the documentation page at https://yossi.eu/toma/. Furthermore, we have added a more directly applicable cli script to the ToMA repository, which can be run using a single config .yaml file, along with an example on how to use this script on a toy structure.

We believe this integration directly addresses the Reviewer's concern about usability. While the standalone circular max-flow repository is intentionally modular and research-oriented, ToMA already provides a substantially more accessible interface for reproducing the workflow of the present paper. In particular, ToMA provides a graphical user interface through which users can load and inspect atomic configurations, visualize the corresponding structures, and access topological analyses and summaries without interacting directly with low-level graph-theoretic scripts. We view this integration as part of an ongoing effort to develop practical software infrastructure for topological analysis of atomic configurations and materials data, rather than as a one-off code release tied only to the present manuscript. We are also currently preparing a separate software-focused publication devoted specifically to ToMA and its broader capabilities.

In the revised manuscript, we have made this broader software context explicit and added clearer references to these resources, so that readers can more easily identify the most user-friendly route for reproducing the present results. Furthermore, we have added minimal working examples of max-flow predictions to the present work as part of the ToMA repository (http://doi.org/10.5281/zenodo.10781424, https://github.com/yossibokorbleile/TopologicalMaterialAnalysis). Finally, we have adapted the "Code Availability" section (see p. 13 in the revised manuscript) to reflect the above additions.

Reviewer #3 (Remarks to the Author):

This article presents a novel computational approach for modeling and predicting ionic conductivity in glassy solid-state electrolytes using max-flow methodology. The work addresses a significant challenge in battery research by proposing an approach that could accelerate the discovery of high-performance electrolyte materials. The authors successfully adapt graph theory concepts to glasses. The methodology is well-motivated by drawing parallels between ion transport and classical network flow problems.

Response: We greatly appreciate the Reviewer's comments on our work and positive appreciation of the introduced max-flow concept for the estimation of ionic conductivity.

The following comments can be made regarding this work:

On page 8, when discussing the difference between the calculated and experimental ionic conductivity values, discrepancies exist between mentioned values. However, the discussion of the potential reasons for this is insufficient and requires more details. A more detailed comparison with the MD simulation data should be included in the supplementary materials if possible.

Response: We acknowledge that there is a discrepancy in the experimental (from the literature) and simulated values of ionic conductivity. We note that such discrepancies are a very common problem in the field and ultimately are a consequence of the reliability of the atomistic force field and available computational resources (i.e., the resulting finite system size and time effects). Furthermore, some uncertainty is also introduced from the common Arrhenius extrapolation of diffusion constants from higher to lower temperatures, in which it is assumed that room temperature is includable. For a more detailed discussion on this, we refer the Reviewer to previous works, such as https://doi.org/10.1016/j.mtphys.2021.100463 and https://doi.org/10.1088/0034-4885/79/6/066504.

However, despite the discrepancies, we note that the developed correlation between the max-flow and ionic conductivity only relies on simulation data. That is, we do not rely on a direct agreement between the experimental and simulated data for the developed methodology and correlation in Figure 4 to be valid. As also noted in our response to comment no. 6 of Reviewer #2 above, we infer and acknowledge that for practical materials discovery, the reliability of the force field in capturing the accurate glass structure is required. With the expected advancements in available datasets, algorithms, and computational resources in the coming years, machine learning force field simulations are expected to become increasingly accurate in their descriptions of glass structures. Such advancements will directly benefit the present methodology.

We have included a brief discussion of the above points in the revised manuscript (see p. 9).

The caption for Supplementary Figure 6 in the supplementary materials should refer to Supplementary Figure 5 (which is an enlargement of Figure 2e from main text).

Response: We thank the reviewer for their careful reading. It has now been fixed in the revised Supplementary Materials.

After these minor revisions, the article can be recommended for publication in Nature Communications.

Response: We again thank the Reviewer for their positive appreciation of our work and we believe that the performed revisions of the manuscript now make it suitable for publication.

Reviewer #4 (Remarks to the Author):

This work presents an original methodology that adapts the max-flow algorithm, commonly used in continuum models for fluid and transport phenomena, to study ion transport in glassy inorganic solid electrolytes. The approach accounts for periodic boundary conditions (PBC) and uses flow network theory to quantify ion-conducting pathways from static atomic structures. This suggests that the max-flow metric could serve as a computationally efficient structural descriptor for predicting ionic conductivity in glassy SSEs. It is an interesting and novel contribution to method development.

Response: We thank the Reviewer for their kind words and appreciation of the originality of our proposed method.

However, its application is constrained by key assumptions and some other limitations listed below, which may hinder its accessibility, reproducibility, and broader adoption within molecular modelling community. I recommend that the work be considered in more specialised computational journal.

Response: We acknowledge that the initial submission had some shortcomings. For this reason, we have revised the manuscript according to yours and the other Reviewers' comments and believe that the revised manuscript is presenting an improved version of the work and that it is now suitable for publication in the present journal.

(1) The model assume that backbone is static, and ions motion follows Arrhenius relationship. This assumption is valid for many solid inorganic glassy systems below the Tg. However, it is not for a broad range of polymer electrolytes or hybrid systems where the backbone exhibits significant segmental motion near or above Tg. In such systems, backbones are coupled with ion transport and conductivity shows non-Arrhenius behaviour. The authors should discuss whether the proposed method remains valid or meaningful under such conditions.

Response: We agree that this is a valid concern and it was also raised by Reviewer #2 above (see our responses to their comments no. 1 and 3). To probe the max-flow for a larger chemical range of glass compositions, we have in the revised manuscript included 48 new samples with compositions with ratios of 1:2, 1:1 and 2:1 for common glass-formers and modifiers. In short, we find that sulfide systems show better predictability in its correlation between max-flow and ionic conductivity, as discussed in relation to Response Figure R1 above. Based on this, we argue that the max-flow method is particularly useful for these systems with high conductivity with flowing ions within the free-volume inside the structure. This is now reflected in the revised title, abstract, and throughout the main text.

We note that while the method has not been tested in other systems such as polymers, the correlation may to some extend still work as time averaging may provide a meaningful

description of the "average" backbone, which could correlate to, e.g., ionic conductivity. While interesting, such simulations would infer a whole new simulation setup and are therefore beyond the scope of the current work. We imagine that such testing could be introduced in future studies within the solid state ionics community. We do, however, note that in the present simulations, we have tested two types of anions (oxides and sulfides), finding good predictability particularly for sulfides. We infer that this is likely due to how the changing chemical interactions are inherently reflected by the backbone structure and thereby replicated in the max-flow, yet that the sulfide predictability is better due to a less directional and more ionic character of the backbone-ion interactions.

We have added a brief discussion on the above points in the revised manuscript (see pp. 9-11). We have also toned down some statements about the general applicability of the method across the revised manuscript, including in the title and abstract.

(2) The model predict ionic flux based purely on void space geometry, without incorporating energetic interactions between mobile ions and the surrounding matrix. For example, for two different polymer systems with identical void geometry, will the model predict the same conductivity?

Response: We thank the Reviewer for raising this important point. A similar concern was raised by Reviewer 2# above (see our response to their comment no. 4). Generally, atomic snapshots of two polymer systems of identical void geometries will indeed per-se produce a similar max-flow (and thereby a similar prediction of ionic conductivity). However, this should be interpreted carefully, because the void-space definition itself is not purely geometric in an abstract sense. That is, it is indeed largely chemistry-based through the effective radii extracted from the MD simulations. Thus, for two systems with the same backbone to yield the same void space in our framework, they would also need to display the same ion-backbone behavior in the MD data used to define that void space. In this sense, the method is geometric at the level of the final transport graph, but the construction of the admissible void region already incorporates chemical information.

Furthermore, while not implemented in the present work, the max-flow algorithm allows for adding weights to edges. We imagine that such weighing based on the local chemistry in vicinity of the edge would allow for a more complete description of the max-flows across even more material families and compositions, or it could even serve to "calibrate" the method to obtain improved predictability of the ionic conductivity. While such implementation is beyond the scope of the current work, which rather serves to introduce and validate the method in the case of glass materials, the open-source nature of the code allows any future user to modify the code according to a specific need or chemical system.

To address the Reviewer's comment, we have included these discussions in the revised manuscript (see pp. 6-7 and 10).

(3) While the max-flow framework is conceptually simple, its practical implementation requires familiarity with graph theory and network optimization — skills not commonly taught in traditional molecular modeling or materials simulation curricula. This may limit its accessibility to researchers without computational graph modeling experience, and highlights the need for well-documented tools and workflows to support broader application.

Response: We agree with the Reviewer that some assistance and guidance is needed to use the method for scientists who are not experts in graph theory, but we respectfully disagree that complete understanding is necessary to implement the said framework for practical applications. Practically all simulation software employs optimization strategies, which are not completely understood by all users.

However, to enhance the accessibility of the max-flow computation and address the Reviewer's comment, we have integrated the full workflow into the software code *Topological Material Analysis* (ToMA), which provides a significantly more accessible entry point than a standalone research code. In particular, ToMA offers a comparatively simple interface for loading structures, inspecting them, and reproducing the main steps of the analysis without requiring users to interact directly with low-level graph-theoretic code. We believe that this is the most practical way to make the framework reproducible and usable also for researchers who are not specialists in graph optimization, and we have made this broader software context more explicit in the revised manuscript. For more details, we also refer the Reviewer to our response to the "coda availability" comment of Reviewer #2 above. Furthermore, we have provided useful examples of usage as part of the ToMA repository (http://doi.org/10.5281/zenodo.10781424, https://github.com/yossibokorbleile/TopologicalMaterialAnalysis) and have accordingly revised the Code Availability section of the revised manuscript (see p. 13).

(4) Calculation of diffusion coefficients using KINISI package involves Bayesian linear regression and MCMC sampling, which offers uncertainty quantification but introduces additional complexity. Reproducing this workflow requires familiarity with probabilistic modeling and parameter tuning, which may be a barrier for users accustomed to classical linear MSD fitting approaches.

Response: We acknowledge that the KINISI package is more complex compared to regular linear fitting of MSDs, but we simultaneously acknowledge KINISI's great efforts in providing more statistically robust measurements of the MSD compared to regular MSD fitting. Also, KINISI provides an extremely well-documented webpage (https://kinisi.readthedocs.io/en/stable/) with detailed information on installation and usage as well as several examples of usage and is thus very accessible to the community. As such, we do not think that its usage impairs the presented results. That is, KINISI will effectively provide similar results as a well-averaged regular fitting of the MSD. To address the Reviewer's concern, we have added a note in the revised manuscript about the use of KINISI in comparison to other methods of estimating the diffusivity (see p. 13 of the revised manuscript).

Reviewer #4 (Remarks on code availability):

Python codes are available. However, no step-by-step instructions are available to advice readers how to run codes to reproduce this work. This is challenging for people without relevant background.

Response: In the revised manuscripts Code Availability section (see p. 13 in the revised manuscript), we have emphasized that the recommended route for reproducing and extending the present framework is through its integration in ToMA. Beyond the availability of the underlying Python code, this integration provides a more user-friendly environment, basic documentation, and minimal working examples, making it substantially easier to reproduce the results of the present paper and to adapt the workflow to related systems. We agree that further

software-oriented documentation will continue to improve accessibility, but we believe that the current ToMA integration already addresses the Reviewer's main concern regarding step-by-step reproducibility. For more details, we again refer the Reviewer to our response to the "code availability" comment of Reviewer #2 above.

Point-by-Point Response Letter

We thank the Reviewers for reading our manuscript again and for their additional comments. Detailed point-by-point responses to these comments are given below.

Reviewer #1:

The authors have fully addressed any minor comments I had. The manuscript is ready for publication.

Response: We thank the Reviewer for taking the time to go through our work again.

Reviewer #1 (Remarks on code availability):

The improvements in readability and application of the code should be much appreciated by the community.

Response: We thank the Reviewer for acknowledging these improvements.

Reviewer #2:

The authors' substantial efforts to revise the manuscript are noted and appreciated. They have significantly expanded the materials, analyses, and explanatory detail, and have successfully addressed all previous concerns. The manuscript seems now suitable for publication.

Response: We thank the Reviewer for taking the time to go through our work again and for acknowledging the improvements.

Reviewer #4:

The revised manuscript has improved substantially, particularly through the expanded dataset, the narrower focus on sulfide glass electrolytes, and the improved code-availability information. I have two minor suggestions that may help further clarify the scope and reproducibility of the revised work.

Response: We thank the Reviewer for taking the time to go through our work again and for acknowledging the improvements.

1. The manuscript should more clearly define when the max-flow descriptor is expected to be valid. In particular, it should state that the method is most appropriate when the host/network structure is effectively static on the ion-transport timescale, and that dynamically disordered systems such as polymer or hybrid electrolytes require separate validation.

Response: We agree with the Reviewer that the validity regime of the max-flow descriptor should be more explicitly defined in the manuscript. As suggested, we have therefore added text to clarify that the present validation is focused on inorganic glass electrolytes in which the

host/network structure is effectively static on the ion-transport timescale. We now also explicitly state that dynamically disordered systems, such as polymeric or hybrid electrolytes where ion-hosted network rearrangements may be coupled to ion transport, require separate validation before the max-flow descriptor can be applied (see p. 4 in the revised manuscript). We have further clarified this point in the "Maximum flow pipeline" section, where the fixed-backbone approximation is introduced.

Specifically, we now state that the descriptor is expected to be most meaningful when the backbone structure is effectively static on the ion-transport timescale, i.e., when a single time-averaged backbone provides a representative structural environment for ion migration (see p. 6 in the revised manuscript).

2. The authors acknowledge in the response letter that the current method is mainly based on void-space geometry/connectivity after the RDF-derived void definition is fixed. This limitation should also be stated explicitly in the manuscript. Systems with similar void connectivity but different ion-binding strengths or activation barriers may yield similar max-flow values unless those energetic differences are indirectly reflected in the static structure or RDF-derived void definition.

Response: Yes, we have revised the "Maximum flow pipeline" section to clarify that local ion-backbone interactions enter the present descriptor indirectly through the radial distribution function-derived definition of the excluded volume (see p. 6 in the revised manuscript). In addition, we have added a brief statement in the Discussion to make the implication of this approximation clear. Notably, we now state that systems with similar ion-accessible void connectivity but different ion-binding strengths or activation barriers may yield similar max-flow values unless those energetic differences are reflected in the static structure or in the radial distribution function-derived void definition (see p. 10 in the revised manuscript).
